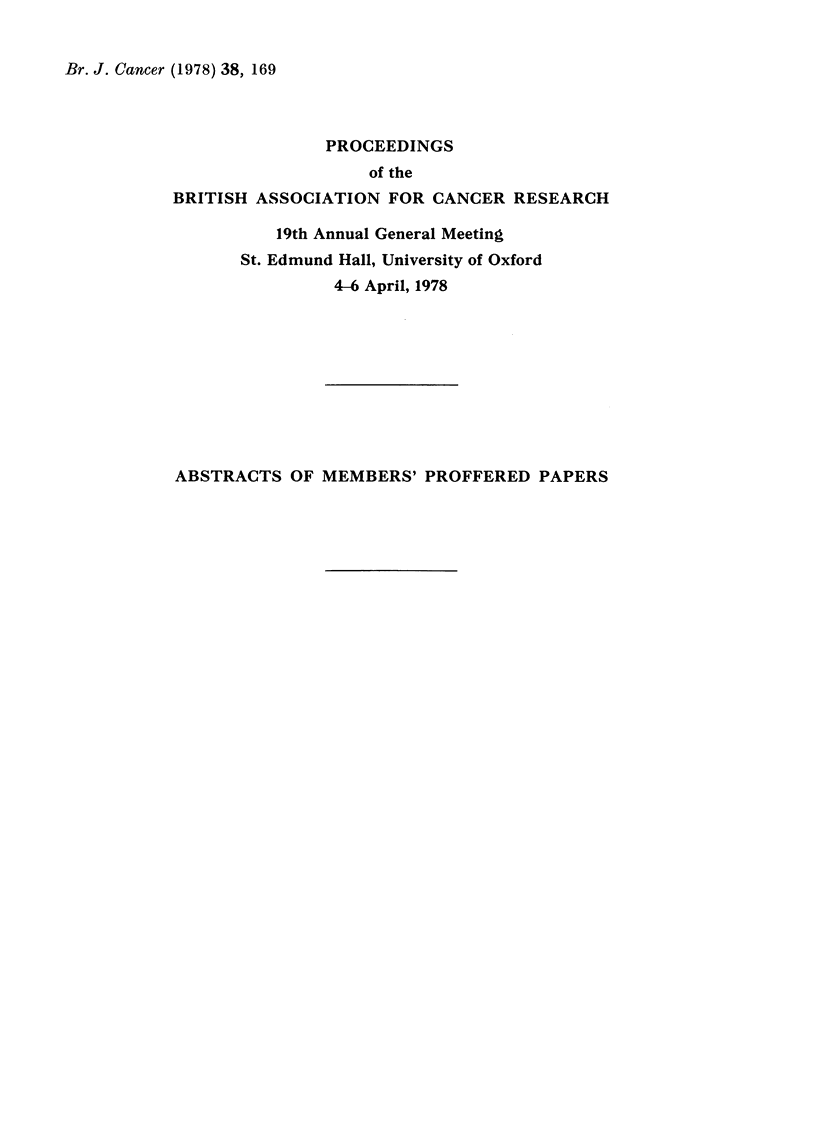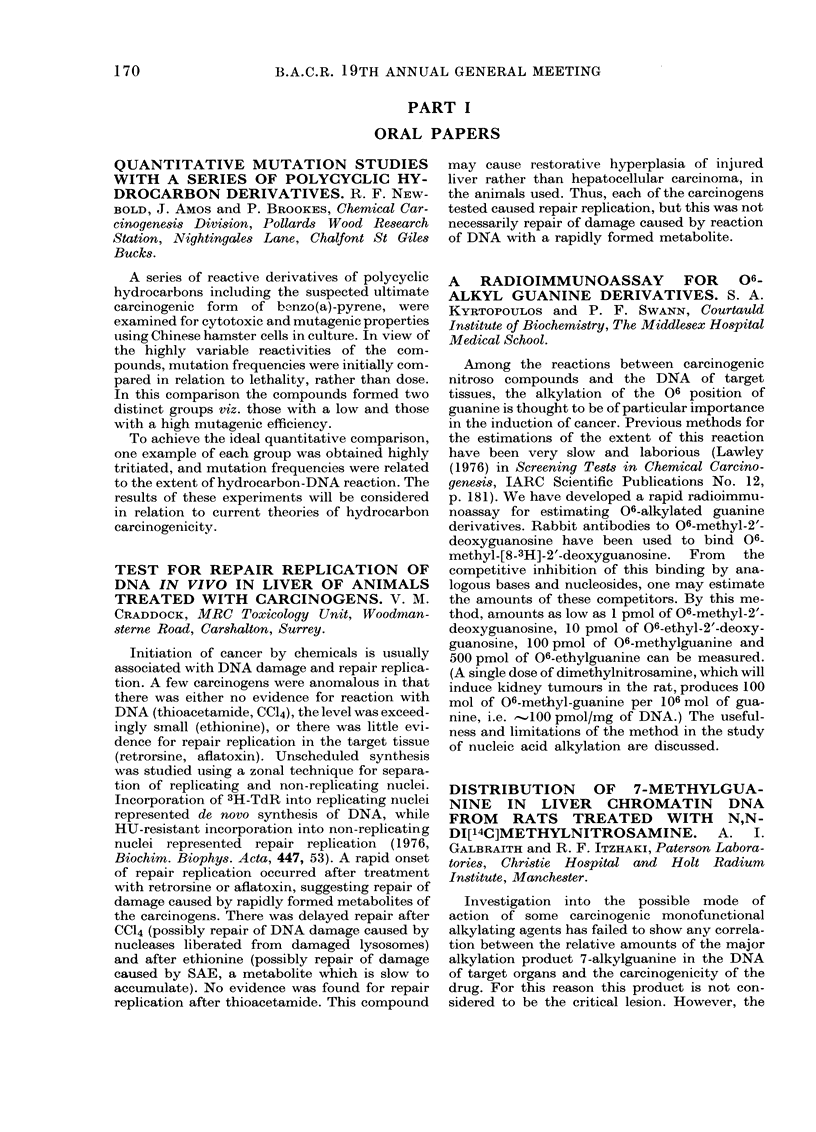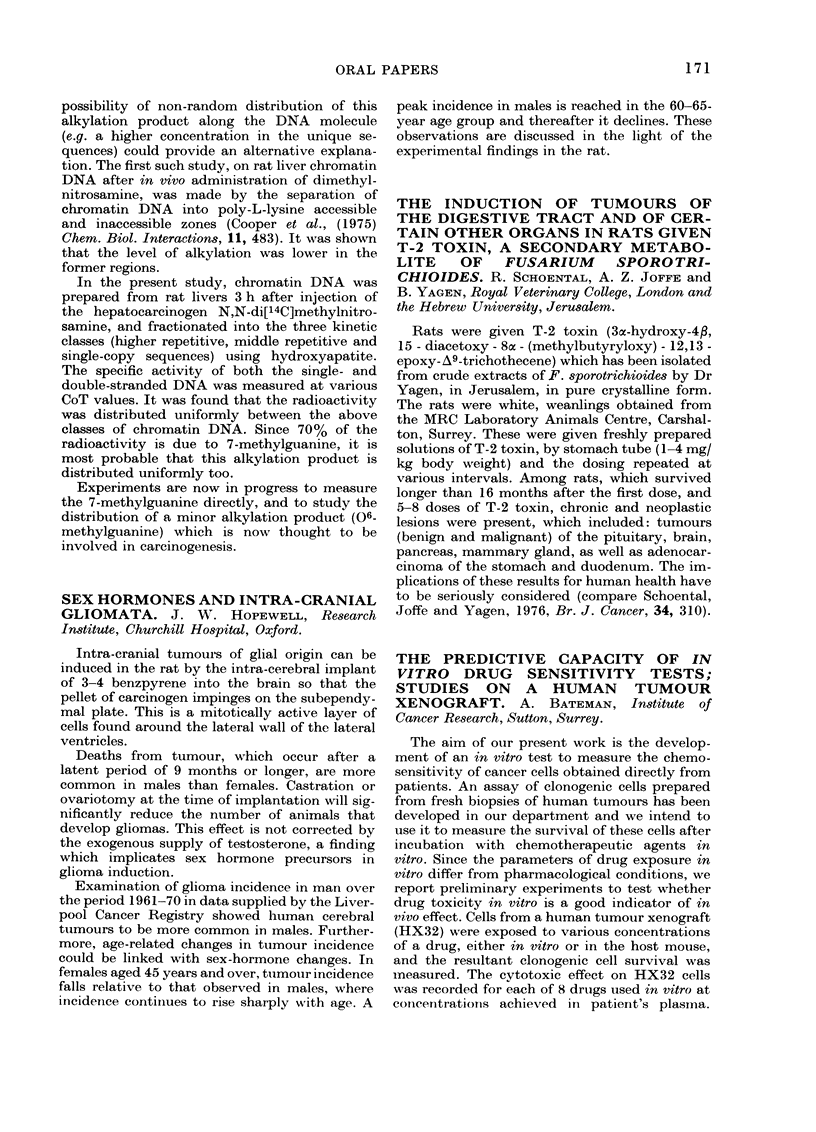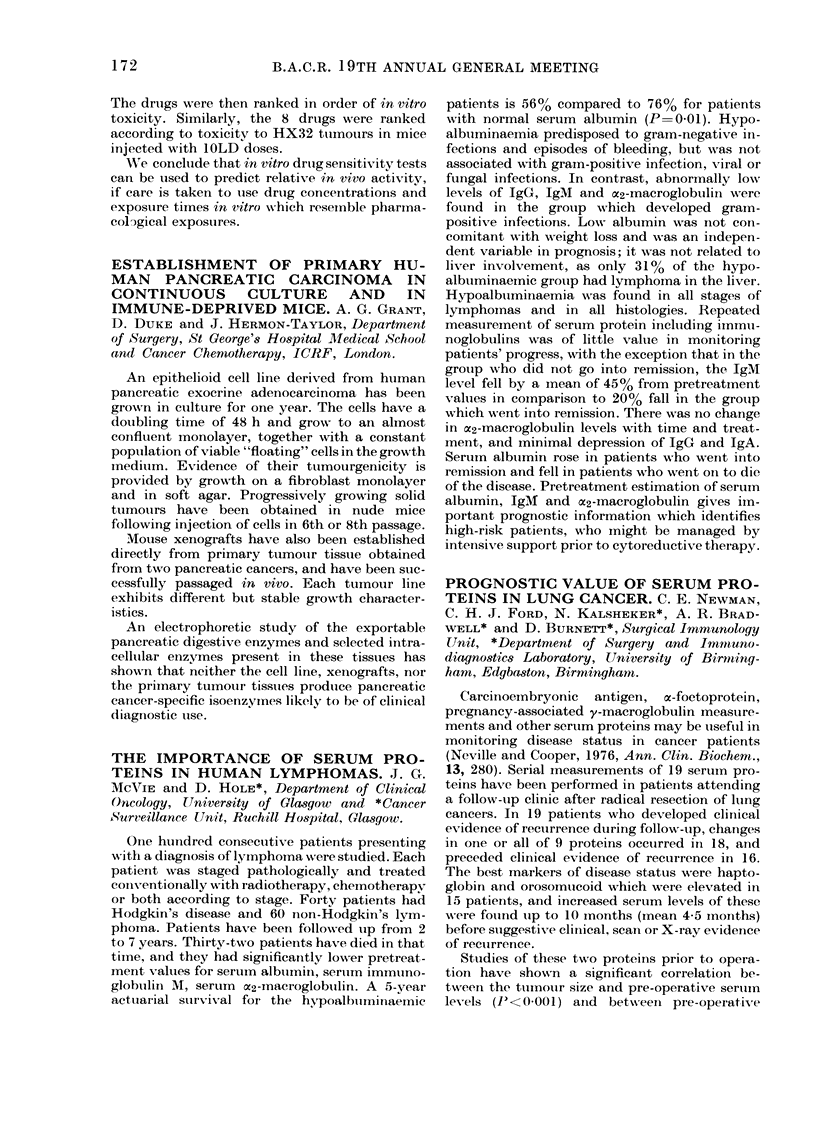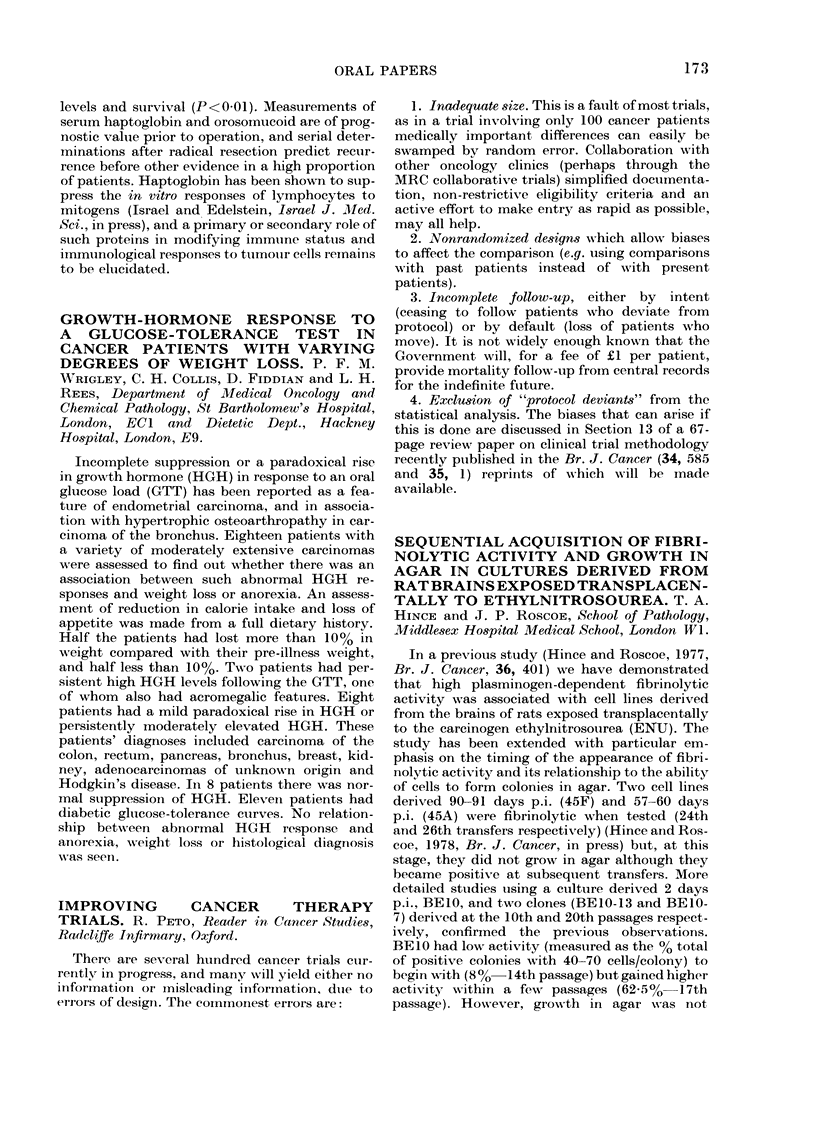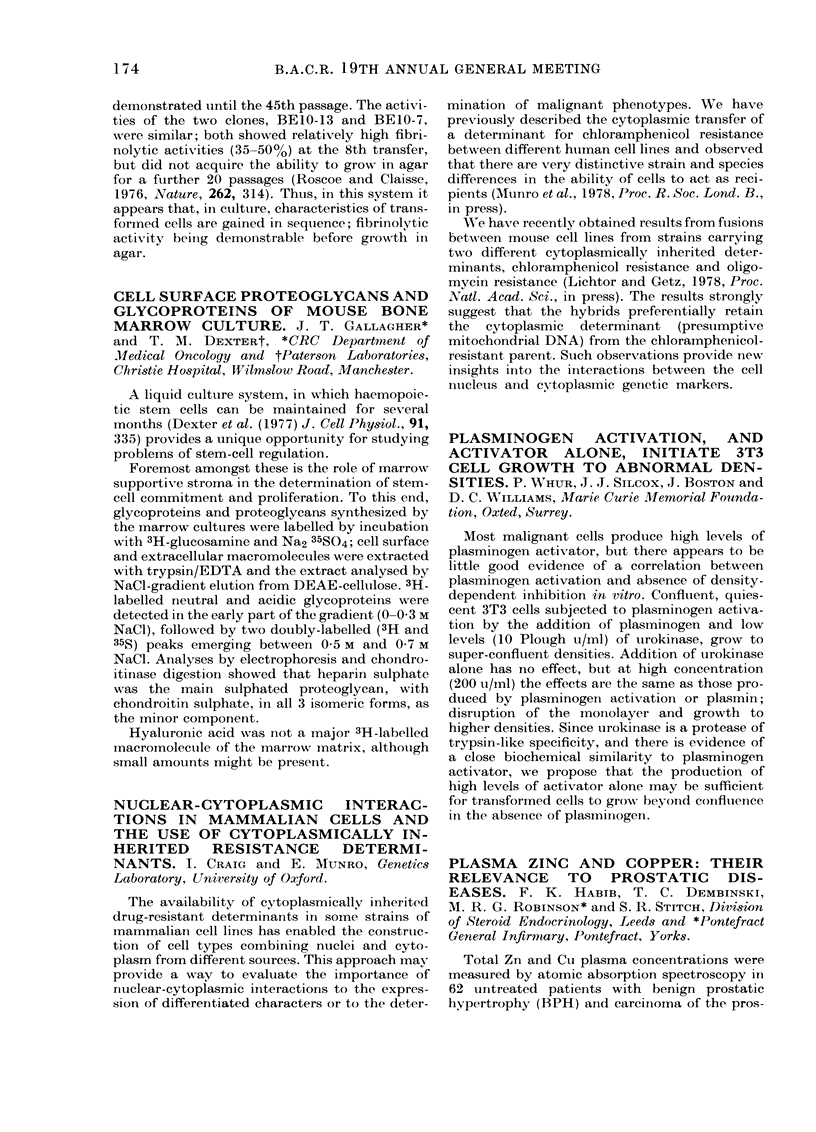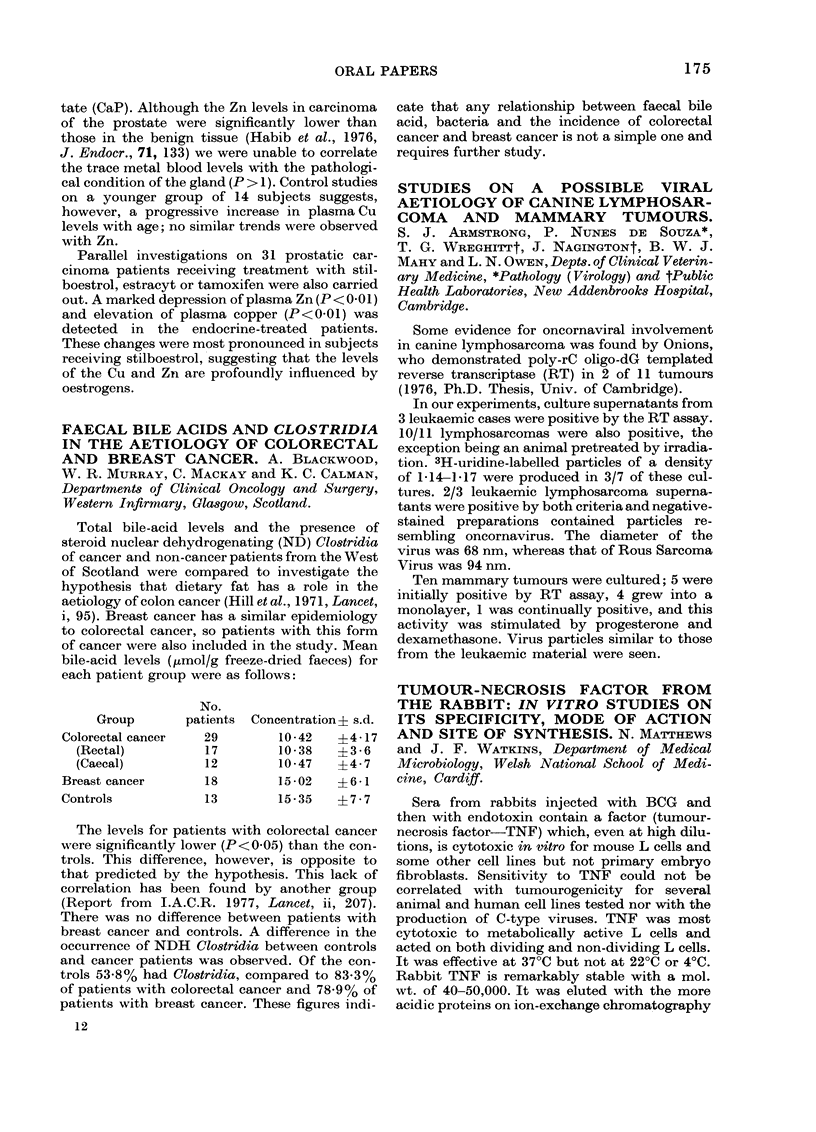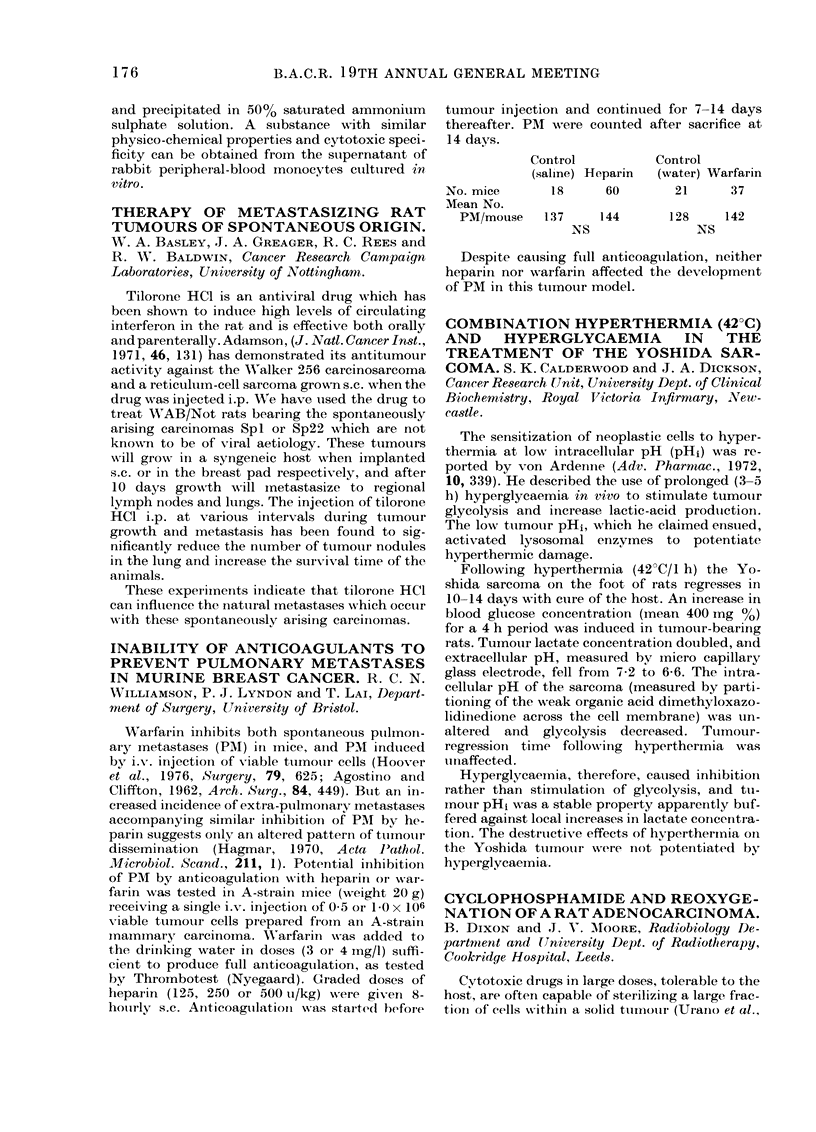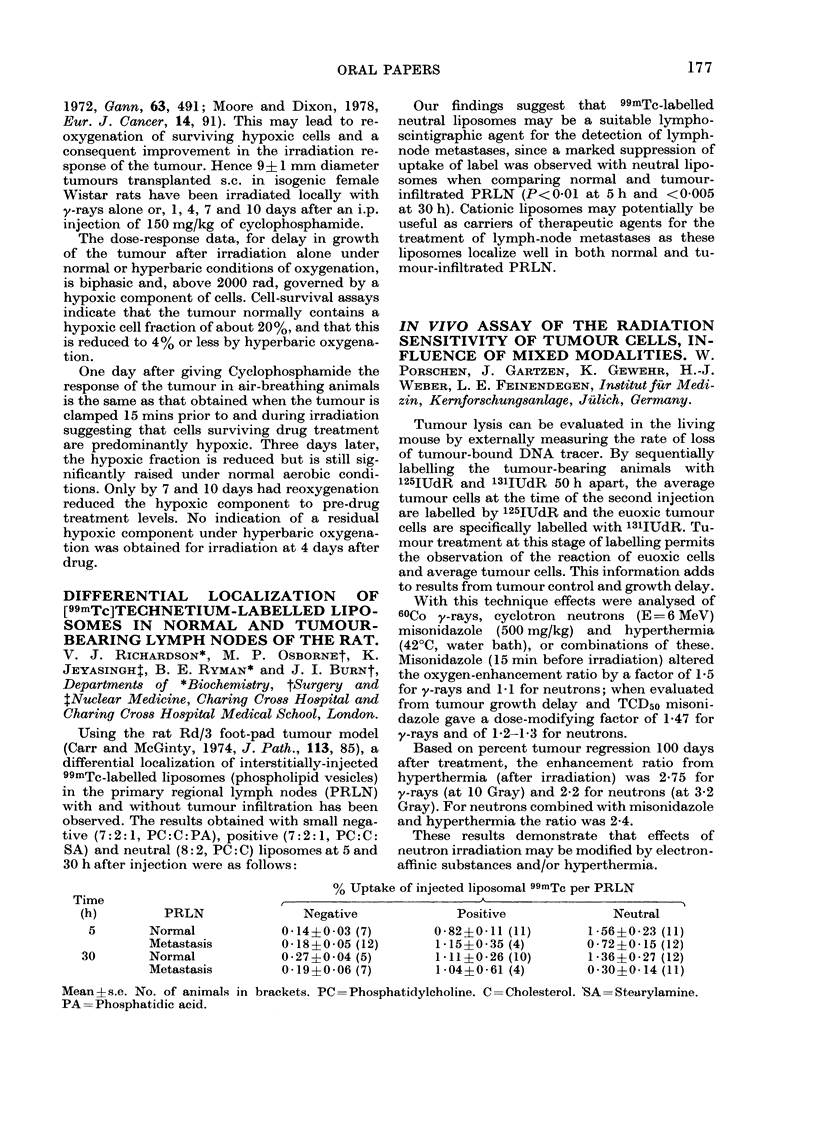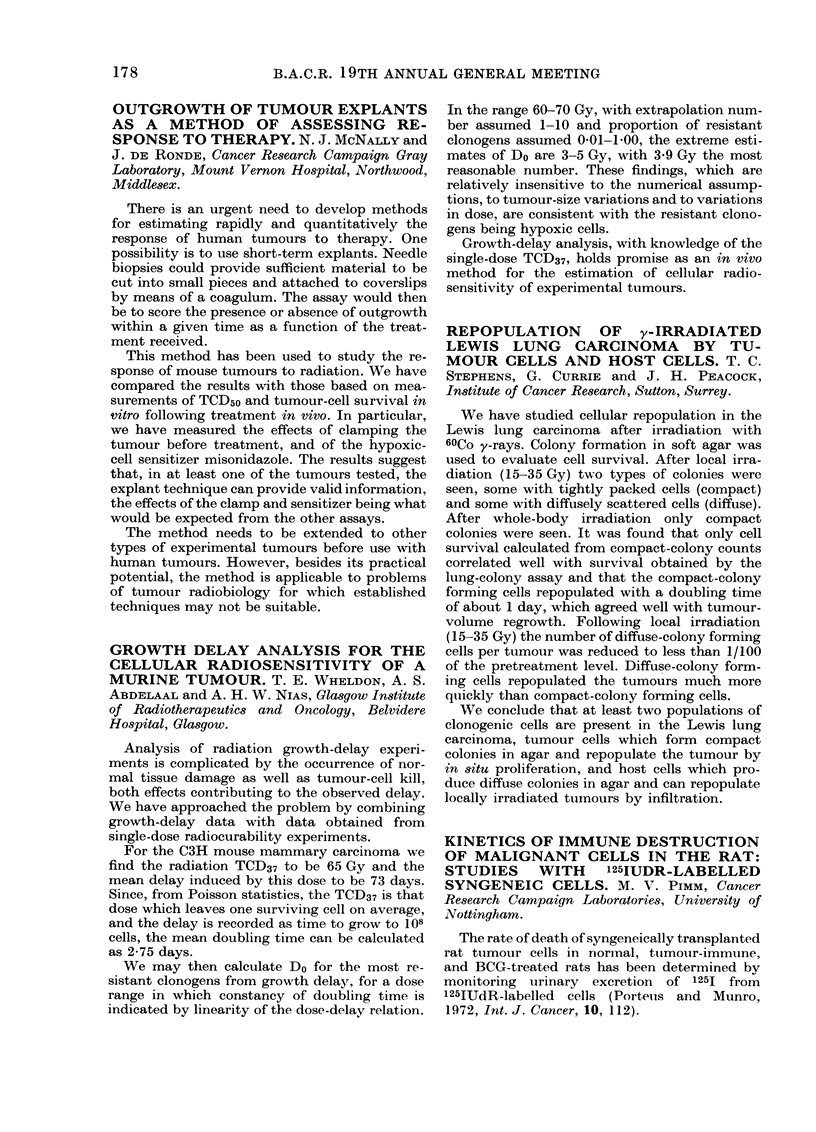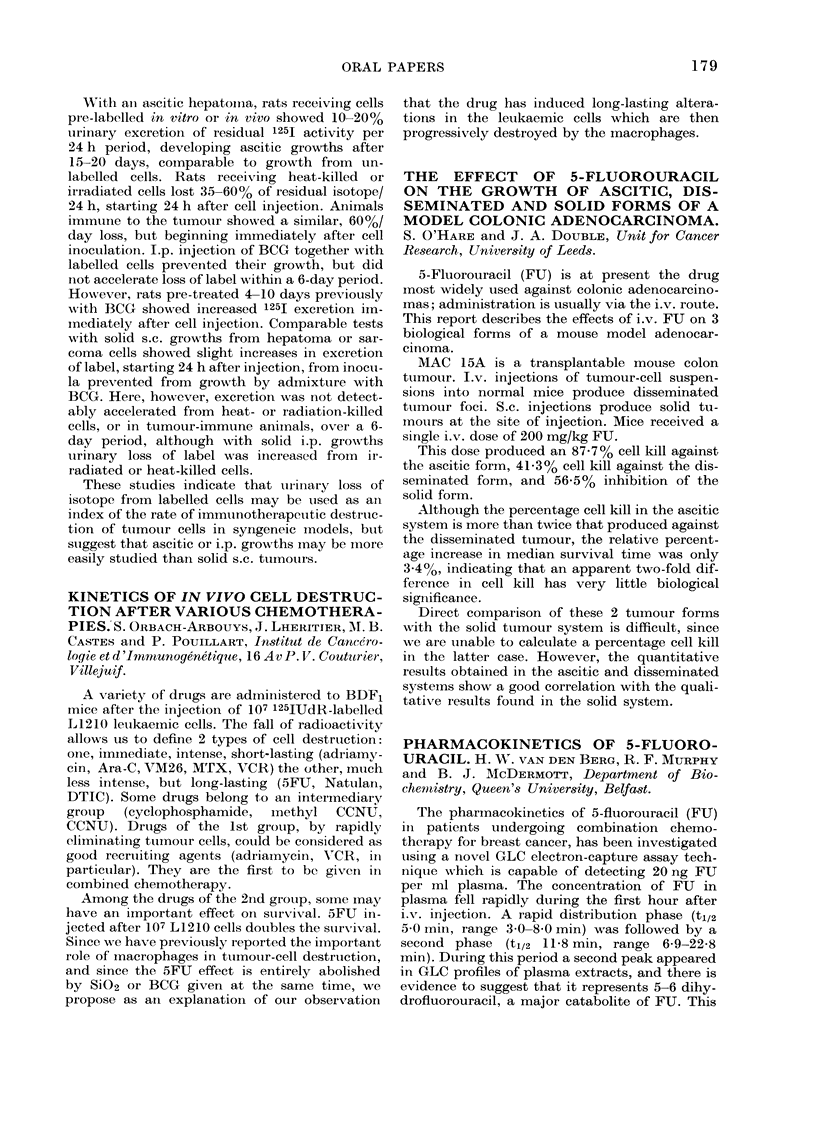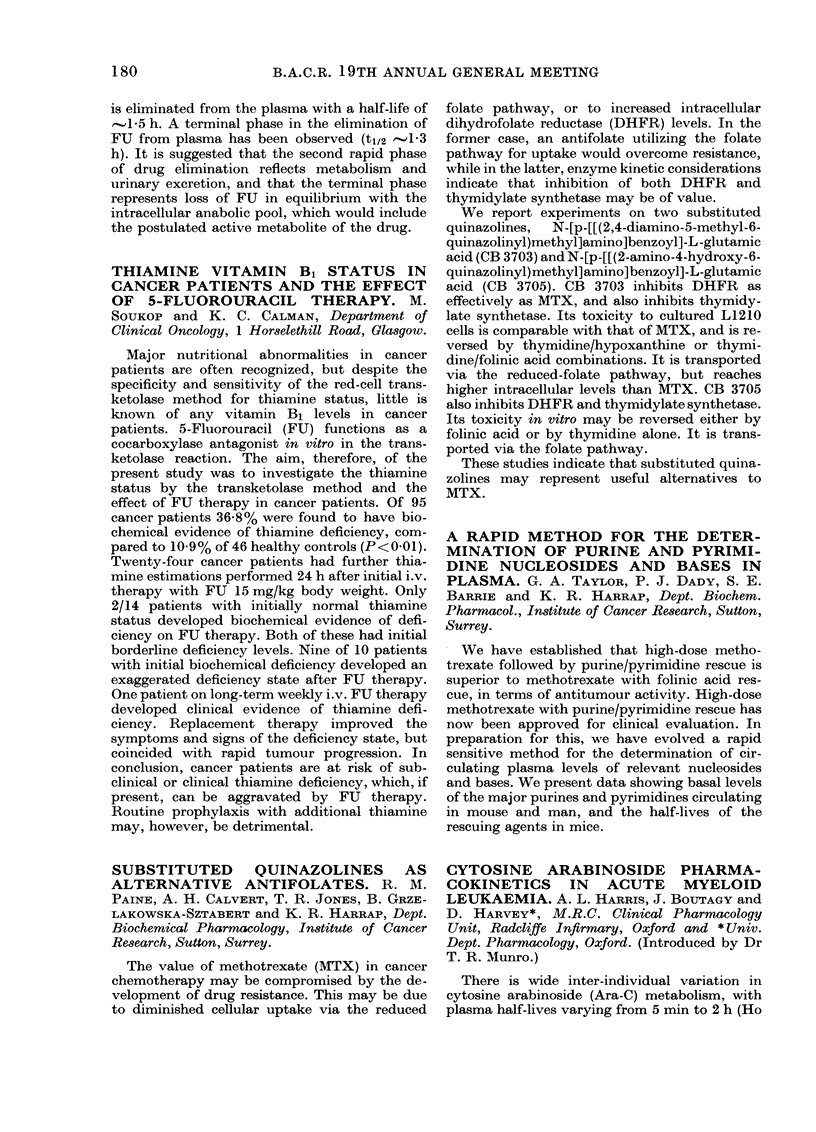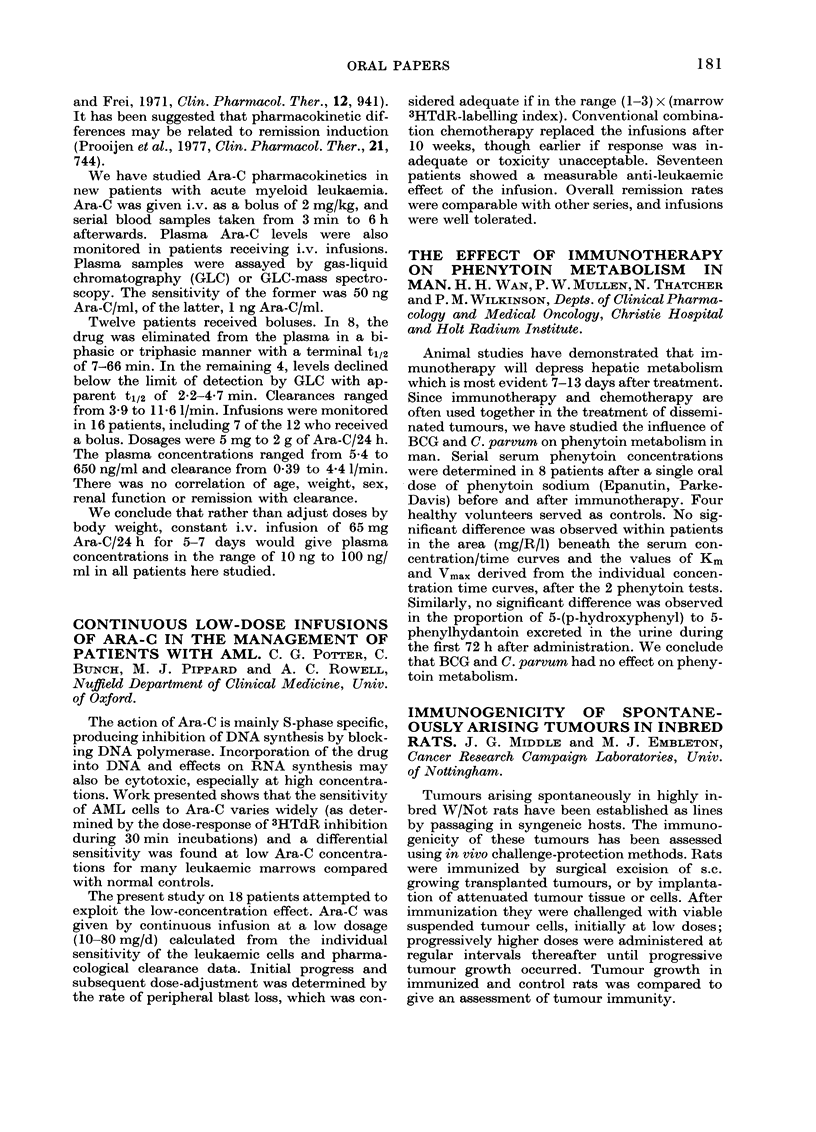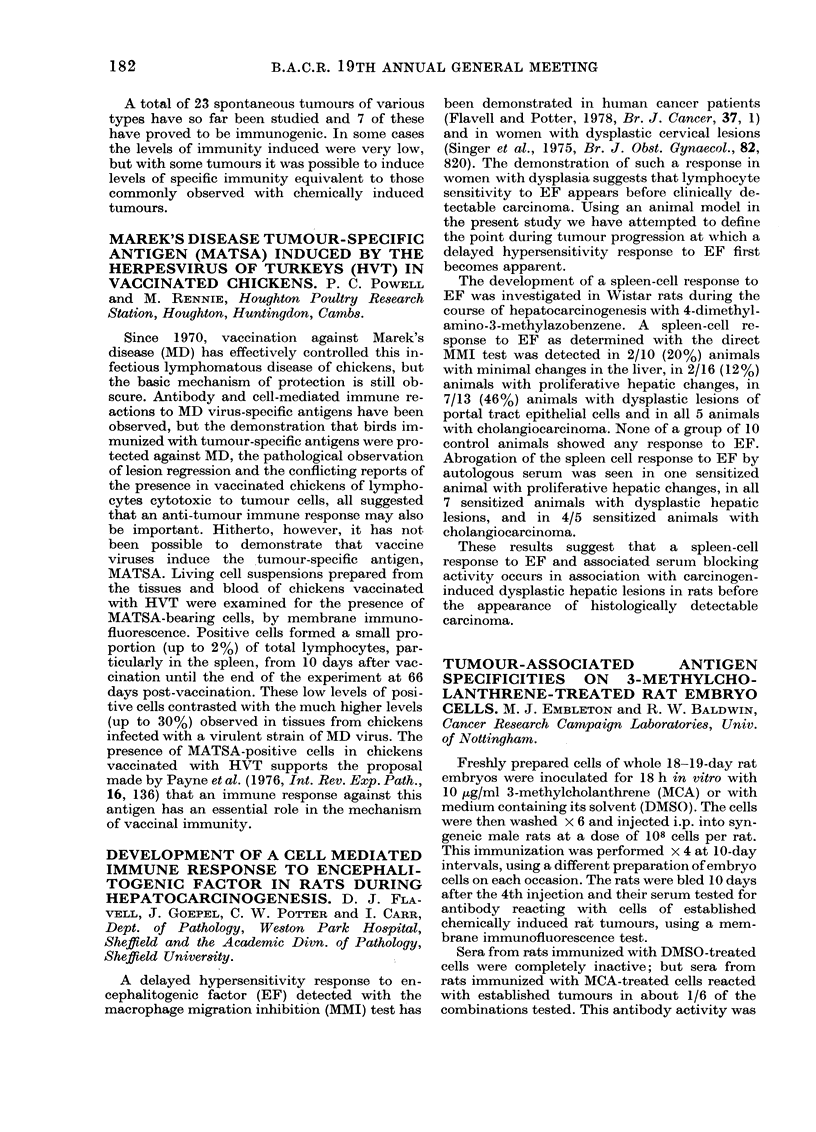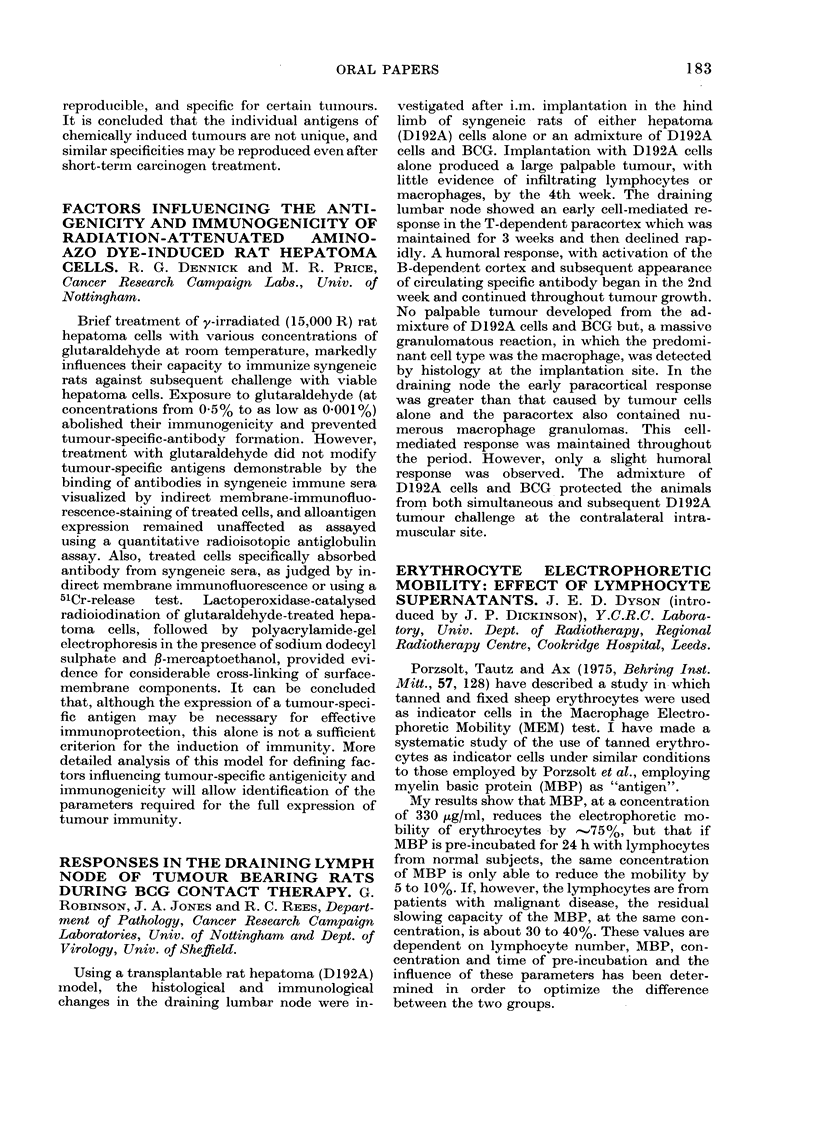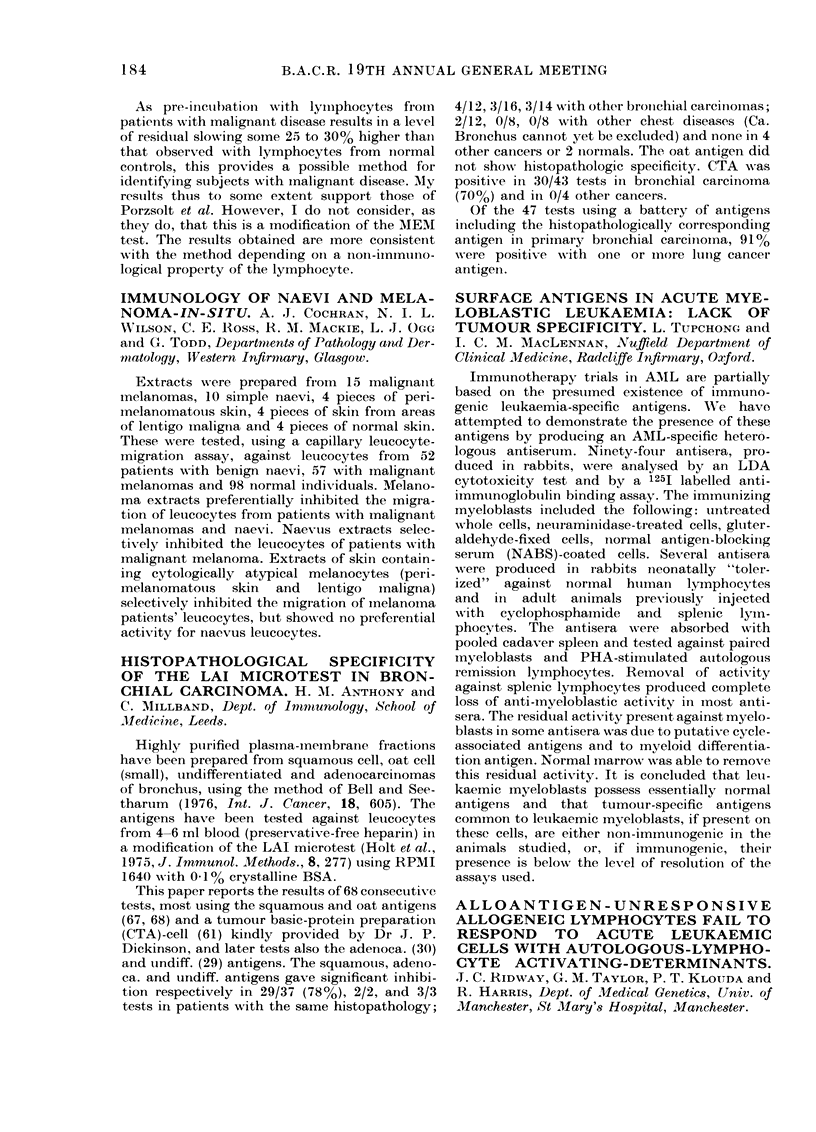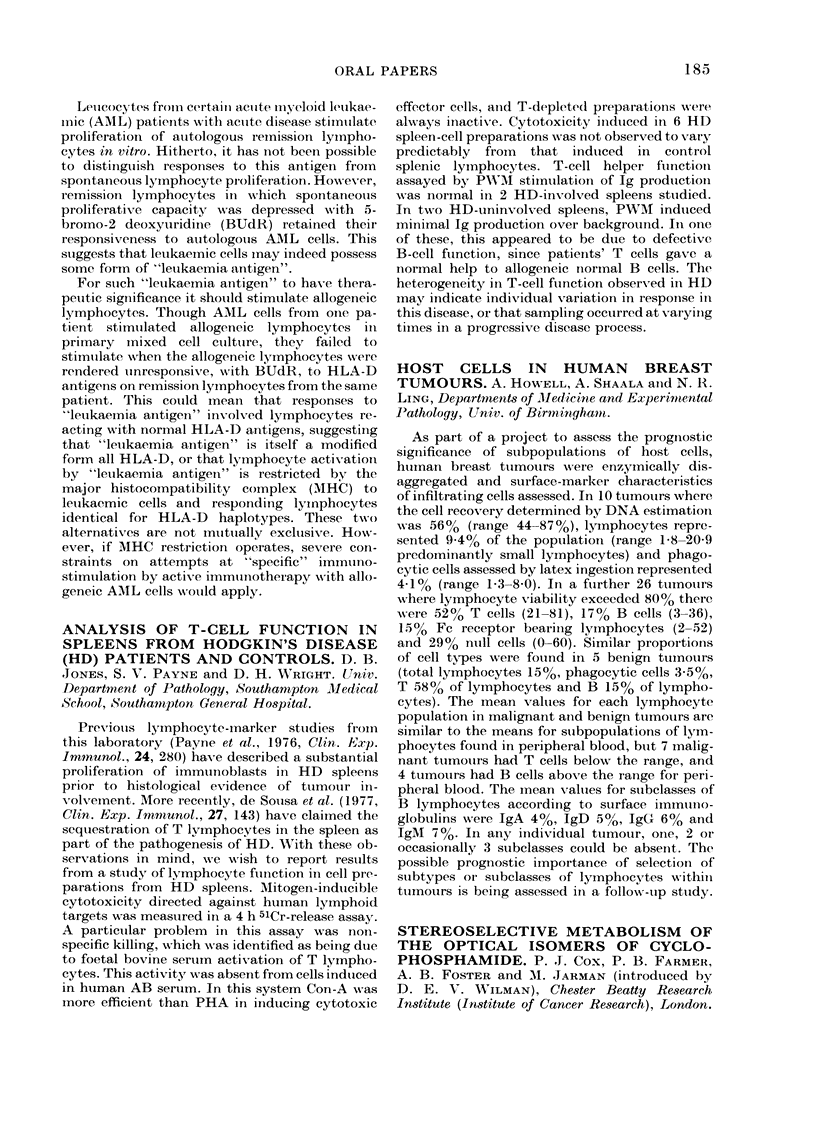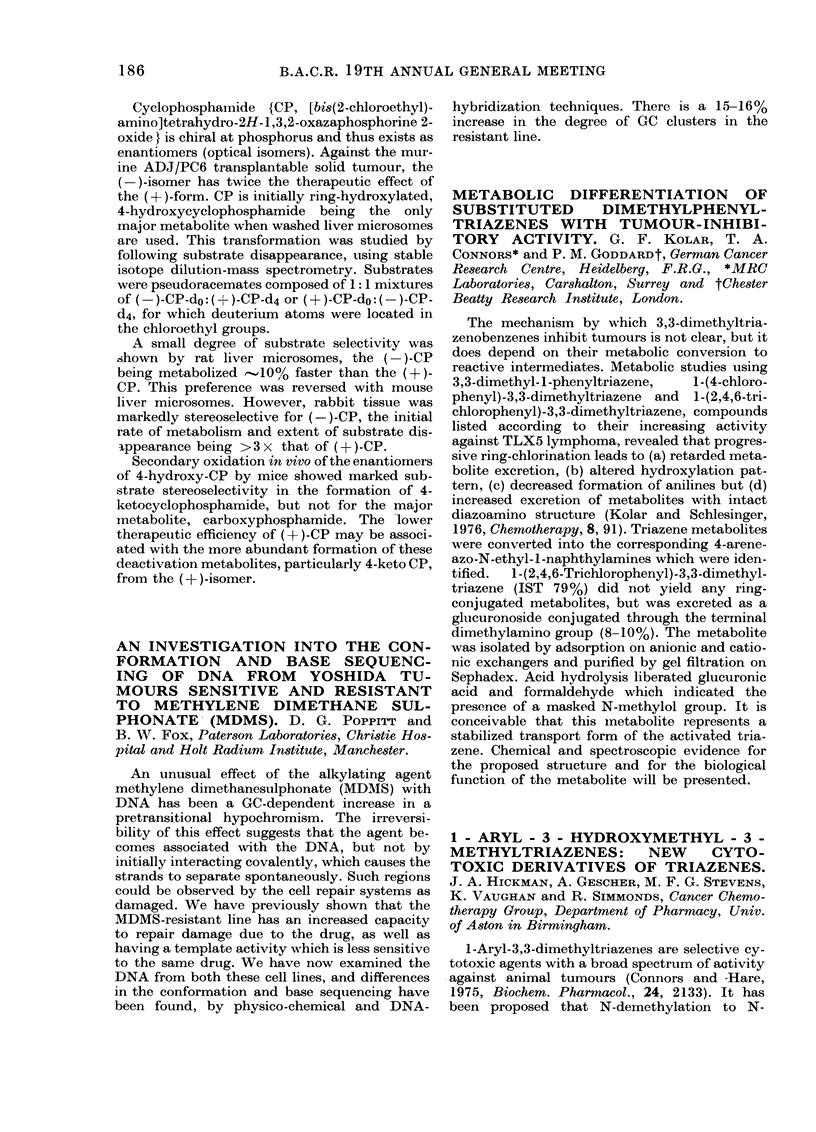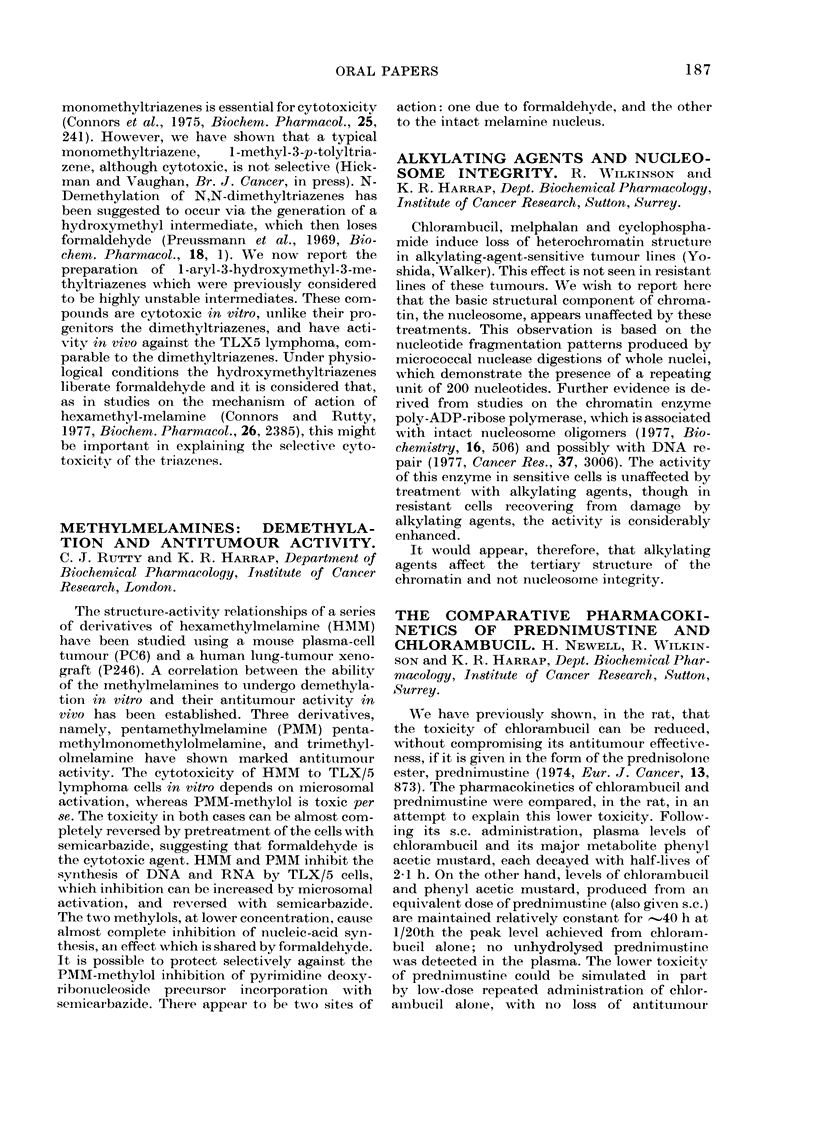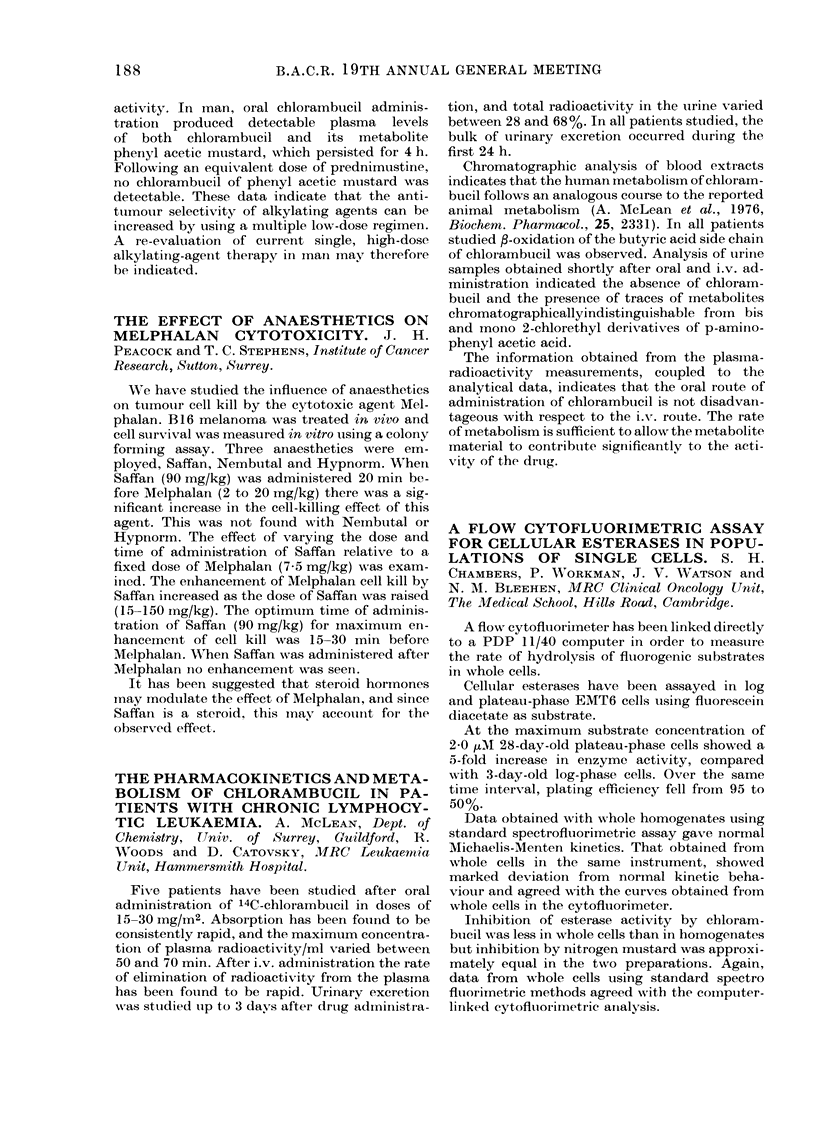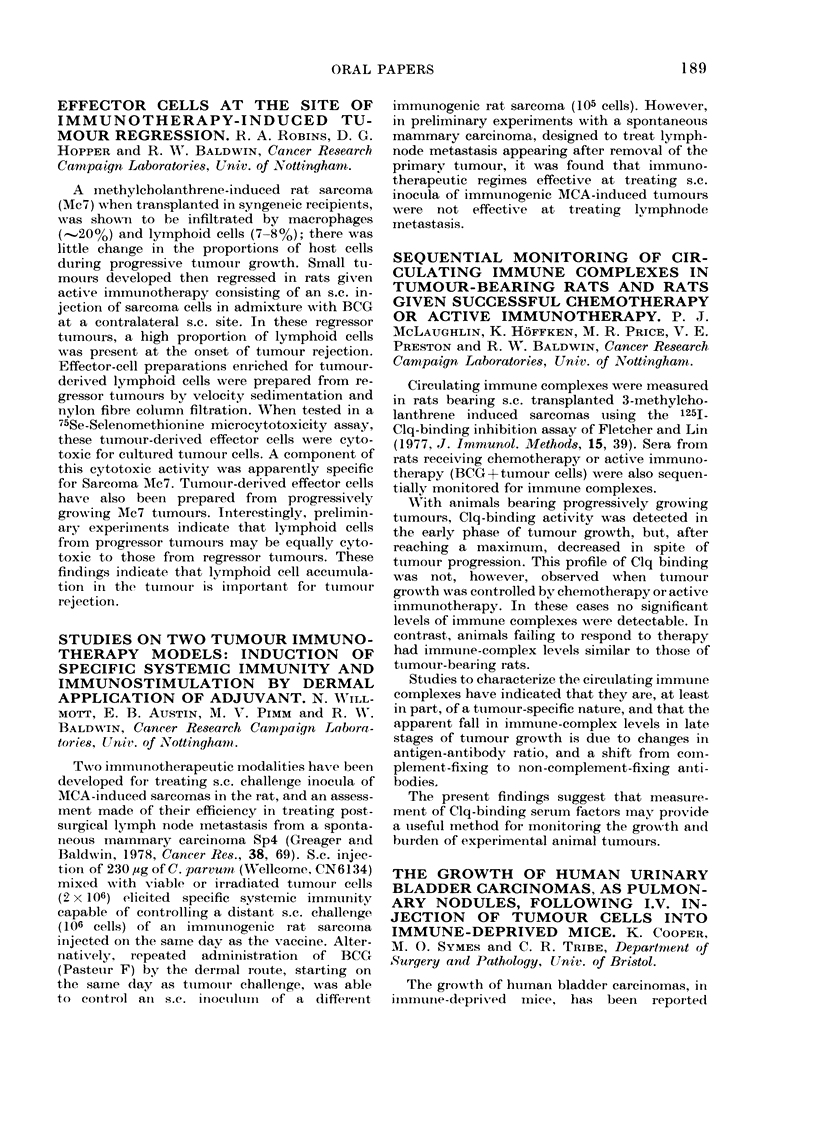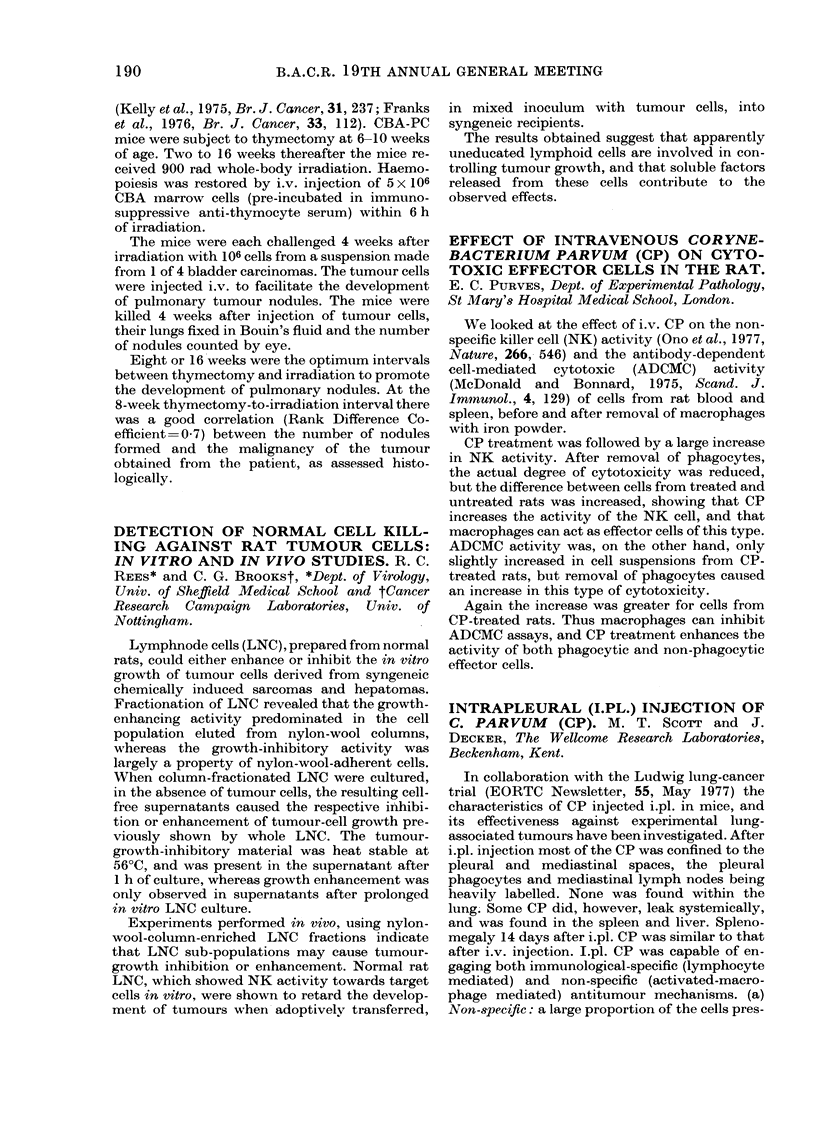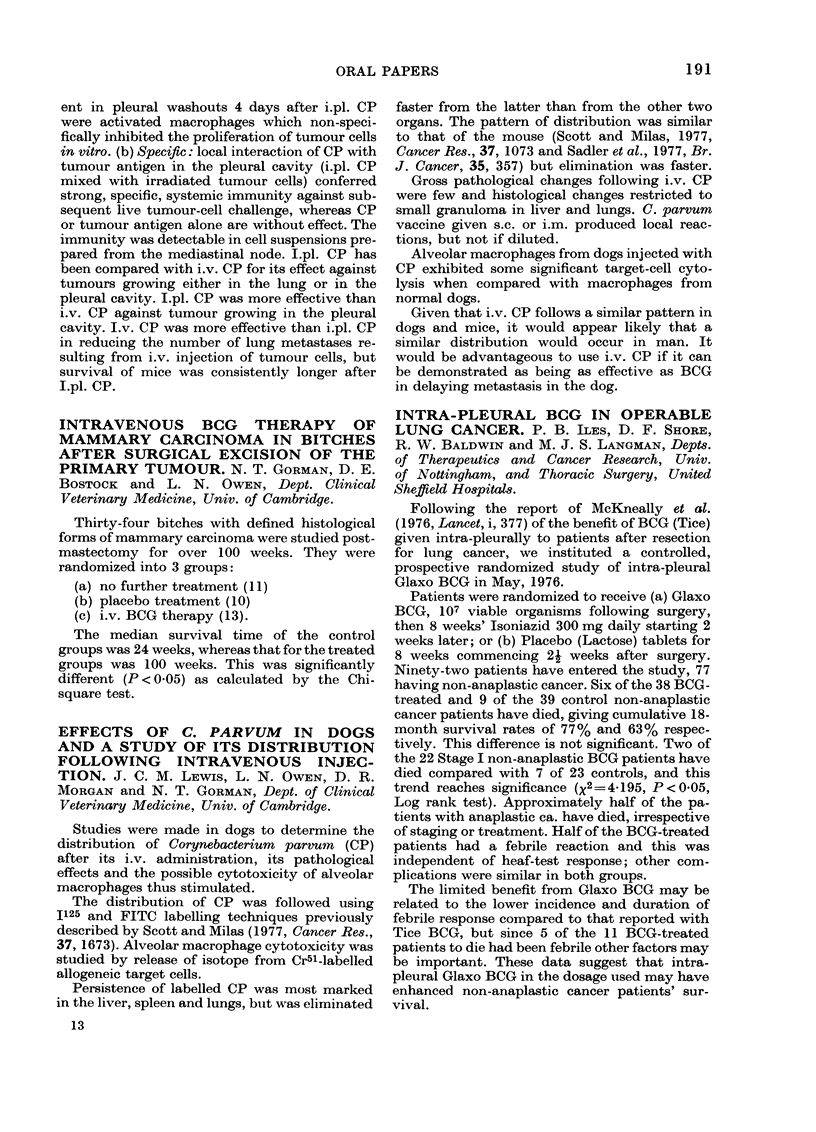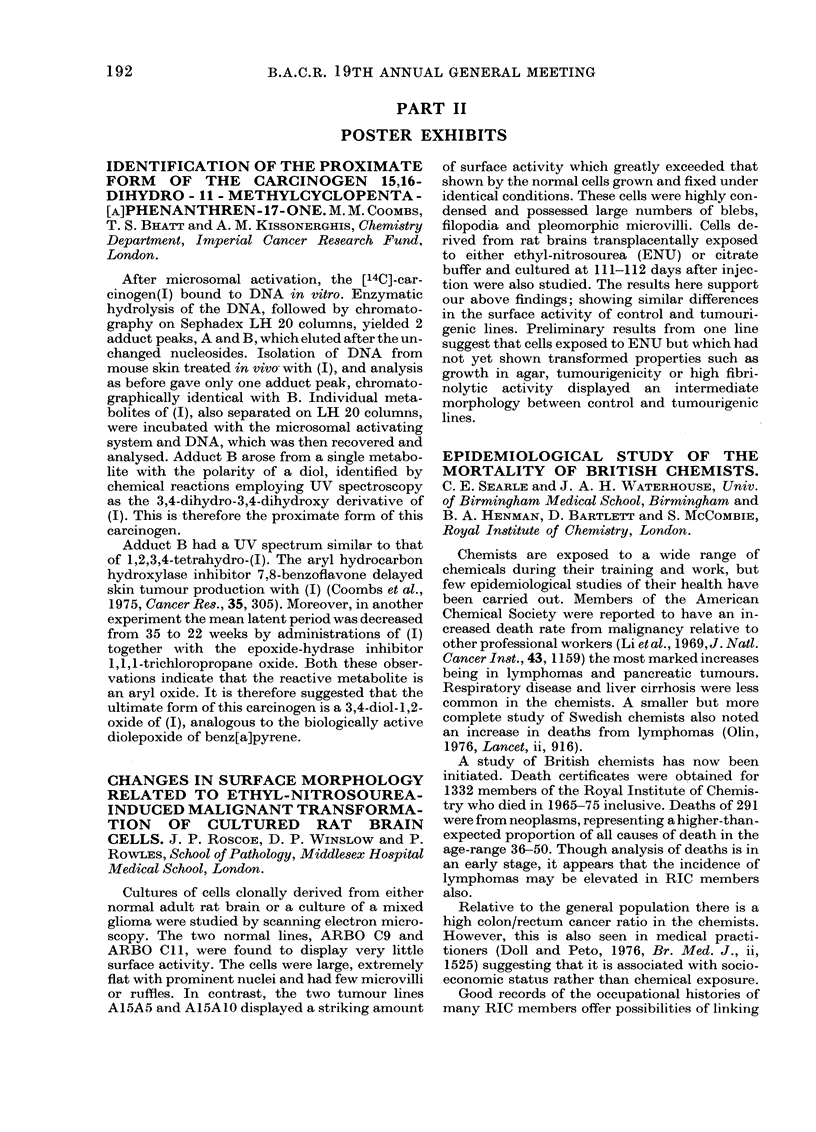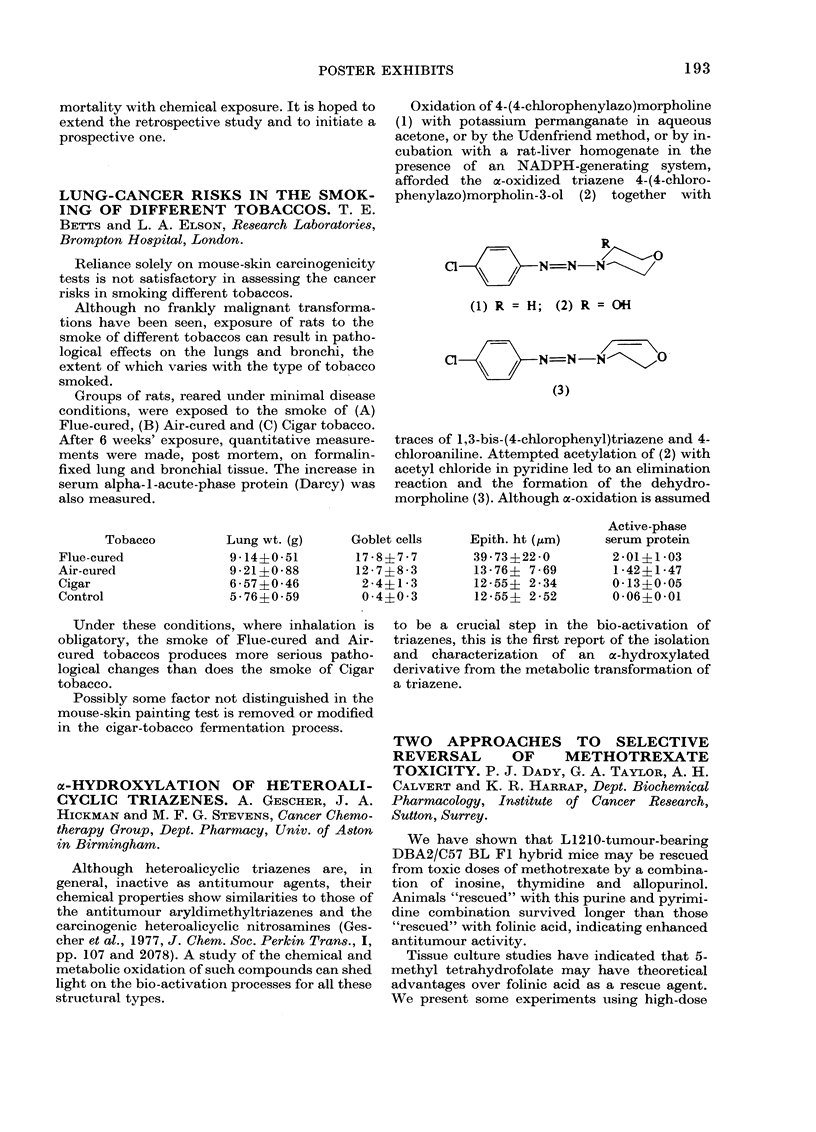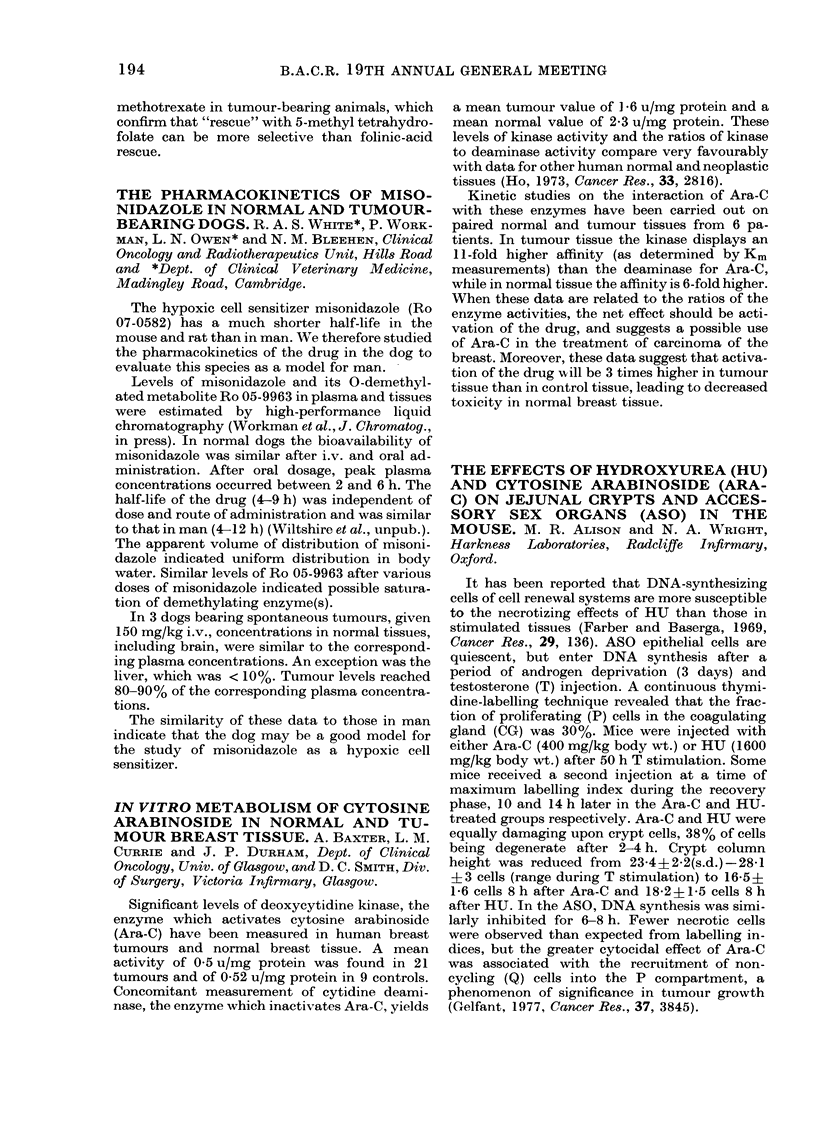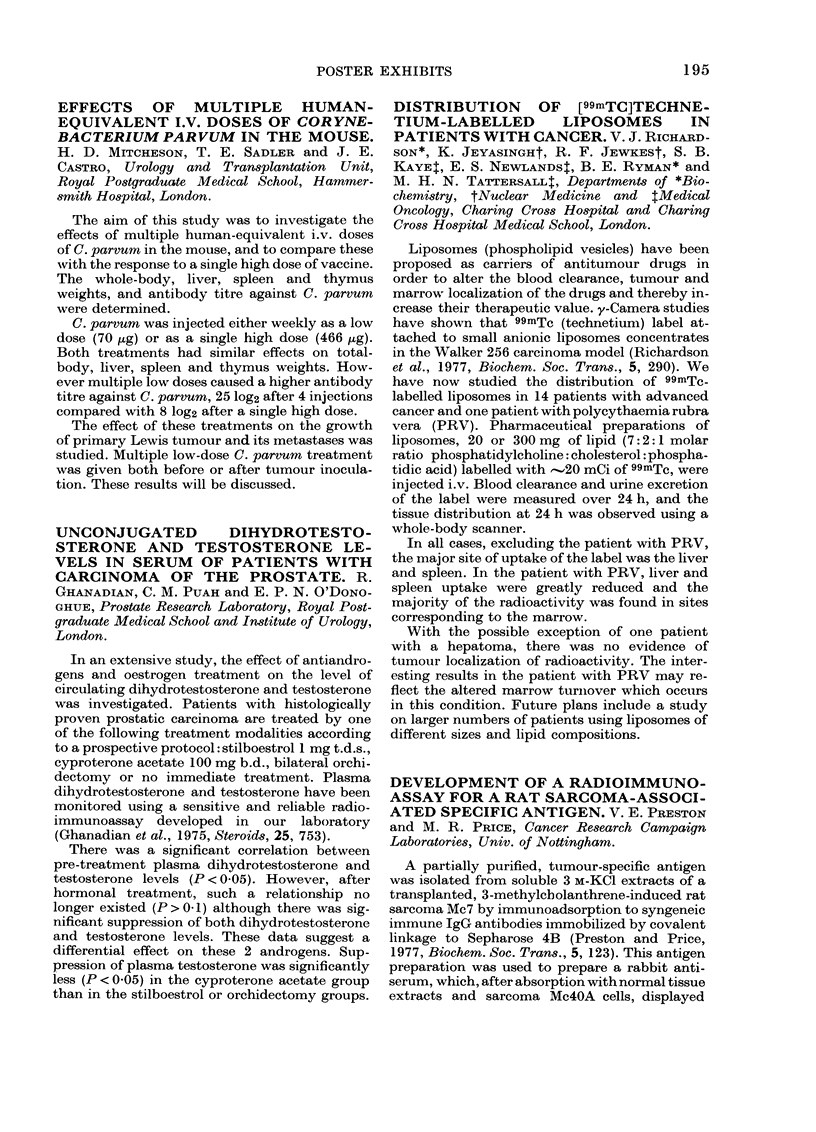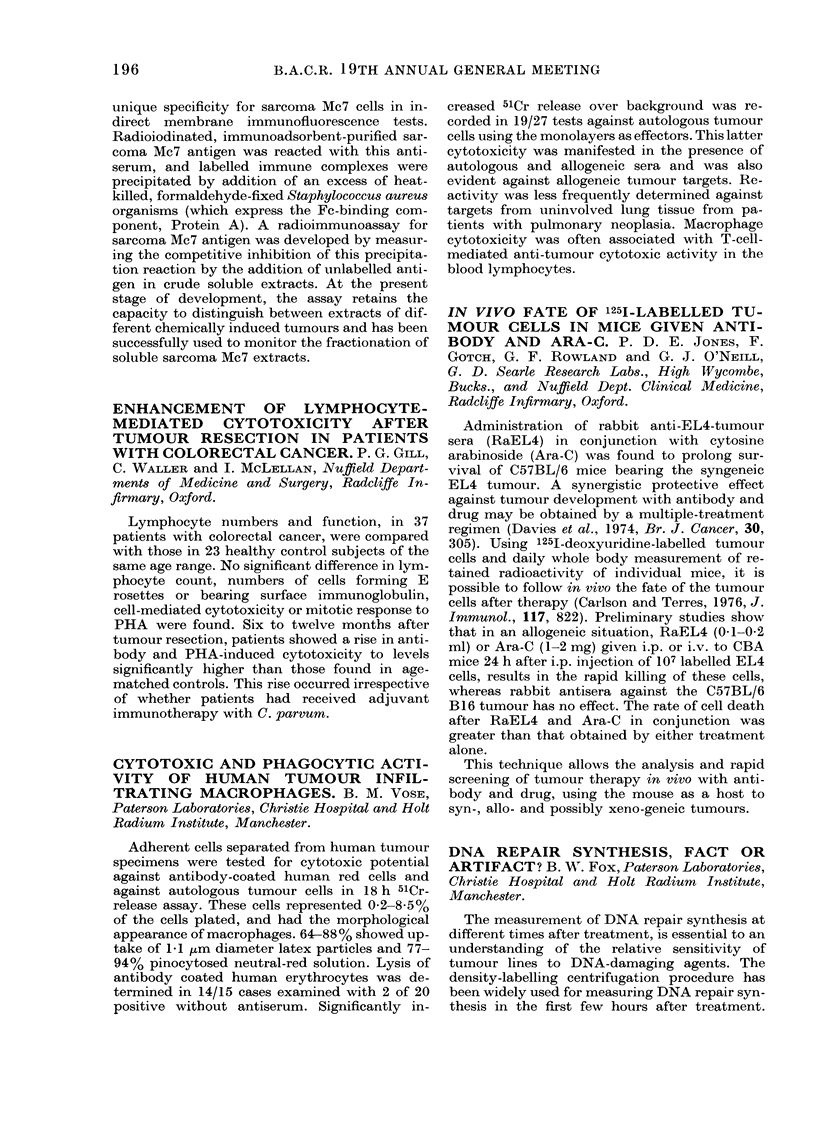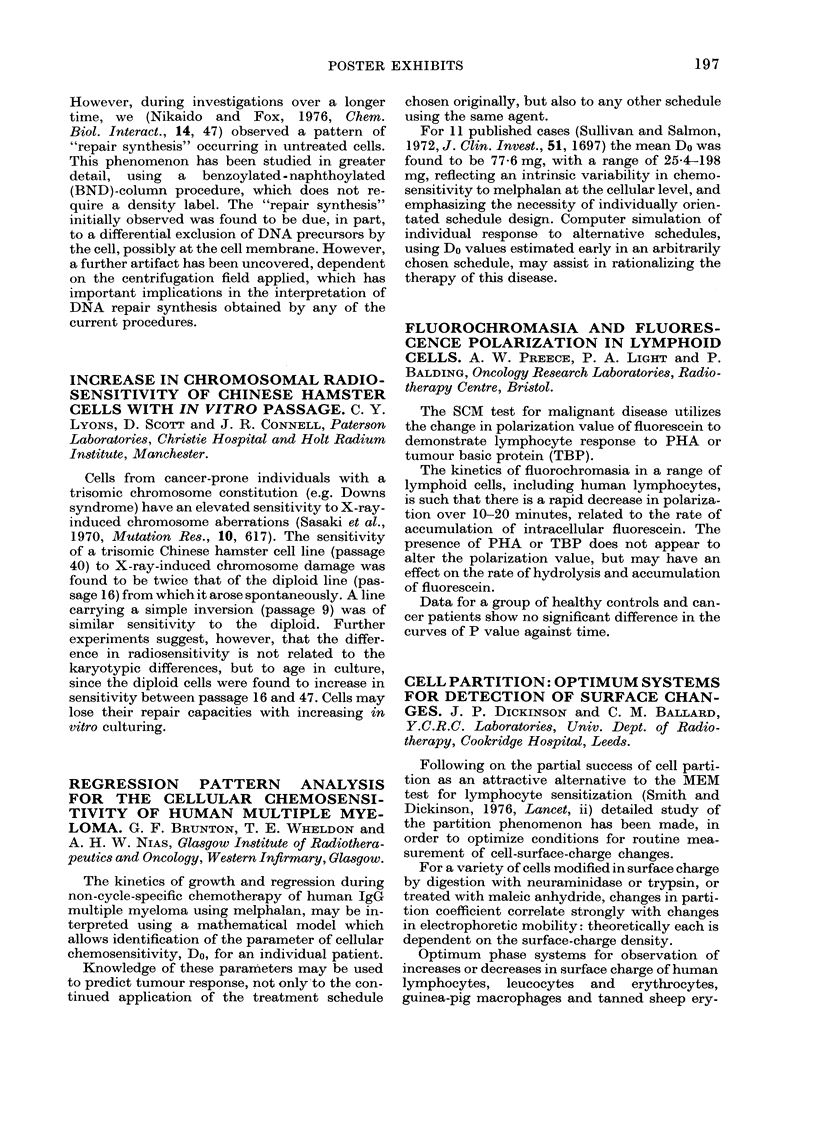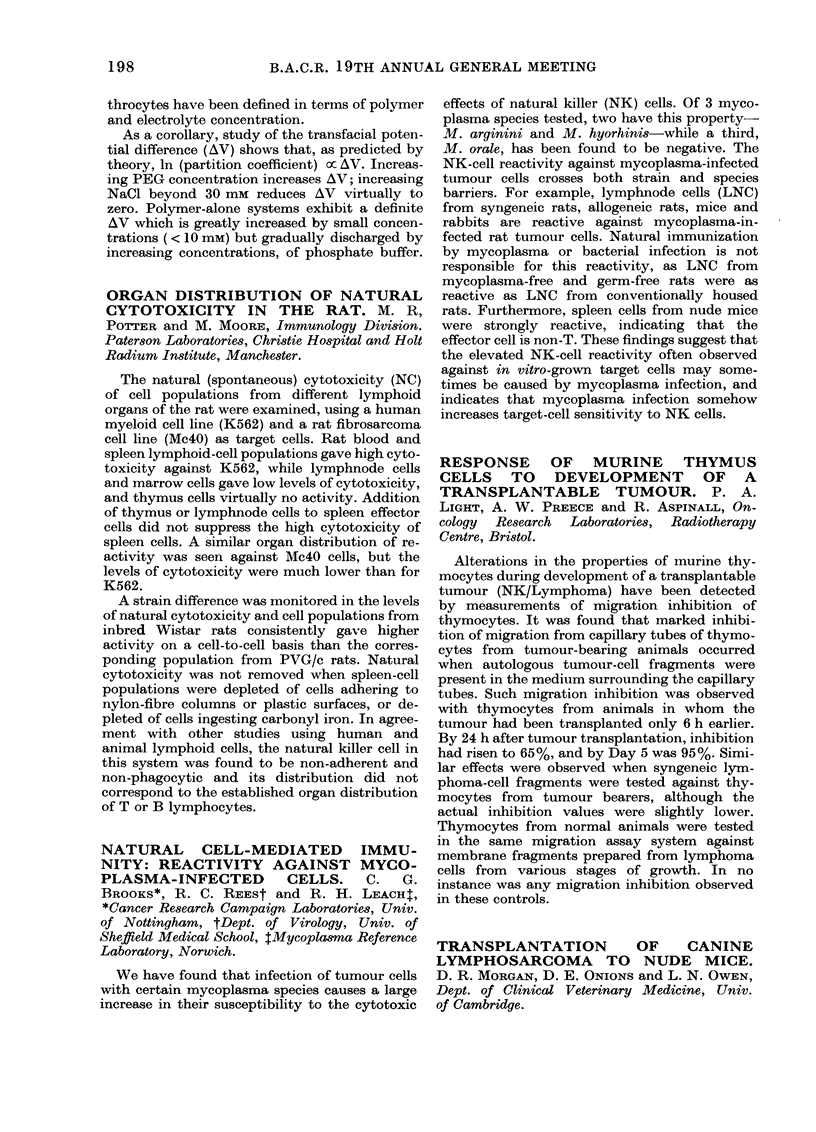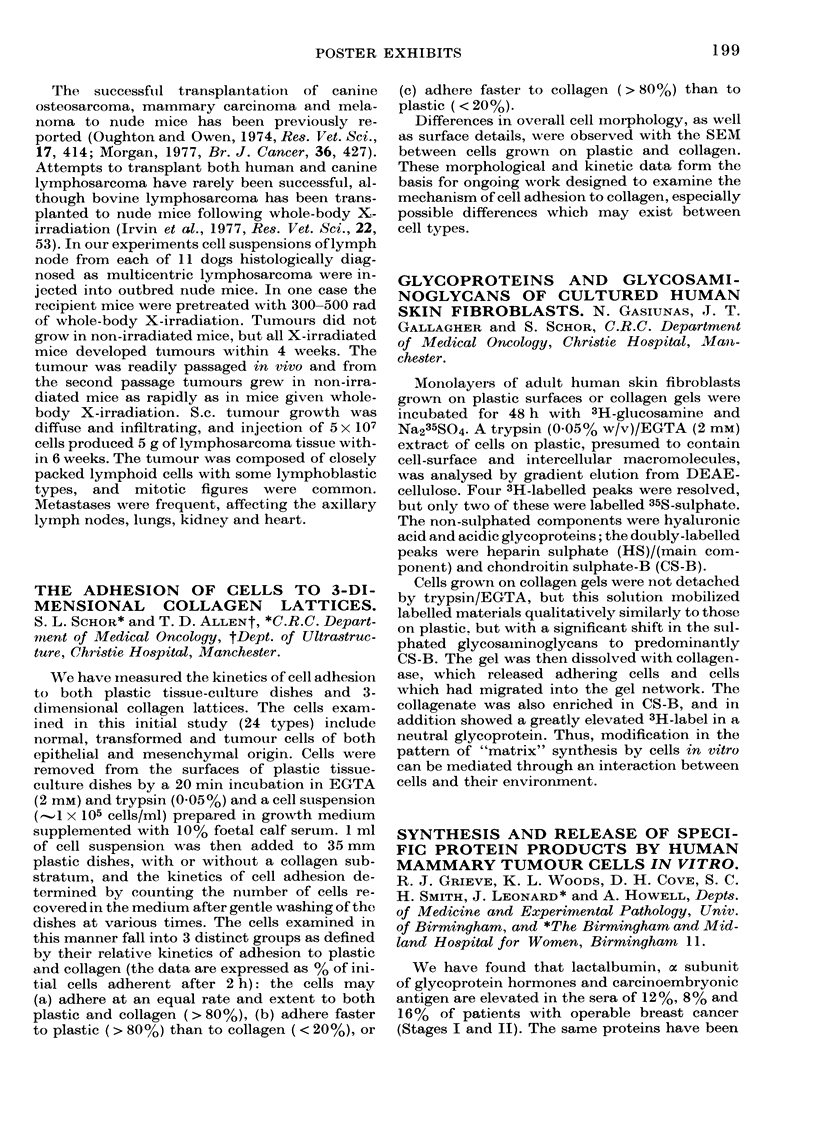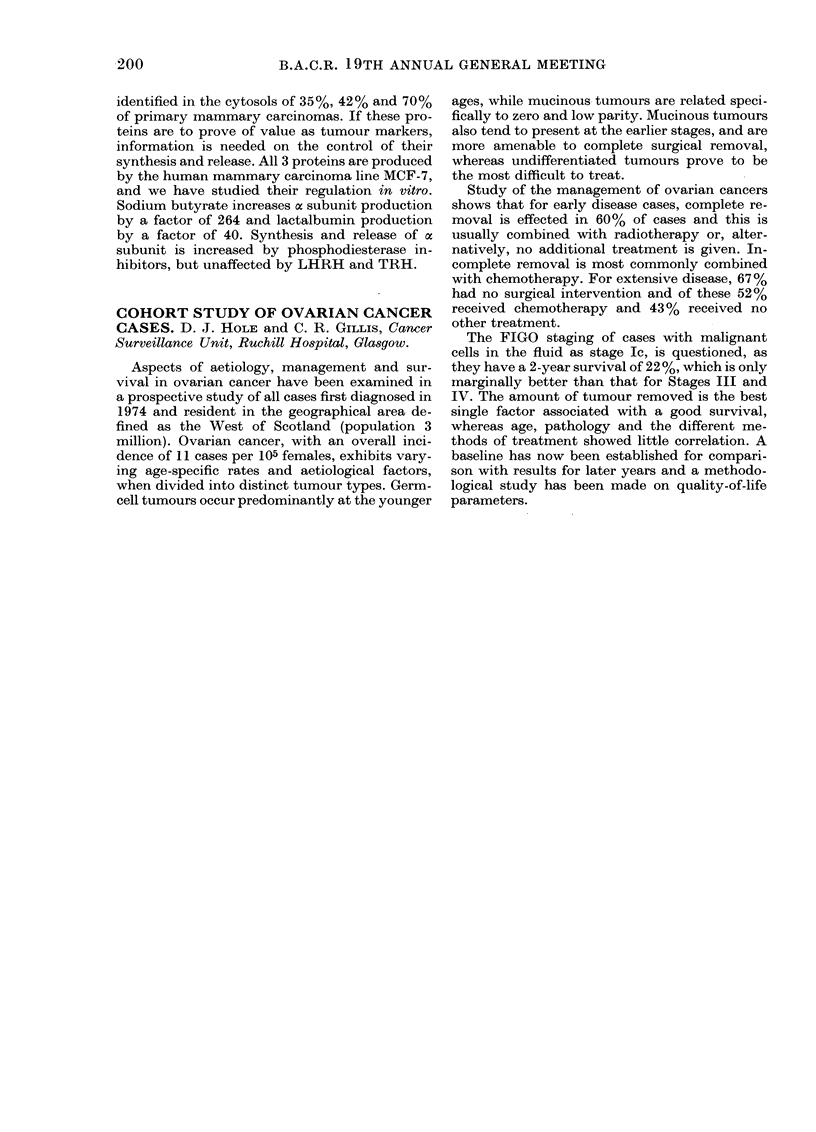# Proceedings of the British Association For Cancer Research, 19th Annual General Meeting St. Edmund Hall, University of Oxford 4--6 April, 1978.

**DOI:** 10.1038/bjc.1978.181

**Published:** 1978-07

**Authors:** 


*Br. J. Cancer* (1978) **38**, 169

### PROCEEDINGS
### of the
### BRITISH ASSOCIATION FOR CANCER RESEARCH

**19th Annual General Meeting**
**St. Edmund Hall, University of Oxford**
**4–6 April, 1978**

---

### ABSTRACTS OF MEMBERS' PROFFERED PAPERS

---

### PART I

### ORAL PAPERS

### QUANTITATIVE MUTATION STUDIES WITH A SERIES OF POLYCYCLIC HYDROCARBON DERIVATIVES.

R. F. NEWBOLD, J. AMOS and P. BROOKES, *Chemical Carcinogenesis Division, Pollards Wood Research Station, Nightingales Lane, Chalfont St Giles Bucks.*

A series of reactive derivatives of polycyclic hydrocarbons including the suspected ultimate carcinogenic form of benzo(a)-pyrene, were examined for cytotoxic and mutagenic properties using Chinese hamster cells in culture. In view of the highly variable reactivities of the compounds, mutation frequencies were initially compared in relation to lethality, rather than dose. In this comparison the compounds formed two distinct groups *viz.* those with a low and those with a high mutagenic efficiency.

To achieve the ideal quantitative comparison, one example of each group was obtained highly tritiated, and mutation frequencies were related to the extent of hydrocarbon-DNA reaction. The results of these experiments will be considered in relation to current theories of hydrocarbon carcinogenicity.

### TEST FOR REPAIR REPLICATION OF DNA *IN VIVO* IN LIVER OF ANIMALS TREATED WITH CARCINOGENS.

V. M. CRADDOCK, *MRC Toxicology Unit, Woodmansterne Road, Carshalton, Surrey.*

Initiation of cancer by chemicals is usually associated with DNA damage and repair replication. A few carcinogens were anomalous in that there was either no evidence for reaction with DNA (thioacetamide, $CCl_4$), the level was exceedingly small (ethionine), or there was little evidence for repair replication in the target tissue (retrorsine, aflatoxin). Unscheduled synthesis was studied using a zonal technique for separation of replicating and non-replicating nuclei. Incorporation of $^3$H-TdR into replicating nuclei represented *de novo* synthesis of DNA, while HU-resistant incorporation into non-replicating nuclei represented repair replication (1976, *Biochim. Biophys. Acta*, **447,** 53). A rapid onset of repair replication occurred after treatment with retrorsine or aflatoxin, suggesting repair of damage caused by rapidly formed metabolites of the carcinogens. There was delayed repair after $CCl_4$ (possibly repair of DNA damage caused by nucleases liberated from damaged lysosomes) and after ethionine (possibly repair of damage caused by SAE, a metabolite which is slow to accumulate). No evidence was found for repair replication after thioacetamide. This compound may cause restorative hyperplasia of injured liver rather than hepatocellular carcinoma, in the animals used. Thus, each of the carcinogens tested caused repair replication, but this was not necessarily repair of damage caused by reaction of DNA with a rapidly formed metabolite.

### A RADIOIMMUNOASSAY FOR O$^6$-ALKYL GUANINE DERIVATIVES.

S. A. KYRTOPOULOS and P. F. SWANN, *Courtauld Institute of Biochemistry, The Middlesex Hospital Medical School.*

Among the reactions between carcinogenic nitroso compounds and the DNA of target tissues, the alkylation of the O$^6$ position of guanine is thought to be of particular importance in the induction of cancer. Previous methods for the estimations of the extent of this reaction have been very slow and laborious (Lawley (1976) in *Screening Tests in Chemical Carcinogenesis*, IARC Scientific Publications No. 12, p. 181). We have developed a rapid radioimmunoassay for estimating O$^6$-alkylated guanine derivatives. Rabbit antibodies to O$^6$-methyl-2′-deoxyguanosine have been used to bind O$^6$-methyl-[8-$^3$H]-2′-deoxyguanosine. From the competitive inhibition of this binding by analogous bases and nucleosides, one may estimate the amounts of these competitors. By this method, amounts as low as 1 pmol of O$^6$-methyl-2′-deoxyguanosine, 10 pmol of O$^6$-ethyl-2′-deoxyguanosine, 100 pmol of O$^6$-methylguanine and 500 pmol of O$^6$-ethylguanine can be measured. (A single dose of dimethylnitrosamine, which will induce kidney tumours in the rat, produces 100 mol of O$^6$-methyl-guanine per $10^6$ mol of guanine, i.e. $\sim$100 pmol/mg of DNA.) The usefulness and limitations of the method in the study of nucleic acid alkylation are discussed.

### DISTRIBUTION OF 7-METHYLGUANINE IN LIVER CHROMATIN DNA FROM RATS TREATED WITH N,N-DI[$^{14}$C]METHYLNITROSAMINE.

A. I. GALBRAITH and R. F. ITZHAKI, *Paterson Laboratories, Christie Hospital and Holt Radium Institute, Manchester.*

Investigation into the possible mode of action of some carcinogenic monofunctional alkylating agents has failed to show any correlation between the relative amounts of the major alkylation product 7-alkylguanine in the DNA of target organs and the carcinogenicity of the drug. For this reason this product is not considered to be the critical lesion. However, the possibility of non-random distribution of this alkylation product along the DNA molecule (*e.g.* a higher concentration in the unique sequences) could provide an alternative explanation. The first such study, on rat liver chromatin DNA after *in vivo* administration of dimethylnitrosamine, was made by the separation of chromatin DNA into poly-L-lysine accessible and inaccessible zones (Cooper *et al.*, (1975) *Chem. Biol. Interactions*, **11,** 483). It was shown that the level of alkylation was lower in the former regions.

In the present study, chromatin DNA was prepared from rat livers 3 h after injection of the hepatocarcinogen N,N-di[$^{14}$C]methylnitrosamine, and fractionated into the three kinetic classes (higher repetitive, middle repetitive and single-copy sequences) using hydroxyapatite. The specific activity of both the single- and double-stranded DNA was measured at various CoT values. It was found that the radioactivity was distributed uniformly between the above classes of chromatin DNA. Since 70% of the radioactivity is due to 7-methylguanine, it is most probable that this alkylation product is distributed uniformly too.

Experiments are now in progress to measure the 7-methylguanine directly, and to study the distribution of a minor alkylation product ($O^6$-methylguanine) which is now thought to be involved in carcinogenesis.

**SEX HORMONES AND INTRA-CRANIAL GLIOMATA.** J. W. Hopewell, *Research Institute, Churchill Hospital, Oxford.*

Intra-cranial tumours of glial origin can be induced in the rat by the intra-cerebral implant of 3–4 benzpyrene into the brain so that the pellet of carcinogen impinges on the subependymal plate. This is a mitotically active layer of cells found around the lateral wall of the lateral ventricles.

Deaths from tumour, which occur after a latent period of 9 months or longer, are more common in males than females. Castration or ovariotomy at the time of implantation will significantly reduce the number of animals that develop gliomas. This effect is not corrected by the exogenous supply of testosterone, a finding which implicates sex hormone precursors in glioma induction.

Examination of glioma incidence in man over the period 1961–70 in data supplied by the Liverpool Cancer Registry showed human cerebral tumours to be more common in males. Furthermore, age-related changes in tumour incidence could be linked with sex-hormone changes. In females aged 45 years and over, tumour incidence falls relative to that observed in males, where incidence continues to rise sharply with age. A peak incidence in males is reached in the 60–65-year age group and thereafter it declines. These observations are discussed in the light of the experimental findings in the rat.

**THE INDUCTION OF TUMOURS OF THE DIGESTIVE TRACT AND OF CERTAIN OTHER ORGANS IN RATS GIVEN T-2 TOXIN, A SECONDARY METABOLITE OF *FUSARIUM SPOROTRICHIOIDES*.** R. Schoental, A. Z. Joffe and B. Yagen, *Royal Veterinary College, London and the Hebrew University, Jerusalem.*

Rats were given T-2 toxin (3α-hydroxy-4β, 15 - diacetoxy - 8α - (methylbutyryloxy) - 12,13 - epoxy-$\Delta^9$-trichothecene) which has been isolated from crude extracts of *F. sporotrichioides* by Dr Yagen, in Jerusalem, in pure crystalline form. The rats were white, weanlings obtained from the MRC Laboratory Animals Centre, Carshalton, Surrey. These were given freshly prepared solutions of T-2 toxin, by stomach tube (1–4 mg/kg body weight) and the dosing repeated at various intervals. Among rats, which survived longer than 16 months after the first dose, and 5–8 doses of T-2 toxin, chronic and neoplastic lesions were present, which included: tumours (benign and malignant) of the pituitary, brain, pancreas, mammary gland, as well as adenocarcinoma of the stomach and duodenum. The implications of these results for human health have to be seriously considered (compare Schoental, Joffe and Yagen, 1976, *Br. J. Cancer*, **34,** 310).

**THE PREDICTIVE CAPACITY OF *IN VITRO* DRUG SENSITIVITY TESTS; STUDIES ON A HUMAN TUMOUR XENOGRAFT.** A. Bateman, *Institute of Cancer Research, Sutton, Surrey.*

The aim of our present work is the development of an *in vitro* test to measure the chemosensitivity of cancer cells obtained directly from patients. An assay of clonogenic cells prepared from fresh biopsies of human tumours has been developed in our department and we intend to use it to measure the survival of these cells after incubation with chemotherapeutic agents *in vitro*. Since the parameters of drug exposure *in vitro* differ from pharmacological conditions, we report preliminary experiments to test whether drug toxicity *in vitro* is a good indicator of *in vivo* effect. Cells from a human tumour xenograft (HX32) were exposed to various concentrations of a drug, either *in vitro* or in the host mouse, and the resultant clonogenic cell survival was measured. The cytotoxic effect on HX32 cells was recorded for each of 8 drugs used *in vitro* at concentrations achieved in patient's plasma.

The drugs were then ranked in order of *in vitro* toxicity. Similarly, the 8 drugs were ranked according to toxicity to HX32 tumours in mice injected with 10LD doses.

We conclude that *in vitro* drug sensitivity tests can be used to predict relative *in vivo* activity, if care is taken to use drug concentrations and exposure times *in vitro* which resemble pharmacological exposures.

**ESTABLISHMENT OF PRIMARY HUMAN PANCREATIC CARCINOMA IN CONTINUOUS CULTURE AND IN IMMUNE-DEPRIVED MICE.** A. G. GRANT, D. DUKE and J. HERMON-TAYLOR, *Department of Surgery, St George's Hospital Medical School and Cancer Chemotherapy, ICRF, London.*

An epithelioid cell line derived from human pancreatic exocrine adenocarcinoma has been grown in culture for one year. The cells have a doubling time of 48 h and grow to an almost confluent monolayer, together with a constant population of viable "floating" cells in the growth medium. Evidence of their tumourgenicity is provided by growth on a fibroblast monolayer and in soft agar. Progressively growing solid tumours have been obtained in nude mice following injection of cells in 6th or 8th passage.

Mouse xenografts have also been established directly from primary tumour tissue obtained from two pancreatic cancers, and have been successfully passaged *in vivo*. Each tumour line exhibits different but stable growth characteristics.

An electrophoretic study of the exportable pancreatic digestive enzymes and selected intracellular enzymes present in these tissues has shown that neither the cell line, xenografts, nor the primary tumour tissues produce pancreatic cancer-specific isoenzymes likely to be of clinical diagnostic use.

**THE IMPORTANCE OF SERUM PROTEINS IN HUMAN LYMPHOMAS.** J. G. MCVIE and D. HOLE*, *Department of Clinical Oncology, University of Glasgow and *Cancer Surveillance Unit, Ruchill Hospital, Glasgow.*

One hundred consecutive patients presenting with a diagnosis of lymphoma were studied. Each patient was staged pathologically and treated conventionally with radiotherapy, chemotherapy or both according to stage. Forty patients had Hodgkin's disease and 60 non-Hodgkin's lymphoma. Patients have been followed up from 2 to 7 years. Thirty-two patients have died in that time, and they had significantly lower pretreatment values for serum albumin, serum immunoglobulin M, serum $\alpha_2$-macroglobulin. A 5-year actuarial survival for the hypoalbuminaemic patients is 56% compared to 76% for patients with normal serum albumin ($P = 0{\cdot}01$). Hypoalbuminaemia predisposed to gram-negative infections and episodes of bleeding, but was not associated with gram-positive infection, viral or fungal infections. In contrast, abnormally low levels of IgG, IgM and $\alpha_2$-macroglobulin were found in the group which developed gram-positive infections. Low albumin was not concomitant with weight loss and was an independent variable in prognosis; it was not related to liver involvement, as only 31% of the hypoalbuminaemic group had lymphoma in the liver. Hypoalbuminaemia was found in all stages of lymphomas and in all histologies. Repeated measurement of serum protein including immunoglobulins was of little value in monitoring patients' progress, with the exception that in the group who did not go into remission, the IgM level fell by a mean of 45% from pretreatment values in comparison to 20% fall in the group which went into remission. There was no change in $\alpha_2$-macroglobulin levels with time and treatment, and minimal depression of IgG and IgA. Serum albumin rose in patients who went into remission and fell in patients who went on to die of the disease. Pretreatment estimation of serum albumin, IgM and $\alpha_2$-macroglobulin gives important prognostic information which identifies high-risk patients, who might be managed by intensive support prior to cytoreductive therapy.

**PROGNOSTIC VALUE OF SERUM PROTEINS IN LUNG CANCER.** C. E. NEWMAN, C. H. J. FORD, N. KALSHEKER*, A. R. BRADWELL* and D. BURNETT*, *Surgical Immunology Unit, *Department of Surgery and Immunodiagnostics Laboratory, University of Birmingham, Edgbaston, Birmingham.*

Carcinoembryonic antigen, $\alpha$-foetoprotein, pregnancy-associated $\gamma$-macroglobulin measurements and other serum proteins may be useful in monitoring disease status in cancer patients (Neville and Cooper, 1976, *Ann. Clin. Biochem.*, **13**, 280). Serial measurements of 19 serum proteins have been performed in patients attending a follow-up clinic after radical resection of lung cancers. In 19 patients who developed clinical evidence of recurrence during follow-up, changes in one or all of 9 proteins occurred in 18, and preceded clinical evidence of recurrence in 16. The best markers of disease status were haptoglobin and orosomucoid which were elevated in 15 patients, and increased serum levels of these were found up to 10 months (mean 4·5 months) before suggestive clinical, scan or X-ray evidence of recurrence.

Studies of these two proteins prior to operation have shown a significant correlation between the tumour size and pre-operative serum levels ($P < 0{\cdot}001$) and between pre-operative levels and survival ($P<0{\cdot}01$). Measurements of serum haptoglobin and orosomucoid are of prognostic value prior to operation, and serial determinations after radical resection predict recurrence before other evidence in a high proportion of patients. Haptoglobin has been shown to suppress the *in vitro* responses of lymphocytes to mitogens (Israel and Edelstein, *Israel J. Med. Sci.*, in press), and a primary or secondary role of such proteins in modifying immune status and immunological responses to tumour cells remains to be elucidated.

**GROWTH-HORMONE RESPONSE TO A GLUCOSE-TOLERANCE TEST IN CANCER PATIENTS WITH VARYING DEGREES OF WEIGHT LOSS.** P. F. M. Wrigley, C. H. Collis, D. Fiddian and L. H. Rees, *Department of Medical Oncology and Chemical Pathology, St Bartholomew's Hospital, London, EC1 and Dietetic Dept., Hackney Hospital, London, E9.*

Incomplete suppression or a paradoxical rise in growth hormone (HGH) in response to an oral glucose load (GTT) has been reported as a feature of endometrial carcinoma, and in association with hypertrophic osteoarthropathy in carcinoma of the bronchus. Eighteen patients with a variety of moderately extensive carcinomas were assessed to find out whether there was an association between such abnormal HGH responses and weight loss or anorexia. An assessment of reduction in calorie intake and loss of appetite was made from a full dietary history. Half the patients had lost more than 10% in weight compared with their pre-illness weight, and half less than 10%. Two patients had persistent high HGH levels following the GTT, one of whom also had acromegalic features. Eight patients had a mild paradoxical rise in HGH or persistently moderately elevated HGH. These patients' diagnoses included carcinoma of the colon, rectum, pancreas, bronchus, breast, kidney, adenocarcinomas of unknown origin and Hodgkin's disease. In 8 patients there was normal suppression of HGH. Eleven patients had diabetic glucose-tolerance curves. No relationship between abnormal HGH response and anorexia, weight loss or histological diagnosis was seen.

**IMPROVING CANCER THERAPY TRIALS.** R. Peto, *Reader in Cancer Studies, Radcliffe Infirmary, Oxford.*

There are several hundred cancer trials currently in progress, and many will yield either no information or misleading information, due to errors of design. The commonest errors are:

1. *Inadequate size.* This is a fault of most trials, as in a trial involving only 100 cancer patients medically important differences can easily be swamped by random error. Collaboration with other oncology clinics (perhaps through the MRC collaborative trials) simplified documentation, non-restrictive eligibility criteria and an active effort to make entry as rapid as possible, may all help.
2. *Nonrandomized designs* which allow biases to affect the comparison (*e.g.* using comparisons with past patients instead of with present patients).
3. *Incomplete follow-up,* either by intent (ceasing to follow patients who deviate from protocol) or by default (loss of patients who move). It is not widely enough known that the Government will, for a fee of £1 per patient, provide mortality follow-up from central records for the indefinite future.
4. *Exclusion of "protocol deviants"* from the statistical analysis. The biases that can arise if this is done are discussed in Section 13 of a 67-page review paper on clinical trial methodology recently published in the *Br. J. Cancer* (**34,** 585 and **35,** 1) reprints of which will be made available.

**SEQUENTIAL ACQUISITION OF FIBRINOLYTIC ACTIVITY AND GROWTH IN AGAR IN CULTURES DERIVED FROM RAT BRAINS EXPOSED TRANSPLACENTALLY TO ETHYLNITROSOUREA.** T. A. Hince and J. P. Roscoe, *School of Pathology, Middlesex Hospital Medical School, London W1.*

In a previous study (Hince and Roscoe, 1977, *Br. J. Cancer*, **36,** 401) we have demonstrated that high plasminogen-dependent fibrinolytic activity was associated with cell lines derived from the brains of rats exposed transplacentally to the carcinogen ethylnitrosourea (ENU). The study has been extended with particular emphasis on the timing of the appearance of fibrinolytic activity and its relationship to the ability of cells to form colonies in agar. Two cell lines derived 90–91 days p.i. (45F) and 57–60 days p.i. (45A) were fibrinolytic when tested (24th and 26th transfers respectively) (Hince and Roscoe, 1978, *Br. J. Cancer*, in press) but, at this stage, they did not grow in agar although they became positive at subsequent transfers. More detailed studies using a culture derived 2 days p.i., BE10, and two clones (BE10-13 and BE10-7) derived at the 10th and 20th passages respectively, confirmed the previous observations. BE10 had low activity (measured as the % total of positive colonies with 40–70 cells/colony) to begin with (8%—14th passage) but gained higher activity within a few passages (62·5%—17th passage). However, growth in agar was not demonstrated until the 45th passage. The activities of the two clones, BE10-13 and BE10-7, were similar; both showed relatively high fibrinolytic activities (35–50%) at the 8th transfer, but did not acquire the ability to grow in agar for a further 20 passages (Roscoe and Claisse, 1976, *Nature*, **262,** 314). Thus, in this system it appears that, in culture, characteristics of transformed cells are gained in sequence; fibrinolytic activity being demonstrable before growth in agar.

**CELL SURFACE PROTEOGLYCANS AND GLYCOPROTEINS OF MOUSE BONE MARROW CULTURE.** J. T. GALLAGHER* and T. M. DEXTER†, **CRC Department of Medical Oncology and †Paterson Laboratories, Christie Hospital, Wilmslow Road, Manchester.*

A liquid culture system, in which haemopoietic stem cells can be maintained for several months (Dexter *et al.* (1977) *J. Cell Physiol.*, **91,** 335) provides a unique opportunity for studying problems of stem-cell regulation.

Foremost amongst these is the role of marrow supportive stroma in the determination of stem-cell commitment and proliferation. To this end, glycoproteins and proteoglycans synthesized by the marrow cultures were labelled by incubation with $^{3}H$-glucosamine and $Na_2\,^{35}SO_4$; cell surface and extracellular macromolecules were extracted with trypsin/EDTA and the extract analysed by NaCl-gradient elution from DEAE-cellulose. $^{3}H$-labelled neutral and acidic glycoproteins were detected in the early part of the gradient (0–0·3 M NaCl), followed by two doubly-labelled ($^{3}H$ and $^{35}S$) peaks emerging between 0·5 M and 0·7 M NaCl. Analyses by electrophoresis and chondroitinase digestion showed that heparin sulphate was the main sulphated proteoglycan, with chondroitin sulphate, in all 3 isomeric forms, as the minor component.

Hyaluronic acid was not a major $^{3}H$-labelled macromolecule of the marrow matrix, although small amounts might be present.

**NUCLEAR-CYTOPLASMIC INTERACTIONS IN MAMMALIAN CELLS AND THE USE OF CYTOPLASMICALLY INHERITED RESISTANCE DETERMINANTS.** I. CRAIG and E. MUNRO, *Genetics Laboratory, University of Oxford.*

The availability of cytoplasmically inherited drug-resistant determinants in some strains of mammalian cell lines has enabled the construction of cell types combining nuclei and cytoplasm from different sources. This approach may provide a way to evaluate the importance of nuclear-cytoplasmic interactions to the expression of differentiated characters or to the determination of malignant phenotypes. We have previously described the cytoplasmic transfer of a determinant for chloramphenicol resistance between different human cell lines and observed that there are very distinctive strain and species differences in the ability of cells to act as recipients (Munro *et al.*, 1978, *Proc. R. Soc. Lond. B.*, in press).

We have recently obtained results from fusions between mouse cell lines from strains carrying two different cytoplasmically inherited determinants, chloramphenicol resistance and oligomycin resistance (Lichtor and Getz, 1978, *Proc. Natl. Acad. Sci.*, in press). The results strongly suggest that the hybrids preferentially retain the cytoplasmic determinant (presumptive mitochondrial DNA) from the chloramphenicol-resistant parent. Such observations provide new insights into the interactions between the cell nucleus and cytoplasmic genetic markers.

**PLASMINOGEN ACTIVATION, AND ACTIVATOR ALONE, INITIATE 3T3 CELL GROWTH TO ABNORMAL DENSITIES.** P. WHUR, J. J. SILCOX, J. BOSTON and D. C. WILLIAMS, *Marie Curie Memorial Foundation, Oxted, Surrey.*

Most malignant cells produce high levels of plasminogen activator, but there appears to be little good evidence of a correlation between plasminogen activation and absence of density-dependent inhibition *in vitro.* Confluent, quiescent 3T3 cells subjected to plasminogen activation by the addition of plasminogen and low levels (10 Plough u/ml) of urokinase, grow to super-confluent densities. Addition of urokinase alone has no effect, but at high concentration (200 u/ml) the effects are the same as those produced by plasminogen activation or plasmin; disruption of the monolayer and growth to higher densities. Since urokinase is a protease of trypsin-like specificity, and there is evidence of a close biochemical similarity to plasminogen activator, we propose that the production of high levels of activator alone may be sufficient for transformed cells to grow beyond confluence in the absence of plasminogen.

**PLASMA ZINC AND COPPER: THEIR RELEVANCE TO PROSTATIC DISEASES.** F. K. HABIB, T. C. DEMBINSKI, M. R. G. ROBINSON* and S. R. STITCH, *Division of Steroid Endocrinology, Leeds and *Pontefract General Infirmary, Pontefract, Yorks.*

Total Zn and Cu plasma concentrations were measured by atomic absorption spectroscopy in 62 untreated patients with benign prostatic hypertrophy (BPH) and carcinoma of the prostate (CaP). Although the Zn levels in carcinoma of the prostate were significantly lower than those in the benign tissue (Habib *et al.*, 1976, *J. Endocr.*, **71,** 133) we were unable to correlate the trace metal blood levels with the pathological condition of the gland ($P > 1$). Control studies on a younger group of 14 subjects suggests, however, a progressive increase in plasma Cu levels with age; no similar trends were observed with Zn.

Parallel investigations on 31 prostatic carcinoma patients receiving treatment with stilboestrol, estracyt or tamoxifen were also carried out. A marked depression of plasma Zn ($P < 0{\cdot}01$) and elevation of plasma copper ($P < 0{\cdot}01$) was detected in the endocrine-treated patients. These changes were most pronounced in subjects receiving stilboestrol, suggesting that the levels of the Cu and Zn are profoundly influenced by oestrogens.

### FAECAL BILE ACIDS AND *CLOSTRIDIA* IN THE AETIOLOGY OF COLORECTAL AND BREAST CANCER.

A. Blackwood, W. R. Murray, C. Mackay and K. C. Calman, *Departments of Clinical Oncology and Surgery, Western Infirmary, Glasgow, Scotland.*

Total bile-acid levels and the presence of steroid nuclear dehydrogenating (ND) *Clostridia* of cancer and non-cancer patients from the West of Scotland were compared to investigate the hypothesis that dietary fat has a role in the aetiology of colon cancer (Hill *et al.*, 1971, *Lancet*, i, 95). Breast cancer has a similar epidemiology to colorectal cancer, so patients with this form of cancer were also included in the study. Mean bile-acid levels (μmol/g freeze-dried faeces) for each patient group were as follows:

| Group | No. patients | Concentration | ± s.d. |
|---|---|---|---|
| Colorectal cancer | 29 | 10·42 | ±4·17 |
| (Rectal) | 17 | 10·38 | ±3·6 |
| (Caecal) | 12 | 10·47 | ±4·7 |
| Breast cancer | 18 | 15·02 | ±6·1 |
| Controls | 13 | 15·35 | ±7·7 |

The levels for patients with colorectal cancer were significantly lower ($P < 0{\cdot}05$) than the controls. This difference, however, is opposite to that predicted by the hypothesis. This lack of correlation has been found by another group (Report from I.A.C.R. 1977, *Lancet*, ii, 207). There was no difference between patients with breast cancer and controls. A difference in the occurrence of NDH *Clostridia* between controls and cancer patients was observed. Of the controls 53·8% had *Clostridia*, compared to 83·3% of patients with colorectal cancer and 78·9% of patients with breast cancer. These figures indicate that any relationship between faecal bile acid, bacteria and the incidence of colorectal cancer and breast cancer is not a simple one and requires further study.

### STUDIES ON A POSSIBLE VIRAL AETIOLOGY OF CANINE LYMPHOSARCOMA AND MAMMARY TUMOURS.

S. J. Armstrong, P. Nunes de Souza*, T. G. Wreghitt†, J. Nagington†, B. W. J. Mahy and L. N. Owen, *Depts. of Clinical Veterinary Medicine, *Pathology (Virology) and †Public Health Laboratories, New Addenbrooks Hospital, Cambridge.*

Some evidence for oncornaviral involvement in canine lymphosarcoma was found by Onions, who demonstrated poly-rC oligo-dG templated reverse transcriptase (RT) in 2 of 11 tumours (1976, Ph.D. Thesis, Univ. of Cambridge).

In our experiments, culture supernatants from 3 leukaemic cases were positive by the RT assay. 10/11 lymphosarcomas were also positive, the exception being an animal pretreated by irradiation. $^3$H-uridine-labelled particles of a density of 1·14–1·17 were produced in 3/7 of these cultures. 2/3 leukaemic lymphosarcoma supernatants were positive by both criteria and negative-stained preparations contained particles resembling oncornavirus. The diameter of the virus was 68 nm, whereas that of Rous Sarcoma Virus was 94 nm.

Ten mammary tumours were cultured; 5 were initially positive by RT assay, 4 grew into a monolayer, 1 was continually positive, and this activity was stimulated by progesterone and dexamethasone. Virus particles similar to those from the leukaemic material were seen.

### TUMOUR-NECROSIS FACTOR FROM THE RABBIT: *IN VITRO* STUDIES ON ITS SPECIFICITY, MODE OF ACTION AND SITE OF SYNTHESIS.

N. Matthews and J. F. Watkins, *Department of Medical Microbiology, Welsh National School of Medicine, Cardiff.*

Sera from rabbits injected with BCG and then with endotoxin contain a factor (tumour-necrosis factor—TNF) which, even at high dilutions, is cytotoxic *in vitro* for mouse L cells and some other cell lines but not primary embryo fibroblasts. Sensitivity to TNF could not be correlated with tumourogenicity for several animal and human cell lines tested nor with the production of C-type viruses. TNF was most cytotoxic to metabolically active L cells and acted on both dividing and non-dividing L cells. It was effective at 37°C but not at 22°C or 4°C. Rabbit TNF is remarkably stable with a mol. wt. of 40–50,000. It was eluted with the more acidic proteins on ion-exchange chromatography and precipitated in 50% saturated ammonium sulphate solution. A substance with similar physico-chemical properties and cytotoxic specificity can be obtained from the supernatant of rabbit peripheral-blood monocytes cultured *in vitro*.

**THERAPY OF METASTASIZING RAT TUMOURS OF SPONTANEOUS ORIGIN.** W. A. BASLEY, J. A. GREAGER, R. C. REES and R. W. BALDWIN, *Cancer Research Campaign Laboratories, University of Nottingham.*

Tilorone HCl is an antiviral drug which has been shown to induce high levels of circulating interferon in the rat and is effective both orally and parenterally. Adamson, (*J. Natl. Cancer Inst.*, 1971, **46**, 131) has demonstrated its antitumour activity against the Walker 256 carcinosarcoma and a reticulum-cell sarcoma grown s.c. when the drug was injected i.p. We have used the drug to treat WAB/Not rats bearing the spontaneously arising carcinomas Sp1 or Sp22 which are not known to be of viral aetiology. These tumours will grow in a syngeneic host when implanted s.c. or in the breast pad respectively, and after 10 days growth will metastasize to regional lymph nodes and lungs. The injection of tilorone HCl i.p. at various intervals during tumour growth and metastasis has been found to significantly reduce the number of tumour nodules in the lung and increase the survival time of the animals.

These experiments indicate that tilorone HCl can influence the natural metastases which occur with these spontaneously arising carcinomas.

**INABILITY OF ANTICOAGULANTS TO PREVENT PULMONARY METASTASES IN MURINE BREAST CANCER.** R. C. N. WILLIAMSON, P. J. LYNDON and T. LAI, *Department of Surgery, University of Bristol.*

Warfarin inhibits both spontaneous pulmonary metastases (PM) in mice, and PM induced by i.v. injection of viable tumour cells (Hoover *et al.*, 1976, *Surgery*, **79**, 625; Agostino and Cliffton, 1962, *Arch. Surg.*, **84**, 449). But an increased incidence of extra-pulmonary metastases accompanying similar inhibition of PM by heparin suggests only an altered pattern of tumour dissemination (Hagmar, 1970, *Acta Pathol. Microbiol. Scand.*, **211**, 1). Potential inhibition of PM by anticoagulation with heparin or warfarin was tested in A-strain mice (weight 20 g) receiving a single i.v. injection of 0·5 or $1 \cdot 0 \times 10^6$ viable tumour cells prepared from an A-strain mammary carcinoma. Warfarin was added to the drinking water in doses (3 or 4 mg/l) sufficient to produce full anticoagulation, as tested by Thrombotest (Nyegaard). Graded doses of heparin (125, 250 or 500 u/kg) were given 8-hourly s.c. Anticoagulation was started before tumour injection and continued for 7–14 days thereafter. PM were counted after sacrifice at 14 days.

| | Control (saline) | Heparin | Control (water) | Warfarin |
|---|---|---|---|---|
| No. mice | 18 | 60 | 21 | 37 |
| Mean No. PM/mouse | 137 | 144 | 128 | 142 |
| | NS | | NS | |

Despite causing full anticoagulation, neither heparin nor warfarin affected the development of PM in this tumour model.

**COMBINATION HYPERTHERMIA (42°C) AND HYPERGLYCAEMIA IN THE TREATMENT OF THE YOSHIDA SARCOMA.** S. K. CALDERWOOD and J. A. DICKSON, *Cancer Research Unit, University Dept. of Clinical Biochemistry, Royal Victoria Infirmary, Newcastle.*

The sensitization of neoplastic cells to hyperthermia at low intracellular pH ($pH_i$) was reported by von Ardenne (*Adv. Pharmac.*, 1972, **10**, 339). He described the use of prolonged (3–5 h) hyperglycaemia *in vivo* to stimulate tumour glycolysis and increase lactic-acid production. The low tumour $pH_i$, which he claimed ensued, activated lysosomal enzymes to potentiate hyperthermic damage.

Following hyperthermia (42°C/1 h) the Yoshida sarcoma on the foot of rats regresses in 10–14 days with cure of the host. An increase in blood glucose concentration (mean 400 mg %) for a 4 h period was induced in tumour-bearing rats. Tumour lactate concentration doubled, and extracellular pH, measured by micro capillary glass electrode, fell from 7·2 to 6·6. The intracellular pH of the sarcoma (measured by partitioning of the weak organic acid dimethyloxazolidinedione across the cell membrane) was unaltered and glycolysis decreased. Tumour-regression time following hyperthermia was unaffected.

Hyperglycaemia, therefore, caused inhibition rather than stimulation of glycolysis, and tumour $pH_i$ was a stable property apparently buffered against local increases in lactate concentration. The destructive effects of hyperthermia on the Yoshida tumour were not potentiated by hyperglycaemia.

**CYCLOPHOSPHAMIDE AND REOXYGENATION OF A RAT ADENOCARCINOMA.** B. DIXON and J. V. MOORE, *Radiobiology Department and University Dept. of Radiotherapy, Cookridge Hospital, Leeds.*

Cytotoxic drugs in large doses, tolerable to the host, are often capable of sterilizing a large fraction of cells within a solid tumour (Urano *et al.*,

1972, *Gann*, **63**, 491; Moore and Dixon, 1978, *Eur. J. Cancer*, **14**, 91). This may lead to reoxygenation of surviving hypoxic cells and a consequent improvement in the irradiation response of the tumour. Hence 9±1 mm diameter tumours transplanted s.c. in isogenic female Wistar rats have been irradiated locally with γ-rays alone or, 1, 4, 7 and 10 days after an i.p. injection of 150 mg/kg of cyclophosphamide.

The dose-response data, for delay in growth of the tumour after irradiation alone under normal or hyperbaric conditions of oxygenation, is biphasic and, above 2000 rad, governed by a hypoxic component of cells. Cell-survival assays indicate that the tumour normally contains a hypoxic cell fraction of about 20%, and that this is reduced to 4% or less by hyperbaric oxygenation.

One day after giving Cyclophosphamide the response of the tumour in air-breathing animals is the same as that obtained when the tumour is clamped 15 mins prior to and during irradiation suggesting that cells surviving drug treatment are predominantly hypoxic. Three days later, the hypoxic fraction is reduced but is still significantly raised under normal aerobic conditions. Only by 7 and 10 days had reoxygenation reduced the hypoxic component to pre-drug treatment levels. No indication of a residual hypoxic component under hyperbaric oxygenation was obtained for irradiation at 4 days after drug.

### DIFFERENTIAL LOCALIZATION OF [$^{99m}$Tc]TECHNETIUM-LABELLED LIPOSOMES IN NORMAL AND TUMOUR-BEARING LYMPH NODES OF THE RAT.

V. J. Richardson*, M. P. Osborne†, K. Jeyasingh‡, B. E. Ryman* and J. I. Burn†, *Departments of *Biochemistry, †Surgery and ‡Nuclear Medicine, Charing Cross Hospital and Charing Cross Hospital Medical School, London.*

Using the rat Rd/3 foot-pad tumour model (Carr and McGinty, 1974, *J. Path.*, **113**, 85), a differential localization of interstitially-injected $^{99m}$Tc-labelled liposomes (phospholipid vesicles) in the primary regional lymph nodes (PRLN) with and without tumour infiltration has been observed. The results obtained with small negative (7:2:1, PC:C:PA), positive (7:2:1, PC:C:SA) and neutral (8:2, PC:C) liposomes at 5 and 30 h after injection were as follows:

| Time (h) | PRLN | % Uptake of injected liposomal $^{99m}$Tc per PRLN: Negative | Positive | Neutral |
|---|---|---|---|---|
| 5 | Normal | 0·14±0·03 (7) | 0·82±0·11 (11) | 1·56±0·23 (11) |
| | Metastasis | 0·18±0·05 (12) | 1·15±0·35 (4) | 0·72±0·15 (12) |
| 30 | Normal | 0·27±0·04 (5) | 1·11±0·26 (10) | 1·36±0·27 (12) |
| | Metastasis | 0·19±0·06 (7) | 1·04±0·61 (4) | 0·30±0·14 (11) |

Mean±s.e. No. of animals in brackets. PC=Phosphatidylcholine. C=Cholesterol. SA=Stearylamine. PA=Phosphatidic acid.

Our findings suggest that $^{99m}$Tc-labelled neutral liposomes may be a suitable lymphoscintigraphic agent for the detection of lymph-node metastases, since a marked suppression of uptake of label was observed with neutral liposomes when comparing normal and tumour-infiltrated PRLN ($P<0·01$ at 5 h and $<0·005$ at 30 h). Cationic liposomes may potentially be useful as carriers of therapeutic agents for the treatment of lymph-node metastases as these liposomes localize well in both normal and tumour-infiltrated PRLN.

### *IN VIVO* ASSAY OF THE RADIATION SENSITIVITY OF TUMOUR CELLS, INFLUENCE OF MIXED MODALITIES.

W. Porschen, J. Gartzen, K. Gewehr, H.-J. Weber, L. E. Feinendegen, *Institut für Medizin, Kernforschungsanlage, Jülich, Germany.*

Tumour lysis can be evaluated in the living mouse by externally measuring the rate of loss of tumour-bound DNA tracer. By sequentially labelling the tumour-bearing animals with $^{125}$IUdR and $^{131}$IUdR 50 h apart, the average tumour cells at the time of the second injection are labelled by $^{125}$IUdR and the euoxic tumour cells are specifically labelled with $^{131}$IUdR. Tumour treatment at this stage of labelling permits the observation of the reaction of euoxic cells and average tumour cells. This information adds to results from tumour control and growth delay.

With this technique effects were analysed of $^{60}$Co γ-rays, cyclotron neutrons (E = 6 MeV) misonidazole (500 mg/kg) and hyperthermia (42°C, water bath), or combinations of these. Misonidazole (15 min before irradiation) altered the oxygen-enhancement ratio by a factor of 1·5 for γ-rays and 1·1 for neutrons; when evaluated from tumour growth delay and $TCD_{50}$ misonidazole gave a dose-modifying factor of 1·47 for γ-rays and of 1·2–1·3 for neutrons.

Based on percent tumour regression 100 days after treatment, the enhancement ratio from hyperthermia (after irradiation) was 2·75 for γ-rays (at 10 Gray) and 2·2 for neutrons (at 3·2 Gray). For neutrons combined with misonidazole and hyperthermia the ratio was 2·4.

These results demonstrate that effects of neutron irradiation may be modified by electron-affinic substances and/or hyperthermia.

### OUTGROWTH OF TUMOUR EXPLANTS AS A METHOD OF ASSESSING RESPONSE TO THERAPY.

N. J. McNally and J. de Ronde, *Cancer Research Campaign Gray Laboratory, Mount Vernon Hospital, Northwood, Middlesex.*

There is an urgent need to develop methods for estimating rapidly and quantitatively the response of human tumours to therapy. One possibility is to use short-term explants. Needle biopsies could provide sufficient material to be cut into small pieces and attached to coverslips by means of a coagulum. The assay would then be to score the presence or absence of outgrowth within a given time as a function of the treatment received.

This method has been used to study the response of mouse tumours to radiation. We have compared the results with those based on measurements of $TCD_{50}$ and tumour-cell survival *in vitro* following treatment *in vivo*. In particular, we have measured the effects of clamping the tumour before treatment, and of the hypoxic-cell sensitizer misonidazole. The results suggest that, in at least one of the tumours tested, the explant technique can provide valid information, the effects of the clamp and sensitizer being what would be expected from the other assays.

The method needs to be extended to other types of experimental tumours before use with human tumours. However, besides its practical potential, the method is applicable to problems of tumour radiobiology for which established techniques may not be suitable.

### GROWTH DELAY ANALYSIS FOR THE CELLULAR RADIOSENSITIVITY OF A MURINE TUMOUR.

T. E. Wheldon, A. S. Abdelaal and A. H. W. Nias, *Glasgow Institute of Radiotherapeutics and Oncology, Belvidere Hospital, Glasgow.*

Analysis of radiation growth-delay experiments is complicated by the occurrence of normal tissue damage as well as tumour-cell kill, both effects contributing to the observed delay. We have approached the problem by combining growth-delay data with data obtained from single-dose radiocurability experiments.

For the C3H mouse mammary carcinoma we find the radiation $TCD_{37}$ to be 65 Gy and the mean delay induced by this dose to be 73 days. Since, from Poisson statistics, the $TCD_{37}$ is that dose which leaves one surviving cell on average, and the delay is recorded as time to grow to $10^8$ cells, the mean doubling time can be calculated as 2·75 days.

We may then calculate $D_0$ for the most resistant clonogens from growth delay, for a dose range in which constancy of doubling time is indicated by linearity of the dose-delay relation. In the range 60–70 Gy, with extrapolation number assumed 1–10 and proportion of resistant clonogens assumed 0·01–1·00, the extreme estimates of $D_0$ are 3–5 Gy, with 3·9 Gy the most reasonable number. These findings, which are relatively insensitive to the numerical assumptions, to tumour-size variations and to variations in dose, are consistent with the resistant clonogens being hypoxic cells.

Growth-delay analysis, with knowledge of the single-dose $TCD_{37}$, holds promise as an *in vivo* method for the estimation of cellular radiosensitivity of experimental tumours.

### REPOPULATION OF $\gamma$-IRRADIATED LEWIS LUNG CARCINOMA BY TUMOUR CELLS AND HOST CELLS.

T. C. Stephens, G. Currie and J. H. Peacock, *Institute of Cancer Research, Sutton, Surrey.*

We have studied cellular repopulation in the Lewis lung carcinoma after irradiation with $^{60}Co$ $\gamma$-rays. Colony formation in soft agar was used to evaluate cell survival. After local irradiation (15–35 Gy) two types of colonies were seen, some with tightly packed cells (compact) and some with diffusely scattered cells (diffuse). After whole-body irradiation only compact colonies were seen. It was found that only cell survival calculated from compact-colony counts correlated well with survival obtained by the lung-colony assay and that the compact-colony forming cells repopulated with a doubling time of about 1 day, which agreed well with tumour-volume regrowth. Following local irradiation (15–35 Gy) the number of diffuse-colony forming cells per tumour was reduced to less than 1/100 of the pretreatment level. Diffuse-colony forming cells repopulated the tumours much more quickly than compact-colony forming cells.

We conclude that at least two populations of clonogenic cells are present in the Lewis lung carcinoma, tumour cells which form compact colonies in agar and repopulate the tumour by *in situ* proliferation, and host cells which produce diffuse colonies in agar and can repopulate locally irradiated tumours by infiltration.

### KINETICS OF IMMUNE DESTRUCTION OF MALIGNANT CELLS IN THE RAT: STUDIES WITH $^{125}$IUDR-LABELLED SYNGENEIC CELLS.

M. V. Pimm, *Cancer Research Campaign Laboratories, University of Nottingham.*

The rate of death of syngeneically transplanted rat tumour cells in normal, tumour-immune, and BCG-treated rats has been determined by monitoring urinary excretion of $^{125}I$ from $^{125}$IUdR-labelled cells (Porteus and Munro, 1972, *Int. J. Cancer*, **10**, 112).

With an ascitic hepatoma, rats receiving cells pre-labelled *in vitro* or *in vivo* showed 10–20% urinary excretion of residual $^{125}I$ activity per 24 h period, developing ascitic growths after 15–20 days, comparable to growth from unlabelled cells. Rats receiving heat-killed or irradiated cells lost 35–60% of residual isotope/24 h, starting 24 h after cell injection. Animals immune to the tumour showed a similar, 60%/day loss, but beginning immediately after cell inoculation. I.p. injection of BCG together with labelled cells prevented their growth, but did not accelerate loss of label within a 6-day period. However, rats pre-treated 4–10 days previously with BCG showed increased $^{125}I$ excretion immediately after cell injection. Comparable tests with solid s.c. growths from hepatoma or sarcoma cells showed slight increases in excretion of label, starting 24 h after injection, from inocula prevented from growth by admixture with BCG. Here, however, excretion was not detectably accelerated from heat- or radiation-killed cells, or in tumour-immune animals, over a 6-day period, although with solid i.p. growths urinary loss of label was increased from irradiated or heat-killed cells.

These studies indicate that urinary loss of isotope from labelled cells may be used as an index of the rate of immunotherapeutic destruction of tumour cells in syngeneic models, but suggest that ascitic or i.p. growths may be more easily studied than solid s.c. tumours.

**KINETICS OF *IN VIVO* CELL DESTRUCTION AFTER VARIOUS CHEMOTHERAPIES.** S. Orbach-Arbouys, J. Lheritier, M. B. Castes and P. Pouillart, *Institut de Cancérologie et d'Immunogénétique*, 16 *Av P. V. Couturier, Villejuif.*

A variety of drugs are administered to $BDF_1$ mice after the injection of $10^7$ $^{125}IUdR$-labelled L1210 leukaemic cells. The fall of radioactivity allows us to define 2 types of cell destruction: one, immediate, intense, short-lasting (adriamycin, Ara-C, VM26, MTX, VCR) the other, much less intense, but long-lasting (5FU, Natulan, DTIC). Some drugs belong to an intermediary group (cyclophosphamide, methyl CCNU, CCNU). Drugs of the 1st group, by rapidly eliminating tumour cells, could be considered as good recruiting agents (adriamycin, VCR, in particular). They are the first to be given in combined chemotherapy.

Among the drugs of the 2nd group, some may have an important effect on survival. 5FU injected after $10^7$ L1210 cells doubles the survival. Since we have previously reported the important role of macrophages in tumour-cell destruction, and since the 5FU effect is entirely abolished by $SiO_2$ or BCG given at the same time, we propose as an explanation of our observation that the drug has induced long-lasting alterations in the leukaemic cells which are then progressively destroyed by the macrophages.

**THE EFFECT OF 5-FLUOROURACIL ON THE GROWTH OF ASCITIC, DISSEMINATED AND SOLID FORMS OF A MODEL COLONIC ADENOCARCINOMA.** S. O'Hare and J. A. Double, *Unit for Cancer Research, University of Leeds.*

5-Fluorouracil (FU) is at present the drug most widely used against colonic adenocarcinomas; administration is usually via the i.v. route. This report describes the effects of i.v. FU on 3 biological forms of a mouse model adenocarcinoma.

MAC 15A is a transplantable mouse colon tumour. I.v. injections of tumour-cell suspensions into normal mice produce disseminated tumour foci. S.c. injections produce solid tumours at the site of injection. Mice received a single i.v. dose of 200 mg/kg FU.

This dose produced an 87·7% cell kill against the ascitic form, 41·3% cell kill against the disseminated form, and 56·5% inhibition of the solid form.

Although the percentage cell kill in the ascitic system is more than twice that produced against the disseminated tumour, the relative percentage increase in median survival time was only 3·4%, indicating that an apparent two-fold difference in cell kill has very little biological significance.

Direct comparison of these 2 tumour forms with the solid tumour system is difficult, since we are unable to calculate a percentage cell kill in the latter case. However, the quantitative results obtained in the ascitic and disseminated systems show a good correlation with the qualitative results found in the solid system.

**PHARMACOKINETICS OF 5-FLUOROURACIL.** H. W. van den Berg, R. F. Murphy and B. J. McDermott, *Department of Biochemistry, Queen's University, Belfast.*

The pharmacokinetics of 5-fluorouracil (FU) in patients undergoing combination chemotherapy for breast cancer, has been investigated using a novel GLC electron-capture assay technique which is capable of detecting 20 ng FU per ml plasma. The concentration of FU in plasma fell rapidly during the first hour after i.v. injection. A rapid distribution phase ($t_{1/2}$ 5·0 min, range 3·0–8·0 min) was followed by a second phase ($t_{1/2}$ 11·8 min, range 6·9–22·8 min). During this period a second peak appeared in GLC profiles of plasma extracts, and there is evidence to suggest that it represents 5–6 dihydrofluorouracil, a major catabolite of FU. This is eliminated from the plasma with a half-life of ~1·5 h. A terminal phase in the elimination of FU from plasma has been observed ($t_{1/2}$ ~1·3 h). It is suggested that the second rapid phase of drug elimination reflects metabolism and urinary excretion, and that the terminal phase represents loss of FU in equilibrium with the intracellular anabolic pool, which would include the postulated active metabolite of the drug.

**THIAMINE VITAMIN $B_1$ STATUS IN CANCER PATIENTS AND THE EFFECT OF 5-FLUOROURACIL THERAPY.** M. Soukop and K. C. Calman, *Department of Clinical Oncology, 1 Horselethill Road, Glasgow.*

Major nutritional abnormalities in cancer patients are often recognized, but despite the specificity and sensitivity of the red-cell transketolase method for thiamine status, little is known of any vitamin $B_1$ levels in cancer patients. 5-Fluorouracil (FU) functions as a cocarboxylase antagonist *in vitro* in the transketolase reaction. The aim, therefore, of the present study was to investigate the thiamine status by the transketolase method and the effect of FU therapy in cancer patients. Of 95 cancer patients 36·8% were found to have biochemical evidence of thiamine deficiency, compared to 10·9% of 46 healthy controls ($P < 0·01$). Twenty-four cancer patients had further thiamine estimations performed 24 h after initial i.v. therapy with FU 15 mg/kg body weight. Only 2/14 patients with initially normal thiamine status developed biochemical evidence of deficiency on FU therapy. Both of these had initial borderline deficiency levels. Nine of 10 patients with initial biochemical deficiency developed an exaggerated deficiency state after FU therapy. One patient on long-term weekly i.v. FU therapy developed clinical evidence of thiamine deficiency. Replacement therapy improved the symptoms and signs of the deficiency state, but coincided with rapid tumour progression. In conclusion, cancer patients are at risk of subclinical or clinical thiamine deficiency, which, if present, can be aggravated by FU therapy. Routine prophylaxis with additional thiamine may, however, be detrimental.

**SUBSTITUTED QUINAZOLINES AS ALTERNATIVE ANTIFOLATES.** R. M. Paine, A. H. Calvert, T. R. Jones, B. Grzelakowska-Sztabert and K. R. Harrap, *Dept. Biochemical Pharmacology, Institute of Cancer Research, Sutton, Surrey.*

The value of methotrexate (MTX) in cancer chemotherapy may be compromised by the development of drug resistance. This may be due to diminished cellular uptake via the reduced folate pathway, or to increased intracellular dihydrofolate reductase (DHFR) levels. In the former case, an antifolate utilizing the folate pathway for uptake would overcome resistance, while in the latter, enzyme kinetic considerations indicate that inhibition of both DHFR and thymidylate synthetase may be of value.

We report experiments on two substituted quinazolines, N-[p-[[(2,4-diamino-5-methyl-6-quinazolinyl)methyl]amino]benzoyl]-L-glutamic acid (CB 3703) and N-[p-[[(2-amino-4-hydroxy-6-quinazolinyl)methyl]amino]benzoyl]-L-glutamic acid (CB 3705). CB 3703 inhibits DHFR as effectively as MTX, and also inhibits thymidylate synthetase. Its toxicity to cultured L1210 cells is comparable with that of MTX, and is reversed by thymidine/hypoxanthine or thymidine/folinic acid combinations. It is transported via the reduced-folate pathway, but reaches higher intracellular levels than MTX. CB 3705 also inhibits DHFR and thymidylate synthetase. Its toxicity *in vitro* may be reversed either by folinic acid or by thymidine alone. It is transported via the folate pathway.

These studies indicate that substituted quinazolines may represent useful alternatives to MTX.

**A RAPID METHOD FOR THE DETERMINATION OF PURINE AND PYRIMIDINE NUCLEOSIDES AND BASES IN PLASMA.** G. A. Taylor, P. J. Dady, S. E. Barrie and K. R. Harrap, *Dept. Biochem. Pharmacol., Institute of Cancer Research, Sutton, Surrey.*

We have established that high-dose methotrexate followed by purine/pyrimidine rescue is superior to methotrexate with folinic acid rescue, in terms of antitumour activity. High-dose methotrexate with purine/pyrimidine rescue has now been approved for clinical evaluation. In preparation for this, we have evolved a rapid sensitive method for the determination of circulating plasma levels of relevant nucleosides and bases. We present data showing basal levels of the major purines and pyrimidines circulating in mouse and man, and the half-lives of the rescuing agents in mice.

**CYTOSINE ARABINOSIDE PHARMACOKINETICS IN ACUTE MYELOID LEUKAEMIA.** A. L. Harris, J. Boutagy and D. Harvey*, *M.R.C. Clinical Pharmacology Unit, Radcliffe Infirmary, Oxford and *Univ. Dept. Pharmacology, Oxford.* (Introduced by Dr T. R. Munro.)

There is wide inter-individual variation in cytosine arabinoside (Ara-C) metabolism, with plasma half-lives varying from 5 min to 2 h (Ho and Frei, 1971, *Clin. Pharmacol. Ther.*, **12**, 941). It has been suggested that pharmacokinetic differences may be related to remission induction (Prooijen *et al.*, 1977, *Clin. Pharmacol. Ther.*, **21**, 744).

We have studied Ara-C pharmacokinetics in new patients with acute myeloid leukaemia. Ara-C was given i.v. as a bolus of 2 mg/kg, and serial blood samples taken from 3 min to 6 h afterwards. Plasma Ara-C levels were also monitored in patients receiving i.v. infusions. Plasma samples were assayed by gas-liquid chromatography (GLC) or GLC-mass spectroscopy. The sensitivity of the former was 50 ng Ara-C/ml, of the latter, 1 ng Ara-C/ml.

Twelve patients received boluses. In 8, the drug was eliminated from the plasma in a biphasic or triphasic manner with a terminal $t_{1/2}$ of 7–66 min. In the remaining 4, levels declined below the limit of detection by GLC with apparent $t_{1/2}$ of 2·2–4·7 min. Clearances ranged from 3·9 to 11·6 l/min. Infusions were monitored in 16 patients, including 7 of the 12 who received a bolus. Dosages were 5 mg to 2 g of Ara-C/24 h. The plasma concentrations ranged from 5·4 to 650 ng/ml and clearance from 0·39 to 4·4 l/min. There was no correlation of age, weight, sex, renal function or remission with clearance.

We conclude that rather than adjust doses by body weight, constant i.v. infusion of 65 mg Ara-C/24 h for 5–7 days would give plasma concentrations in the range of 10 ng to 100 ng/ml in all patients here studied.

### CONTINUOUS LOW-DOSE INFUSIONS OF ARA-C IN THE MANAGEMENT OF PATIENTS WITH AML.

C. G. Potter, C. Bunch, M. J. Pippard and A. C. Rowell, *Nuffield Department of Clinical Medicine, Univ. of Oxford.*

The action of Ara-C is mainly S-phase specific, producing inhibition of DNA synthesis by blocking DNA polymerase. Incorporation of the drug into DNA and effects on RNA synthesis may also be cytotoxic, especially at high concentrations. Work presented shows that the sensitivity of AML cells to Ara-C varies widely (as determined by the dose-response of $^3$HTdR inhibition during 30 min incubations) and a differential sensitivity was found at low Ara-C concentrations for many leukaemic marrows compared with normal controls.

The present study on 18 patients attempted to exploit the low-concentration effect. Ara-C was given by continuous infusion at a low dosage (10–80 mg/d) calculated from the individual sensitivity of the leukaemic cells and pharmacological clearance data. Initial progress and subsequent dose-adjustment was determined by the rate of peripheral blast loss, which was considered adequate if in the range (1–3) × (marrow $^3$HTdR-labelling index). Conventional combination chemotherapy replaced the infusions after 10 weeks, though earlier if response was inadequate or toxicity unacceptable. Seventeen patients showed a measurable anti-leukaemic effect of the infusion. Overall remission rates were comparable with other series, and infusions were well tolerated.

### THE EFFECT OF IMMUNOTHERAPY ON PHENYTOIN METABOLISM IN MAN.

H. H. Wan, P. W. Mullen, N. Thatcher and P. M. Wilkinson, *Depts. of Clinical Pharmacology and Medical Oncology, Christie Hospital and Holt Radium Institute.*

Animal studies have demonstrated that immunotherapy will depress hepatic metabolism which is most evident 7–13 days after treatment. Since immunotherapy and chemotherapy are often used together in the treatment of disseminated tumours, we have studied the influence of BCG and *C. parvum* on phenytoin metabolism in man. Serial serum phenytoin concentrations were determined in 8 patients after a single oral dose of phenytoin sodium (Epanutin, Parke-Davis) before and after immunotherapy. Four healthy volunteers served as controls. No significant difference was observed within patients in the area (mg/R/l) beneath the serum concentration/time curves and the values of $K_m$ and $V_{max}$ derived from the individual concentration time curves, after the 2 phenytoin tests. Similarly, no significant difference was observed in the proportion of 5-(p-hydroxyphenyl) to 5-phenylhydantoin excreted in the urine during the first 72 h after administration. We conclude that BCG and *C. parvum* had no effect on phenytoin metabolism.

### IMMUNOGENICITY OF SPONTANEOUSLY ARISING TUMOURS IN INBRED RATS.

J. G. Middle and M. J. Embleton, *Cancer Research Campaign Laboratories, Univ. of Nottingham.*

Tumours arising spontaneously in highly inbred W/Not rats have been established as lines by passaging in syngeneic hosts. The immunogenicity of these tumours has been assessed using *in vivo* challenge-protection methods. Rats were immunized by surgical excision of s.c. growing transplanted tumours, or by implantation of attenuated tumour tissue or cells. After immunization they were challenged with viable suspended tumour cells, initially at low doses; progressively higher doses were administered at regular intervals thereafter until progressive tumour growth occurred. Tumour growth in immunized and control rats was compared to give an assessment of tumour immunity.

A total of 23 spontaneous tumours of various types have so far been studied and 7 of these have proved to be immunogenic. In some cases the levels of immunity induced were very low, but with some tumours it was possible to induce levels of specific immunity equivalent to those commonly observed with chemically induced tumours.

**MAREK'S DISEASE TUMOUR-SPECIFIC ANTIGEN (MATSA) INDUCED BY THE HERPESVIRUS OF TURKEYS (HVT) IN VACCINATED CHICKENS.** P. C. POWELL and M. RENNIE, *Houghton Poultry Research Station, Houghton, Huntingdon, Cambs.*

Since 1970, vaccination against Marek's disease (MD) has effectively controlled this infectious lymphomatous disease of chickens, but the basic mechanism of protection is still obscure. Antibody and cell-mediated immune reactions to MD virus-specific antigens have been observed, but the demonstration that birds immunized with tumour-specific antigens were protected against MD, the pathological observation of lesion regression and the conflicting reports of the presence in vaccinated chickens of lymphocytes cytotoxic to tumour cells, all suggested that an anti-tumour immune response may also be important. Hitherto, however, it has not been possible to demonstrate that vaccine viruses induce the tumour-specific antigen, MATSA. Living cell suspensions prepared from the tissues and blood of chickens vaccinated with HVT were examined for the presence of MATSA-bearing cells, by membrane immunofluorescence. Positive cells formed a small proportion (up to 2%) of total lymphocytes, particularly in the spleen, from 10 days after vaccination until the end of the experiment at 66 days post-vaccination. These low levels of positive cells contrasted with the much higher levels (up to 30%) observed in tissues from chickens infected with a virulent strain of MD virus. The presence of MATSA-positive cells in chickens vaccinated with HVT supports the proposal made by Payne *et al.* (1976, *Int. Rev. Exp. Path.*, **16**, 136) that an immune response against this antigen has an essential role in the mechanism of vaccinal immunity.

**DEVELOPMENT OF A CELL MEDIATED IMMUNE RESPONSE TO ENCEPHALITOGENIC FACTOR IN RATS DURING HEPATOCARCINOGENESIS.** D. J. FLAVELL, J. GOEPEL, C. W. POTTER and I. CARR, *Dept. of Pathology, Weston Park Hospital, Sheffield and the Academic Divn. of Pathology, Sheffield University.*

A delayed hypersensitivity response to encephalitogenic factor (EF) detected with the macrophage migration inhibition (MMI) test has been demonstrated in human cancer patients (Flavell and Potter, 1978, *Br. J. Cancer*, **37**, 1) and in women with dysplastic cervical lesions (Singer *et al.*, 1975, *Br. J. Obst. Gynaecol.*, **82**, 820). The demonstration of such a response in women with dysplasia suggests that lymphocyte sensitivity to EF appears before clinically detectable carcinoma. Using an animal model in the present study we have attempted to define the point during tumour progression at which a delayed hypersensitivity response to EF first becomes apparent.

The development of a spleen-cell response to EF was investigated in Wistar rats during the course of hepatocarcinogenesis with 4-dimethylamino-3-methylazobenzene. A spleen-cell response to EF as determined with the direct MMI test was detected in 2/10 (20%) animals with minimal changes in the liver, in 2/16 (12%) animals with proliferative hepatic changes, in 7/13 (46%) animals with dysplastic lesions of portal tract epithelial cells and in all 5 animals with cholangiocarcinoma. None of a group of 10 control animals showed any response to EF. Abrogation of the spleen cell response to EF by autologous serum was seen in one sensitized animal with proliferative hepatic changes, in all 7 sensitized animals with dysplastic hepatic lesions, and in 4/5 sensitized animals with cholangiocarcinoma.

These results suggest that a spleen-cell response to EF and associated serum blocking activity occurs in association with carcinogen-induced dysplastic hepatic lesions in rats before the appearance of histologically detectable carcinoma.

**TUMOUR-ASSOCIATED ANTIGEN SPECIFICITIES ON 3-METHYLCHOLANTHRENE-TREATED RAT EMBRYO CELLS.** M. J. EMBLETON and R. W. BALDWIN, *Cancer Research Campaign Laboratories, Univ. of Nottingham.*

Freshly prepared cells of whole 18–19-day rat embryos were inoculated for 18 h *in vitro* with 10 μg/ml 3-methylcholanthrene (MCA) or with medium containing its solvent (DMSO). The cells were then washed × 6 and injected i.p. into syngeneic male rats at a dose of $10^8$ cells per rat. This immunization was performed × 4 at 10-day intervals, using a different preparation of embryo cells on each occasion. The rats were bled 10 days after the 4th injection and their serum tested for antibody reacting with cells of established chemically induced rat tumours, using a membrane immunofluorescence test.

Sera from rats immunized with DMSO-treated cells were completely inactive; but sera from rats immunized with MCA-treated cells reacted with established tumours in about 1/6 of the combinations tested. This antibody activity was reproducible, and specific for certain tumours. It is concluded that the individual antigens of chemically induced tumours are not unique, and similar specificities may be reproduced even after short-term carcinogen treatment.

**FACTORS INFLUENCING THE ANTIGENICITY AND IMMUNOGENICITY OF RADIATION-ATTENUATED AMINO-AZO DYE-INDUCED RAT HEPATOMA CELLS.** R. G. DENNICK and M. R. PRICE, *Cancer Research Campaign Labs., Univ. of Nottingham.*

Brief treatment of $\gamma$-irradiated (15,000 R) rat hepatoma cells with various concentrations of glutaraldehyde at room temperature, markedly influences their capacity to immunize syngeneic rats against subsequent challenge with viable hepatoma cells. Exposure to glutaraldehyde (at concentrations from 0·5% to as low as 0·001%) abolished their immunogenicity and prevented tumour-specific-antibody formation. However, treatment with glutaraldehyde did not modify tumour-specific antigens demonstrable by the binding of antibodies in syngeneic immune sera visualized by indirect membrane-immunofluorescence-staining of treated cells, and alloantigen expression remained unaffected as assayed using a quantitative radioisotopic antiglobulin assay. Also, treated cells specifically absorbed antibody from syngeneic sera, as judged by indirect membrane immunofluorescence or using a $^{51}$Cr-release test. Lactoperoxidase-catalysed radioiodination of glutaraldehyde-treated hepatoma cells, followed by polyacrylamide-gel electrophoresis in the presence of sodium dodecyl sulphate and $\beta$-mercaptoethanol, provided evidence for considerable cross-linking of surface-membrane components. It can be concluded that, although the expression of a tumour-specific antigen may be necessary for effective immunoprotection, this alone is not a sufficient criterion for the induction of immunity. More detailed analysis of this model for defining factors influencing tumour-specific antigenicity and immunogenicity will allow identification of the parameters required for the full expression of tumour immunity.

**RESPONSES IN THE DRAINING LYMPH NODE OF TUMOUR BEARING RATS DURING BCG CONTACT THERAPY.** G. ROBINSON, J. A. JONES and R. C. REES, *Department of Pathology, Cancer Research Campaign Laboratories, Univ. of Nottingham and Dept. of Virology, Univ. of Sheffield.*

Using a transplantable rat hepatoma (D192A) model, the histological and immunological changes in the draining lumbar node were investigated after i.m. implantation in the hind limb of syngeneic rats of either hepatoma (D192A) cells alone or an admixture of D192A cells and BCG. Implantation with D192A cells alone produced a large palpable tumour, with little evidence of infiltrating lymphocytes or macrophages, by the 4th week. The draining lumbar node showed an early cell-mediated response in the T-dependent paracortex which was maintained for 3 weeks and then declined rapidly. A humoral response, with activation of the B-dependent cortex and subsequent appearance of circulating specific antibody began in the 2nd week and continued throughout tumour growth. No palpable tumour developed from the admixture of D192A cells and BCG but, a massive granulomatous reaction, in which the predominant cell type was the macrophage, was detected by histology at the implantation site. In the draining node the early paracortical response was greater than that caused by tumour cells alone and the paracortex also contained numerous macrophage granulomas. This cell-mediated response was maintained throughout the period. However, only a slight humoral response was observed. The admixture of D192A cells and BCG protected the animals from both simultaneous and subsequent D192A tumour challenge at the contralateral intramuscular site.

**ERYTHROCYTE ELECTROPHORETIC MOBILITY: EFFECT OF LYMPHOCYTE SUPERNATANTS.** J. E. D. DYSON (introduced by J. P. DICKINSON), *Y.C.R.C. Laboratory, Univ. Dept. of Radiotherapy, Regional Radiotherapy Centre, Cookridge Hospital, Leeds.*

Porzsolt, Tautz and Ax (1975, *Behring Inst. Mitt.*, **57**, 128) have described a study in which tanned and fixed sheep erythrocytes were used as indicator cells in the Macrophage Electrophoretic Mobility (MEM) test. I have made a systematic study of the use of tanned erythrocytes as indicator cells under similar conditions to those employed by Porzsolt *et al.*, employing myelin basic protein (MBP) as "antigen".

My results show that MBP, at a concentration of 330 $\mu$g/ml, reduces the electrophoretic mobility of erythrocytes by ~75%, but that if MBP is pre-incubated for 24 h with lymphocytes from normal subjects, the same concentration of MBP is only able to reduce the mobility by 5 to 10%. If, however, the lymphocytes are from patients with malignant disease, the residual slowing capacity of the MBP, at the same concentration, is about 30 to 40%. These values are dependent on lymphocyte number, MBP, concentration and time of pre-incubation and the influence of these parameters has been determined in order to optimize the difference between the two groups.

As pre-incubation with lymphocytes from patients with malignant disease results in a level of residual slowing some 25 to 30% higher than that observed with lymphocytes from normal controls, this provides a possible method for identifying subjects with malignant disease. My results thus to some extent support those of Porzsolt *et al.* However, I do not consider, as they do, that this is a modification of the MEM test. The results obtained are more consistent with the method depending on a non-immunological property of the lymphocyte.

**IMMUNOLOGY OF NAEVI AND MELANOMA-*IN-SITU*.** A. J. COCHRAN, N. I. L. WILSON, C. E. ROSS, R. M. MACKIE, L. J. OGG and G. TODD, *Departments of Pathology and Dermatology, Western Infirmary, Glasgow.*

Extracts were prepared from 15 malignant melanomas, 10 simple naevi, 4 pieces of perimelanomatous skin, 4 pieces of skin from areas of lentigo maligna and 4 pieces of normal skin. These were tested, using a capillary leucocyte-migration assay, against leucocytes from 52 patients with benign naevi, 57 with malignant melanomas and 98 normal individuals. Melanoma extracts preferentially inhibited the migration of leucocytes from patients with malignant melanomas and naevi. Naevus extracts selectively inhibited the leucocytes of patients with malignant melanoma. Extracts of skin containing cytologically atypical melanocytes (perimelanomatous skin and lentigo maligna) selectively inhibited the migration of melanoma patients' leucocytes, but showed no preferential activity for naevus leucocytes.

**HISTOPATHOLOGICAL SPECIFICITY OF THE LAI MICROTEST IN BRONCHIAL CARCINOMA.** H. M. ANTHONY and C. MILLBAND, *Dept. of Immunology, School of Medicine, Leeds.*

Highly purified plasma-membrane fractions have been prepared from squamous cell, oat cell (small), undifferentiated and adenocarcinomas of bronchus, using the method of Bell and Seetharum (1976, *Int. J. Cancer*, **18**, 605). The antigens have been tested against leucocytes from 4–6 ml blood (preservative-free heparin) in a modification of the LAI microtest (Holt *et al.*, 1975, *J. Immunol. Methods.*, **8**, 277) using RPMI 1640 with 0·1% crystalline BSA.

This paper reports the results of 68 consecutive tests, most using the squamous and oat antigens (67, 68) and a tumour basic-protein preparation (CTA)-cell (61) kindly provided by Dr J. P. Dickinson, and later tests also the adenoca. (30) and undiff. (29) antigens. The squamous, adenoca. and undiff. antigens gave significant inhibition respectively in 29/37 (78%), 2/2, and 3/3 tests in patients with the same histopathology; 4/12, 3/16, 3/14 with other bronchial carcinomas; 2/12, 0/8, 0/8 with other chest diseases (Ca. Bronchus cannot yet be excluded) and none in 4 other cancers or 2 normals. The oat antigen did not show histopathologic specificity. CTA was positive in 30/43 tests in bronchial carcinoma (70%) and in 0/4 other cancers.

Of the 47 tests using a battery of antigens including the histopathologically corresponding antigen in primary bronchial carcinoma, 91% were positive with one or more lung cancer antigen.

**SURFACE ANTIGENS IN ACUTE MYELOBLASTIC LEUKAEMIA: LACK OF TUMOUR SPECIFICITY.** L. TUPCHONG and I. C. M. MACLENNAN, *Nuffield Department of Clinical Medicine, Radcliffe Infirmary, Oxford.*

Immunotherapy trials in AML are partially based on the presumed existence of immunogenic leukaemia-specific antigens. We have attempted to demonstrate the presence of these antigens by producing an AML-specific heterologous antiserum. Ninety-four antisera, produced in rabbits, were analysed by an LDA cytotoxicity test and by a $^{125}I$ labelled anti-immunoglobulin binding assay. The immunizing myeloblasts included the following: untreated whole cells, neuraminidase-treated cells, gluteraldehyde-fixed cells, normal antigen-blocking serum (NABS)-coated cells. Several antisera were produced in rabbits neonatally "tolerized" against normal human lymphocytes and in adult animals previously injected with cyclophosphamide and splenic lymphocytes. The antisera were absorbed with pooled cadaver spleen and tested against paired myeloblasts and PHA-stimulated autologous remission lymphocytes. Removal of activity against splenic lymphocytes produced complete loss of anti-myeloblastic activity in most antisera. The residual activity present against myeloblasts in some antisera was due to putative cycle-associated antigens and to myeloid differentiation antigen. Normal marrow was able to remove this residual activity. It is concluded that leukaemic myeloblasts possess essentially normal antigens and that tumour-specific antigens common to leukaemic myeloblasts, if present on these cells, are either non-immunogenic in the animals studied, or, if immunogenic, their presence is below the level of resolution of the assays used.

**ALLOANTIGEN-UNRESPONSIVE ALLOGENEIC LYMPHOCYTES FAIL TO RESPOND TO ACUTE LEUKAEMIC CELLS WITH AUTOLOGOUS-LYMPHOCYTE ACTIVATING-DETERMINANTS.** J. C. RIDWAY, G. M. TAYLOR, P. T. KLOUDA and R. HARRIS, *Dept. of Medical Genetics, Univ. of Manchester, St Mary's Hospital, Manchester.*

Leucocytes from certain acute myeloid leukaemic (AML) patients with acute disease stimulate proliferation of autologous remission lymphocytes *in vitro*. Hitherto, it has not been possible to distinguish responses to this antigen from spontaneous lymphocyte proliferation. However, remission lymphocytes in which spontaneous proliferative capacity was depressed with 5-bromo-2 deoxyuridine (BUdR) retained their responsiveness to autologous AML cells. This suggests that leukaemic cells may indeed possess some form of "leukaemia antigen".

For such "leukaemia antigen" to have therapeutic significance it should stimulate allogeneic lymphocytes. Though AML cells from one patient stimulated allogeneic lymphocytes in primary mixed cell culture, they failed to stimulate when the allogeneic lymphocytes were rendered unresponsive, with BUdR, to HLA-D antigens on remission lymphocytes from the same patient. This could mean that responses to "leukaemia antigen" involved lymphocytes reacting with normal HLA-D antigens, suggesting that "leukaemia antigen" is itself a modified form all HLA-D, or that lymphocyte activation by "leukaemia antigen" is restricted by the major histocompatibility complex (MHC) to leukaemic cells and responding lymphocytes identical for HLA-D haplotypes. These two alternatives are not mutually exclusive. However, if MHC restriction operates, severe constraints on attempts at "specific" immunostimulation by active immunotherapy with allogeneic AML cells would apply.

### ANALYSIS OF T-CELL FUNCTION IN SPLEENS FROM HODGKIN'S DISEASE (HD) PATIENTS AND CONTROLS.

D. B. JONES, S. V. PAYNE and D. H. WRIGHT. *Univ. Department of Pathology, Southampton Medical School, Southampton General Hospital.*

Previous lymphocyte-marker studies from this laboratory (Payne *et al.*, 1976, *Clin. Exp. Immunol.*, **24**, 280) have described a substantial proliferation of immunoblasts in HD spleens prior to histological evidence of tumour involvement. More recently, de Sousa *et al.* (1977, *Clin. Exp. Immunol.*, **27**, 143) have claimed the sequestration of T lymphocytes in the spleen as part of the pathogenesis of HD. With these observations in mind, we wish to report results from a study of lymphocyte function in cell preparations from HD spleens. Mitogen-inducible cytotoxicity directed against human lymphoid targets was measured in a 4 h $^{51}$Cr-release assay. A particular problem in this assay was non-specific killing, which was identified as being due to foetal bovine serum activation of T lymphocytes. This activity was absent from cells induced in human AB serum. In this system Con-A was more efficient than PHA in inducing cytotoxic effector cells, and T-depleted preparations were always inactive. Cytotoxicity induced in 6 HD spleen-cell preparations was not observed to vary predictably from that induced in control splenic lymphocytes. T-cell helper function assayed by PWM stimulation of Ig production was normal in 2 HD-involved spleens studied. In two HD-uninvolved spleens, PWM induced minimal Ig production over background. In one of these, this appeared to be due to defective B-cell function, since patients' T cells gave a normal help to allogeneic normal B cells. The heterogeneity in T-cell function observed in HD may indicate individual variation in response in this disease, or that sampling occurred at varying times in a progressive disease process.

### HOST CELLS IN HUMAN BREAST TUMOURS.

A. HOWELL, A. SHAALA and N. R. LING, *Departments of Medicine and Experimental Pathology, Univ. of Birmingham.*

As part of a project to assess the prognostic significance of subpopulations of host cells, human breast tumours were enzymically disaggregated and surface-marker characteristics of infiltrating cells assessed. In 10 tumours where the cell recovery determined by DNA estimation was 56% (range 44–87%), lymphocytes represented 9·4% of the population (range 1·8–20·9 predominantly small lymphocytes) and phagocytic cells assessed by latex ingestion represented 4·1% (range 1·3–8·0). In a further 26 tumours where lymphocyte viability exceeded 80% there were 52% T cells (21–81), 17% B cells (3–36), 15% Fc receptor bearing lymphocytes (2–52) and 29% null cells (0–60). Similar proportions of cell types were found in 5 benign tumours (total lymphocytes 15%, phagocytic cells 3·5%, T 58% of lymphocytes and B 15% of lymphocytes). The mean values for each lymphocyte population in malignant and benign tumours are similar to the means for subpopulations of lymphocytes found in peripheral blood, but 7 malignant tumours had T cells below the range, and 4 tumours had B cells above the range for peripheral blood. The mean values for subclasses of B lymphocytes according to surface immunoglobulins were IgA 4%, IgD 5%, IgG 6% and IgM 7%. In any individual tumour, one, 2 or occasionally 3 subclasses could be absent. The possible prognostic importance of selection of subtypes or subclasses of lymphocytes within tumours is being assessed in a follow-up study.

### STEREOSELECTIVE METABOLISM OF THE OPTICAL ISOMERS OF CYCLOPHOSPHAMIDE.

P. J. COX, P. B. FARMER, A. B. FOSTER and M. JARMAN (introduced by D. E. V. WILMAN), *Chester Beatty Research Institute (Institute of Cancer Research), London.*

Cyclophosphamide {CP, [*bis*(2-chloroethyl)-amino]tetrahydro-2*H*-1,3,2-oxazaphosphorine 2-oxide} is chiral at phosphorus and thus exists as enantiomers (optical isomers). Against the murine ADJ/PC6 transplantable solid tumour, the (−)-isomer has twice the therapeutic effect of the (+)-form. CP is initially ring-hydroxylated, 4-hydroxycyclophosphamide being the only major metabolite when washed liver microsomes are used. This transformation was studied by following substrate disappearance, using stable isotope dilution-mass spectrometry. Substrates were pseudoracemates composed of 1:1 mixtures of (−)-CP-$d_0$:(+)-CP-$d_4$ or (+)-CP-$d_0$:(−)-CP-$d_4$, for which deuterium atoms were located in the chloroethyl groups.

A small degree of substrate selectivity was shown by rat liver microsomes, the (−)-CP being metabolized ~10% faster than the (+)-CP. This preference was reversed with mouse liver microsomes. However, rabbit tissue was markedly stereoselective for (−)-CP, the initial rate of metabolism and extent of substrate disappearance being >3× that of (+)-CP.

Secondary oxidation *in vivo* of the enantiomers of 4-hydroxy-CP by mice showed marked substrate stereoselectivity in the formation of 4-ketocyclophosphamide, but not for the major metabolite, carboxyphosphamide. The lower therapeutic efficiency of (+)-CP may be associated with the more abundant formation of these deactivation metabolites, particularly 4-keto CP, from the (+)-isomer.

**AN INVESTIGATION INTO THE CONFORMATION AND BASE SEQUENCING OF DNA FROM YOSHIDA TUMOURS SENSITIVE AND RESISTANT TO METHYLENE DIMETHANE SULPHONATE (MDMS).** D. G. Poppitt and B. W. Fox, *Paterson Laboratories, Christie Hospital and Holt Radium Institute, Manchester.*

An unusual effect of the alkylating agent methylene dimethanesulphonate (MDMS) with DNA has been a GC-dependent increase in a pretransitional hypochromism. The irreversibility of this effect suggests that the agent becomes associated with the DNA, but not by initially interacting covalently, which causes the strands to separate spontaneously. Such regions could be observed by the cell repair systems as damaged. We have previously shown that the MDMS-resistant line has an increased capacity to repair damage due to the drug, as well as having a template activity which is less sensitive to the same drug. We have now examined the DNA from both these cell lines, and differences in the conformation and base sequencing have been found, by physico-chemical and DNA-hybridization techniques. There is a 15–16% increase in the degree of GC clusters in the resistant line.

**METABOLIC DIFFERENTIATION OF SUBSTITUTED DIMETHYLPHENYLTRIAZENES WITH TUMOUR-INHIBITORY ACTIVITY.** G. F. Kolar, T. A. Connors* and P. M. Goddard†, *German Cancer Research Centre, Heidelberg, F.R.G.*, **MRC Laboratories, Carshalton, Surrey and* †*Chester Beatty Research Institute, London.*

The mechanism by which 3,3-dimethyltriazenobenzenes inhibit tumours is not clear, but it does depend on their metabolic conversion to reactive intermediates. Metabolic studies using 3,3-dimethyl-1-phenyltriazene, 1-(4-chlorophenyl)-3,3-dimethyltriazene and 1-(2,4,6-trichlorophenyl)-3,3-dimethyltriazene, compounds listed according to their increasing activity against TLX5 lymphoma, revealed that progressive ring-chlorination leads to (a) retarded metabolite excretion, (b) altered hydroxylation pattern, (c) decreased formation of anilines but (d) increased excretion of metabolites with intact diazoamino structure (Kolar and Schlesinger, 1976, *Chemotherapy*, **8**, 91). Triazene metabolites were converted into the corresponding 4-areneazo-N-ethyl-1-naphthylamines which were identified. 1-(2,4,6-Trichlorophenyl)-3,3-dimethyltriazene (IST 79%) did not yield any ring-conjugated metabolites, but was excreted as a glucuronoside conjugated through the terminal dimethylamino group (8–10%). The metabolite was isolated by adsorption on anionic and cationic exchangers and purified by gel filtration on Sephadex. Acid hydrolysis liberated glucuronic acid and formaldehyde which indicated the presence of a masked N-methylol group. It is conceivable that this metabolite represents a stabilized transport form of the activated triazene. Chemical and spectroscopic evidence for the proposed structure and for the biological function of the metabolite will be presented.

**1 - ARYL - 3 - HYDROXYMETHYL - 3 - METHYLTRIAZENES: NEW CYTOTOXIC DERIVATIVES OF TRIAZENES.** J. A. Hickman, A. Gescher, M. F. G. Stevens, K. Vaughan and R. Simmonds, *Cancer Chemotherapy Group, Department of Pharmacy, Univ. of Aston in Birmingham.*

1-Aryl-3,3-dimethyltriazenes are selective cytotoxic agents with a broad spectrum of activity against animal tumours (Connors and Hare, 1975, *Biochem. Pharmacol.*, **24**, 2133). It has been proposed that N-demethylation to N-

monomethyltriazenes is essential for cytotoxicity (Connors *et al.*, 1975, *Biochem. Pharmacol.*, **25**, 241). However, we have shown that a typical monomethyltriazene, 1-methyl-3-*p*-tolyltriazene, although cytotoxic, is not selective (Hickman and Vaughan, *Br. J. Cancer*, in press). N-Demethylation of N,N-dimethyltriazenes has been suggested to occur via the generation of a hydroxymethyl intermediate, which then loses formaldehyde (Preussmann *et al.*, 1969, *Biochem. Pharmacol.*, **18**, 1). We now report the preparation of 1-aryl-3-hydroxymethyl-3-methyltriazenes which were previously considered to be highly unstable intermediates. These compounds are cytotoxic *in vitro*, unlike their progenitors the dimethyltriazenes, and have activity *in vivo* against the TLX5 lymphoma, comparable to the dimethyltriazenes. Under physiological conditions the hydroxymethyltriazenes liberate formaldehyde and it is considered that, as in studies on the mechanism of action of hexamethyl-melamine (Connors and Rutty, 1977, *Biochem. Pharmacol.*, **26**, 2385), this might be important in explaining the selective cytotoxicity of the triazenes.

**METHYLMELAMINES: DEMETHYLATION AND ANTITUMOUR ACTIVITY.** C. J. Rutty and K. R. Harrap, *Department of Biochemical Pharmacology, Institute of Cancer Research, London.*

The structure-activity relationships of a series of derivatives of hexamethylmelamine (HMM) have been studied using a mouse plasma-cell tumour (PC6) and a human lung-tumour xenograft (P246). A correlation between the ability of the methylmelamines to undergo demethylation *in vitro* and their antitumour activity *in vivo* has been established. Three derivatives, namely, pentamethylmelamine (PMM) pentamethylmonomethylolmelamine, and trimethylolmelamine have shown marked antitumour activity. The cytotoxicity of HMM to TLX/5 lymphoma cells *in vitro* depends on microsomal activation, whereas PMM-methylol is toxic *per se*. The toxicity in both cases can be almost completely reversed by pretreatment of the cells with semicarbazide, suggesting that formaldehyde is the cytotoxic agent. HMM and PMM inhibit the synthesis of DNA and RNA by TLX/5 cells, which inhibition can be increased by microsomal activation, and reversed with semicarbazide. The two methylols, at lower concentration, cause almost complete inhibition of nucleic-acid synthesis, an effect which is shared by formaldehyde. It is possible to protect selectively against the PMM-methylol inhibition of pyrimidine deoxyribonucleoside precursor incorporation with semicarbazide. There appear to be two sites of action: one due to formaldehyde, and the other to the intact melamine nucleus.

**ALKYLATING AGENTS AND NUCLEOSOME INTEGRITY.** R. Wilkinson and K. R. Harrap, *Dept. Biochemical Pharmacology, Institute of Cancer Research, Sutton, Surrey.*

Chlorambucil, melphalan and cyclophosphamide induce loss of heterochromatin structure in alkylating-agent-sensitive tumour lines (Yoshida, Walker). This effect is not seen in resistant lines of these tumours. We wish to report here that the basic structural component of chromatin, the nucleosome, appears unaffected by these treatments. This observation is based on the nucleotide fragmentation patterns produced by micrococcal nuclease digestions of whole nuclei, which demonstrate the presence of a repeating unit of 200 nucleotides. Further evidence is derived from studies on the chromatin enzyme poly-ADP-ribose polymerase, which is associated with intact nucleosome oligomers (1977, *Biochemistry*, **16**, 506) and possibly with DNA repair (1977, *Cancer Res.*, **37**, 3006). The activity of this enzyme in sensitive cells is unaffected by treatment with alkylating agents, though in resistant cells recovering from damage by alkylating agents, the activity is considerably enhanced.

It would appear, therefore, that alkylating agents affect the tertiary structure of the chromatin and not nucleosome integrity.

**THE COMPARATIVE PHARMACOKINETICS OF PREDNIMUSTINE AND CHLORAMBUCIL.** H. Newell, R. Wilkinson and K. R. Harrap, *Dept. Biochemical Pharmacology, Institute of Cancer Research, Sutton, Surrey.*

We have previously shown, in the rat, that the toxicity of chlorambucil can be reduced, without compromising its antitumour effectiveness, if it is given in the form of the prednisolone ester, prednimustine (1974, *Eur. J. Cancer*, **13**, 873). The pharmacokinetics of chlorambucil and prednimustine were compared, in the rat, in an attempt to explain this lower toxicity. Following its s.c. administration, plasma levels of chlorambucil and its major metabolite phenyl acetic mustard, each decayed with half-lives of 2·1 h. On the other hand, levels of chlorambucil and phenyl acetic mustard, produced from an equivalent dose of prednimustine (also given s.c.) are maintained relatively constant for ~40 h at 1/20th the peak level achieved from chlorambucil alone; no unhydrolysed prednimustine was detected in the plasma. The lower toxicity of prednimustine could be simulated in part by low-dose repeated administration of chlorambucil alone, with no loss of antitumour activity. In man, oral chlorambucil administration produced detectable plasma levels of both chlorambucil and its metabolite phenyl acetic mustard, which persisted for 4 h. Following an equivalent dose of prednimustine, no chlorambucil of phenyl acetic mustard was detectable. These data indicate that the antitumour selectivity of alkylating agents can be increased by using a multiple low-dose regimen. A re-evaluation of current single, high-dose alkylating-agent therapy in man may therefore be indicated.

### THE EFFECT OF ANAESTHETICS ON MELPHALAN CYTOTOXICITY.

J. H. PEACOCK and T. C. STEPHENS, *Institute of Cancer Research, Sutton, Surrey.*

We have studied the influence of anaesthetics on tumour cell kill by the cytotoxic agent Melphalan. B16 melanoma was treated *in vivo* and cell survival was measured *in vitro* using a colony forming assay. Three anaesthetics were employed, Saffan, Nembutal and Hypnorm. When Saffan (90 mg/kg) was administered 20 min before Melphalan (2 to 20 mg/kg) there was a significant increase in the cell-killing effect of this agent. This was not found with Nembutal or Hypnorm. The effect of varying the dose and time of administration of Saffan relative to a fixed dose of Melphalan (7·5 mg/kg) was examined. The enhancement of Melphalan cell kill by Saffan increased as the dose of Saffan was raised (15–150 mg/kg). The optimum time of administration of Saffan (90 mg/kg) for maximum enhancement of cell kill was 15–30 min before Melphalan. When Saffan was administered after Melphalan no enhancement was seen.

It has been suggested that steroid hormones may modulate the effect of Melphalan, and since Saffan is a steroid, this may account for the observed effect.

### THE PHARMACOKINETICS AND METABOLISM OF CHLORAMBUCIL IN PATIENTS WITH CHRONIC LYMPHOCYTIC LEUKAEMIA.

A. McLEAN, *Dept. of Chemistry, Univ. of Surrey, Guildford*, R. WOODS and D. CATOVSKY, *MRC Leukaemia Unit, Hammersmith Hospital.*

Five patients have been studied after oral administration of $^{14}$C-chlorambucil in doses of 15–30 mg/m$^2$. Absorption has been found to be consistently rapid, and the maximum concentration of plasma radioactivity/ml varied between 50 and 70 min. After i.v. administration the rate of elimination of radioactivity from the plasma has been found to be rapid. Urinary excretion was studied up to 3 days after drug administration, and total radioactivity in the urine varied between 28 and 68%. In all patients studied, the bulk of urinary excretion occurred during the first 24 h.

Chromatographic analysis of blood extracts indicates that the human metabolism of chlorambucil follows an analogous course to the reported animal metabolism (A. McLean *et al.*, 1976, *Biochem. Pharmacol.*, **25**, 2331). In all patients studied $\beta$-oxidation of the butyric acid side chain of chlorambucil was observed. Analysis of urine samples obtained shortly after oral and i.v. administration indicated the absence of chlorambucil and the presence of traces of metabolites chromatographicallyindistinguishable from bis and mono 2-chlorethyl derivatives of p-aminophenyl acetic acid.

The information obtained from the plasma-radioactivity measurements, coupled to the analytical data, indicates that the oral route of administration of chlorambucil is not disadvantageous with respect to the i.v. route. The rate of metabolism is sufficient to allow the metabolite material to contribute significantly to the activity of the drug.

### A FLOW CYTOFLUORIMETRIC ASSAY FOR CELLULAR ESTERASES IN POPULATIONS OF SINGLE CELLS.

S. H. CHAMBERS, P. WORKMAN, J. V. WATSON and N. M. BLEEHEN, *MRC Clinical Oncology Unit, The Medical School, Hills Road, Cambridge.*

A flow cytofluorimeter has been linked directly to a PDP 11/40 computer in order to measure the rate of hydrolysis of fluorogenic substrates in whole cells.

Cellular esterases have been assayed in log and plateau-phase EMT6 cells using fluorescein diacetate as substrate.

At the maximum substrate concentration of 2·0 $\mu$M 28-day-old plateau-phase cells showed a 5-fold increase in enzyme activity, compared with 3-day-old log-phase cells. Over the same time interval, plating efficiency fell from 95 to 50%.

Data obtained with whole homogenates using standard spectrofluorimetric assay gave normal Michaelis-Menten kinetics. That obtained from whole cells in the same instrument, showed marked deviation from normal kinetic behaviour and agreed with the curves obtained from whole cells in the cytofluorimeter.

Inhibition of esterase activity by chlorambucil was less in whole cells than in homogenates but inhibition by nitrogen mustard was approximately equal in the two preparations. Again, data from whole cells using standard spectro fluorimetric methods agreed with the computer-linked cytofluorimetric analysis.

**EFFECTOR CELLS AT THE SITE OF IMMUNOTHERAPY-INDUCED TUMOUR REGRESSION.** R. A. ROBINS, D. G. HOPPER and R. W. BALDWIN, *Cancer Research Campaign Laboratories, Univ. of Nottingham.*

A methylcholanthrene-induced rat sarcoma (Mc7) when transplanted in syngeneic recipients, was shown to be infiltrated by macrophages (~20%) and lymphoid cells (7–8%); there was little change in the proportions of host cells during progressive tumour growth. Small tumours developed then regressed in rats given active immunotherapy consisting of an s.c. injection of sarcoma cells in admixture with BCG at a contralateral s.c. site. In these regressor tumours, a high proportion of lymphoid cells was present at the onset of tumour rejection. Effector-cell preparations enriched for tumour-derived lymphoid cells were prepared from regressor tumours by velocity sedimentation and nylon fibre column filtration. When tested in a $^{75}$Se-Selenomethionine microcytotoxicity assay, these tumour-derived effector cells were cytotoxic for cultured tumour cells. A component of this cytotoxic activity was apparently specific for Sarcoma Mc7. Tumour-derived effector cells have also been prepared from progressively growing Mc7 tumours. Interestingly, preliminary experiments indicate that lymphoid cells from progressor tumours may be equally cytotoxic to those from regressor tumours. These findings indicate that lymphoid cell accumulation in the tumour is important for tumour rejection.

**STUDIES ON TWO TUMOUR IMMUNOTHERAPY MODELS: INDUCTION OF SPECIFIC SYSTEMIC IMMUNITY AND IMMUNOSTIMULATION BY DERMAL APPLICATION OF ADJUVANT.** N. WILLMOTT, E. B. AUSTIN, M. V. PIMM and R. W. BALDWIN, *Cancer Research Campaign Laboratories, Univ. of Nottingham.*

Two immunotherapeutic modalities have been developed for treating s.c. challenge inocula of MCA-induced sarcomas in the rat, and an assessment made of their efficiency in treating post-surgical lymph node metastasis from a spontaneous mammary carcinoma Sp4 (Greager and Baldwin, 1978, *Cancer Res.*, **38**, 69). S.c. injection of 230 $\mu$g of *C. parvum* (Wellcome, CN6134) mixed with viable or irradiated tumour cells ($2 \times 10^6$) elicited specific systemic immunity capable of controlling a distant s.c. challenge ($10^6$ cells) of an immunogenic rat sarcoma injected on the same day as the vaccine. Alternatively, repeated administration of BCG (Pasteur F) by the dermal route, starting on the same day as tumour challenge, was able to control an s.c. inoculum of a different immunogenic rat sarcoma ($10^5$ cells). However, in preliminary experiments with a spontaneous mammary carcinoma, designed to treat lymphnode metastasis appearing after removal of the primary tumour, it was found that immunotherapeutic regimes effective at treating s.c. inocula of immunogenic MCA-induced tumours were not effective at treating lymphnode metastasis.

**SEQUENTIAL MONITORING OF CIRCULATING IMMUNE COMPLEXES IN TUMOUR-BEARING RATS AND RATS GIVEN SUCCESSFUL CHEMOTHERAPY OR ACTIVE IMMUNOTHERAPY.** P. J. MCLAUGHLIN, K. HÖFFKEN, M. R. PRICE, V. E. PRESTON and R. W. BALDWIN, *Cancer Research Campaign Laboratories, Univ. of Nottingham.*

Circulating immune complexes were measured in rats bearing s.c. transplanted 3-methylcholanthrene induced sarcomas using the $^{125}$I-Clq-binding inhibition assay of Fletcher and Lin (1977, *J. Immunol. Methods*, **15**, 39). Sera from rats receiving chemotherapy or active immunotherapy (BCG + tumour cells) were also sequentially monitored for immune complexes.

With animals bearing progressively growing tumours, Clq-binding activity was detected in the early phase of tumour growth, but, after reaching a maximum, decreased in spite of tumour progression. This profile of Clq binding was not, however, observed when tumour growth was controlled by chemotherapy or active immunotherapy. In these cases no significant levels of immune complexes were detectable. In contrast, animals failing to respond to therapy had immune-complex levels similar to those of tumour-bearing rats.

Studies to characterize the circulating immune complexes have indicated that they are, at least in part, of a tumour-specific nature, and that the apparent fall in immune-complex levels in late stages of tumour growth is due to changes in antigen-antibody ratio, and a shift from complement-fixing to non-complement-fixing antibodies.

The present findings suggest that measurement of Clq-binding serum factors may provide a useful method for monitoring the growth and burden of experimental animal tumours.

**THE GROWTH OF HUMAN URINARY BLADDER CARCINOMAS, AS PULMONARY NODULES, FOLLOWING I.V. INJECTION OF TUMOUR CELLS INTO IMMUNE-DEPRIVED MICE.** K. COOPER, M. O. SYMES and C. R. TRIBE, *Department of Surgery and Pathology, Univ. of Bristol.*

The growth of human bladder carcinomas, in immune-deprived mice, has been reported

(Kelly *et al.*, 1975, *Br. J. Cancer*, **31**, 237; Franks *et al.*, 1976, *Br. J. Cancer*, **33**, 112). CBA-PC mice were subject to thymectomy at 6–10 weeks of age. Two to 16 weeks thereafter the mice received 900 rad whole-body irradiation. Haemopoiesis was restored by i.v. injection of $5 \times 10^6$ CBA marrow cells (pre-incubated in immunosuppressive anti-thymocyte serum) within 6 h of irradiation.

The mice were each challenged 4 weeks after irradiation with $10^6$ cells from a suspension made from 1 of 4 bladder carcinomas. The tumour cells were injected i.v. to facilitate the development of pulmonary tumour nodules. The mice were killed 4 weeks after injection of tumour cells, their lungs fixed in Bouin's fluid and the number of nodules counted by eye.

Eight or 16 weeks were the optimum intervals between thymectomy and irradiation to promote the development of pulmonary nodules. At the 8-week thymectomy-to-irradiation interval there was a good correlation (Rank Difference Coefficient = 0·7) between the number of nodules formed and the malignancy of the tumour obtained from the patient, as assessed histologically.

### DETECTION OF NORMAL CELL KILLING AGAINST RAT TUMOUR CELLS: *IN VITRO* AND *IN VIVO* STUDIES.

R. C. Rees* and C. G. Brooks†, **Dept. of Virology, Univ. of Sheffield Medical School and* †*Cancer Research Campaign Laboratories, Univ. of Nottingham.*

Lymphnode cells (LNC), prepared from normal rats, could either enhance or inhibit the *in vitro* growth of tumour cells derived from syngeneic chemically induced sarcomas and hepatomas. Fractionation of LNC revealed that the growth-enhancing activity predominated in the cell population eluted from nylon-wool columns, whereas the growth-inhibitory activity was largely a property of nylon-wool-adherent cells. When column-fractionated LNC were cultured, in the absence of tumour cells, the resulting cell-free supernatants caused the respective inhibition or enhancement of tumour-cell growth previously shown by whole LNC. The tumour-growth-inhibitory material was heat stable at 56°C, and was present in the supernatant after 1 h of culture, whereas growth enhancement was only observed in supernatants after prolonged *in vitro* LNC culture.

Experiments performed *in vivo*, using nylon-wool-column-enriched LNC fractions indicate that LNC sub-populations may cause tumour-growth inhibition or enhancement. Normal rat LNC, which showed NK activity towards target cells *in vitro*, were shown to retard the development of tumours when adoptively transferred, in mixed inoculum with tumour cells, into syngeneic recipients.

The results obtained suggest that apparently uneducated lymphoid cells are involved in controlling tumour growth, and that soluble factors released from these cells contribute to the observed effects.

### EFFECT OF INTRAVENOUS *CORYNEBACTERIUM PARVUM* (CP) ON CYTOTOXIC EFFECTOR CELLS IN THE RAT.

E. C. Purves, *Dept. of Experimental Pathology, St Mary's Hospital Medical School, London.*

We looked at the effect of i.v. CP on the non-specific killer cell (NK) activity (Ono *et al.*, 1977, *Nature*, **266**, 546) and the antibody-dependent cell-mediated cytotoxic (ADCMC) activity (McDonald and Bonnard, 1975, *Scand. J. Immunol.*, **4**, 129) of cells from rat blood and spleen, before and after removal of macrophages with iron powder.

CP treatment was followed by a large increase in NK activity. After removal of phagocytes, the actual degree of cytotoxicity was reduced, but the difference between cells from treated and untreated rats was increased, showing that CP increases the activity of the NK cell, and that macrophages can act as effector cells of this type. ADCMC activity was, on the other hand, only slightly increased in cell suspensions from CP-treated rats, but removal of phagocytes caused an increase in this type of cytotoxicity.

Again the increase was greater for cells from CP-treated rats. Thus macrophages can inhibit ADCMC assays, and CP treatment enhances the activity of both phagocytic and non-phagocytic effector cells.

### INTRAPLEURAL (I.PL.) INJECTION OF *C. PARVUM* (CP).

M. T. Scott and J. Decker, *The Wellcome Research Laboratories, Beckenham, Kent.*

In collaboration with the Ludwig lung-cancer trial (EORTC Newsletter, **55**, May 1977) the characteristics of CP injected i.pl. in mice, and its effectiveness against experimental lung-associated tumours have been investigated. After i.pl. injection most of the CP was confined to the pleural and mediastinal spaces, the pleural phagocytes and mediastinal lymph nodes being heavily labelled. None was found within the lung. Some CP did, however, leak systemically, and was found in the spleen and liver. Splenomegaly 14 days after i.pl. CP was similar to that after i.v. injection. I.pl. CP was capable of engaging both immunological-specific (lymphocyte mediated) and non-specific (activated-macrophage mediated) antitumour mechanisms. (a) *Non-specific:* a large proportion of the cells present in pleural washouts 4 days after i.pl. CP were activated macrophages which non-specifically inhibited the proliferation of tumour cells *in vitro*. (b) *Specific:* local interaction of CP with tumour antigen in the pleural cavity (i.pl. CP mixed with irradiated tumour cells) conferred strong, specific, systemic immunity against subsequent live tumour-cell challenge, whereas CP or tumour antigen alone are without effect. The immunity was detectable in cell suspensions prepared from the mediastinal node. I.pl. CP has been compared with i.v. CP for its effect against tumours growing either in the lung or in the pleural cavity. I.pl. CP was more effective than i.v. CP against tumour growing in the pleural cavity. I.v. CP was more effective than i.pl. CP in reducing the number of lung metastases resulting from i.v. injection of tumour cells, but survival of mice was consistently longer after I.pl. CP.

**INTRAVENOUS BCG THERAPY OF MAMMARY CARCINOMA IN BITCHES AFTER SURGICAL EXCISION OF THE PRIMARY TUMOUR.** N. T. GORMAN, D. E. BOSTOCK and L. N. OWEN, *Dept. Clinical Veterinary Medicine, Univ. of Cambridge.*

Thirty-four bitches with defined histological forms of mammary carcinoma were studied post-mastectomy for over 100 weeks. They were randomized into 3 groups:

(a) no further treatment (11)
(b) placebo treatment (10)
(c) i.v. BCG therapy (13).

The median survival time of the control groups was 24 weeks, whereas that for the treated groups was 100 weeks. This was significantly different ($P < 0{\cdot}05$) as calculated by the Chi-square test.

**EFFECTS OF *C. PARVUM* IN DOGS AND A STUDY OF ITS DISTRIBUTION FOLLOWING INTRAVENOUS INJECTION.** J. C. M. LEWIS, L. N. OWEN, D. R. MORGAN and N. T. GORMAN, *Dept. of Clinical Veterinary Medicine, Univ. of Cambridge.*

Studies were made in dogs to determine the distribution of *Corynebacterium parvum* (CP) after its i.v. administration, its pathological effects and the possible cytotoxicity of alveolar macrophages thus stimulated.

The distribution of CP was followed using $I^{125}$ and FITC labelling techniques previously described by Scott and Milas (1977, *Cancer Res.*, **37**, 1673). Alveolar macrophage cytotoxicity was studied by release of isotope from $Cr^{51}$-labelled allogeneic target cells.

Persistence of labelled CP was most marked in the liver, spleen and lungs, but was eliminated faster from the latter than from the other two organs. The pattern of distribution was similar to that of the mouse (Scott and Milas, 1977, *Cancer Res.*, **37**, 1073 and Sadler *et al.*, 1977, *Br. J. Cancer*, **35**, 357) but elimination was faster.

Gross pathological changes following i.v. CP were few and histological changes restricted to small granuloma in liver and lungs. *C. parvum* vaccine given s.c. or i.m. produced local reactions, but not if diluted.

Alveolar macrophages from dogs injected with CP exhibited some significant target-cell cytolysis when compared with macrophages from normal dogs.

Given that i.v. CP follows a similar pattern in dogs and mice, it would appear likely that a similar distribution would occur in man. It would be advantageous to use i.v. CP if it can be demonstrated as being as effective as BCG in delaying metastasis in the dog.

**INTRA-PLEURAL BCG IN OPERABLE LUNG CANCER.** P. B. ILES, D. F. SHORE, R. W. BALDWIN and M. J. S. LANGMAN, *Depts. of Therapeutics and Cancer Research, Univ. of Nottingham, and Thoracic Surgery, United Sheffield Hospitals.*

Following the report of McKneally *et al.* (1976, *Lancet*, i, 377) of the benefit of BCG (Tice) given intra-pleurally to patients after resection for lung cancer, we instituted a controlled, prospective randomized study of intra-pleural Glaxo BCG in May, 1976.

Patients were randomized to receive (a) Glaxo BCG, $10^7$ viable organisms following surgery, then 8 weeks' Isoniazid 300 mg daily starting 2 weeks later; or (b) Placebo (Lactose) tablets for 8 weeks commencing 2½ weeks after surgery. Ninety-two patients have entered the study, 77 having non-anaplastic cancer. Six of the 38 BCG-treated and 9 of the 39 control non-anaplastic cancer patients have died, giving cumulative 18-month survival rates of 77% and 63% respectively. This difference is not significant. Two of the 22 Stage I non-anaplastic BCG patients have died compared with 7 of 23 controls, and this trend reaches significance ($\chi^2 = 4{\cdot}195$, $P < 0{\cdot}05$, Log rank test). Approximately half of the patients with anaplastic ca. have died, irrespective of staging or treatment. Half of the BCG-treated patients had a febrile reaction and this was independent of heaf-test response; other complications were similar in both groups.

The limited benefit from Glaxo BCG may be related to the lower incidence and duration of febrile response compared to that reported with Tice BCG, but since 5 of the 11 BCG-treated patients to die had been febrile other factors may be important. These data suggest that intra-pleural Glaxo BCG in the dosage used may have enhanced non-anaplastic cancer patients' survival.

### PART II

### POSTER EXHIBITS

### IDENTIFICATION OF THE PROXIMATE FORM OF THE CARCINOGEN 15,16-DIHYDRO - 11 - METHYLCYCLOPENTA - [A]PHENANTHREN-17-ONE.

M. M. COOMBS, T. S. BHATT and A. M. KISSONERGHIS, *Chemistry Department, Imperial Cancer Research Fund, London.*

After microsomal activation, the [$^{14}$C]-carcinogen(I) bound to DNA *in vitro*. Enzymatic hydrolysis of the DNA, followed by chromatography on Sephadex LH 20 columns, yielded 2 adduct peaks, A and B, which eluted after the unchanged nucleosides. Isolation of DNA from mouse skin treated *in vivo* with (I), and analysis as before gave only one adduct peak, chromatographically identical with B. Individual metabolites of (I), also separated on LH 20 columns, were incubated with the microsomal activating system and DNA, which was then recovered and analysed. Adduct B arose from a single metabolite with the polarity of a diol, identified by chemical reactions employing UV spectroscopy as the 3,4-dihydro-3,4-dihydroxy derivative of (I). This is therefore the proximate form of this carcinogen.

Adduct B had a UV spectrum similar to that of 1,2,3,4-tetrahydro-(I). The aryl hydrocarbon hydroxylase inhibitor 7,8-benzoflavone delayed skin tumour production with (I) (Coombs *et al.*, 1975, *Cancer Res.*, **35**, 305). Moreover, in another experiment the mean latent period was decreased from 35 to 22 weeks by administrations of (I) together with the epoxide-hydrase inhibitor 1,1,1-trichloropropane oxide. Both these observations indicate that the reactive metabolite is an aryl oxide. It is therefore suggested that the ultimate form of this carcinogen is a 3,4-diol-1,2-oxide of (I), analogous to the biologically active diolepoxide of benz[a]pyrene.

### CHANGES IN SURFACE MORPHOLOGY RELATED TO ETHYL-NITROSOUREA-INDUCED MALIGNANT TRANSFORMATION OF CULTURED RAT BRAIN CELLS.

J. P. ROSCOE, D. P. WINSLOW and P. ROWLES, *School of Pathology, Middlesex Hospital Medical School, London.*

Cultures of cells clonally derived from either normal adult rat brain or a culture of a mixed glioma were studied by scanning electron microscopy. The two normal lines, ARBO C9 and ARBO C11, were found to display very little surface activity. The cells were large, extremely flat with prominent nuclei and had few microvilli or ruffles. In contrast, the two tumour lines A15A5 and A15A10 displayed a striking amount of surface activity which greatly exceeded that shown by the normal cells grown and fixed under identical conditions. These cells were highly condensed and possessed large numbers of blebs, filopodia and pleomorphic microvilli. Cells derived from rat brains transplacentally exposed to either ethyl-nitrosourea (ENU) or citrate buffer and cultured at 111–112 days after injection were also studied. The results here support our above findings; showing similar differences in the surface activity of control and tumourigenic lines. Preliminary results from one line suggest that cells exposed to ENU but which had not yet shown transformed properties such as growth in agar, tumourigenicity or high fibrinolytic activity displayed an intermediate morphology between control and tumourigenic lines.

### EPIDEMIOLOGICAL STUDY OF THE MORTALITY OF BRITISH CHEMISTS.

C. E. SEARLE and J. A. H. WATERHOUSE, *Univ. of Birmingham Medical School, Birmingham* and B. A. HENMAN, D. BARTLETT and S. MCCOMBIE, *Royal Institute of Chemistry, London.*

Chemists are exposed to a wide range of chemicals during their training and work, but few epidemiological studies of their health have been carried out. Members of the American Chemical Society were reported to have an increased death rate from malignancy relative to other professional workers (Li *et al.*, 1969, *J. Natl. Cancer Inst.*, **43**, 1159) the most marked increases being in lymphomas and pancreatic tumours. Respiratory disease and liver cirrhosis were less common in the chemists. A smaller but more complete study of Swedish chemists also noted an increase in deaths from lymphomas (Olin, 1976, *Lancet*, ii, 916).

A study of British chemists has now been initiated. Death certificates were obtained for 1332 members of the Royal Institute of Chemistry who died in 1965–75 inclusive. Deaths of 291 were from neoplasms, representing a higher-than-expected proportion of all causes of death in the age-range 36–50. Though analysis of deaths is in an early stage, it appears that the incidence of lymphomas may be elevated in RIC members also.

Relative to the general population there is a high colon/rectum cancer ratio in the chemists. However, this is also seen in medical practitioners (Doll and Peto, 1976, *Br. Med. J.*, ii, 1525) suggesting that it is associated with socioeconomic status rather than chemical exposure.

Good records of the occupational histories of many RIC members offer possibilities of linking mortality with chemical exposure. It is hoped to extend the retrospective study and to initiate a prospective one.

**LUNG-CANCER RISKS IN THE SMOKING OF DIFFERENT TOBACCOS.** T. E. Betts and L. A. Elson, *Research Laboratories, Brompton Hospital, London.*

Reliance solely on mouse-skin carcinogenicity tests is not satisfactory in assessing the cancer risks in smoking different tobaccos.

Although no frankly malignant transformations have been seen, exposure of rats to the smoke of different tobaccos can result in pathological effects on the lungs and bronchi, the extent of which varies with the type of tobacco smoked.

Groups of rats, reared under minimal disease conditions, were exposed to the smoke of (A) Flue-cured, (B) Air-cured and (C) Cigar tobacco. After 6 weeks' exposure, quantitative measurements were made, post mortem, on formalin-fixed lung and bronchial tissue. The increase in serum alpha-1-acute-phase protein (Darcy) was also measured.

| Tobacco | Lung wt. (g) | Goblet cells | Epith. ht ($\mu$m) | Active-phase serum protein |
|---|---|---|---|---|
| Flue-cured | 9·14±0·51 | 17·8±7·7 | 39·73±22·0 | 2·01±1·03 |
| Air-cured | 9·21±0·88 | 12·7±8·3 | 13·76± 7·69 | 1·42±1·47 |
| Cigar | 6·57±0·46 | 2·4±1·3 | 12·55± 2·34 | 0·13±0·05 |
| Control | 5·76±0·59 | 0·4±0·3 | 12·55± 2·52 | 0·06±0·01 |

Under these conditions, where inhalation is obligatory, the smoke of Flue-cured and Air-cured tobaccos produces more serious pathological changes than does the smoke of Cigar tobacco.

Possibly some factor not distinguished in the mouse-skin painting test is removed or modified in the cigar-tobacco fermentation process.

**$\alpha$-HYDROXYLATION OF HETEROALICYCLIC TRIAZENES.** A. Gescher, J. A. Hickman and M. F. G. Stevens, *Cancer Chemotherapy Group, Dept. Pharmacy, Univ. of Aston in Birmingham.*

Although heteroalicyclic triazenes are, in general, inactive as antitumour agents, their chemical properties show similarities to those of the antitumour aryldimethyltriazenes and the carcinogenic heteroalicyclic nitrosamines (Gescher *et al.*, 1977, *J. Chem. Soc. Perkin Trans.*, **I**, pp. 107 and 2078). A study of the chemical and metabolic oxidation of such compounds can shed light on the bio-activation processes for all these structural types.

Oxidation of 4-(4-chlorophenylazo)morpholine (1) with potassium permanganate in aqueous acetone, or by the Udenfriend method, or by incubation with a rat-liver homogenate in the presence of an NADPH-generating system, afforded the $\alpha$-oxidized triazene 4-(4-chlorophenylazo)morpholin-3-ol (2) together with

(1) R = H; (2) R = OH

(3)

traces of 1,3-bis-(4-chlorophenyl)triazene and 4-chloroaniline. Attempted acetylation of (2) with acetyl chloride in pyridine led to an elimination reaction and the formation of the dehydromorpholine (3). Although $\alpha$-oxidation is assumed to be a crucial step in the bio-activation of triazenes, this is the first report of the isolation and characterization of an $\alpha$-hydroxylated derivative from the metabolic transformation of a triazene.

**TWO APPROACHES TO SELECTIVE REVERSAL OF METHOTREXATE TOXICITY.** P. J. Dady, G. A. Taylor, A. H. Calvert and K. R. Harrap, *Dept. Biochemical Pharmacology, Institute of Cancer Research, Sutton, Surrey.*

We have shown that L1210-tumour-bearing DBA2/C57 BL F1 hybrid mice may be rescued from toxic doses of methotrexate by a combination of inosine, thymidine and allopurinol. Animals "rescued" with this purine and pyrimidine combination survived longer than those "rescued" with folinic acid, indicating enhanced antitumour activity.

Tissue culture studies have indicated that 5-methyl tetrahydrofolate may have theoretical advantages over folinic acid as a rescue agent. We present some experiments using high-dose methotrexate in tumour-bearing animals, which confirm that "rescue" with 5-methyl tetrahydrofolate can be more selective than folinic-acid rescue.

**THE PHARMACOKINETICS OF MISONIDAZOLE IN NORMAL AND TUMOUR-BEARING DOGS.** R. A. S. WHITE*, P. WORKMAN, L. N. OWEN* and N. M. BLEEHEN, *Clinical Oncology and Radiotherapeutics Unit, Hills Road and *Dept. of Clinical Veterinary Medicine, Madingley Road, Cambridge.*

The hypoxic cell sensitizer misonidazole (Ro 07-0582) has a much shorter half-life in the mouse and rat than in man. We therefore studied the pharmacokinetics of the drug in the dog to evaluate this species as a model for man.

Levels of misonidazole and its O-demethylated metabolite Ro 05-9963 in plasma and tissues were estimated by high-performance liquid chromatography (Workman *et al.*, *J. Chromatog.*, in press). In normal dogs the bioavailability of misonidazole was similar after i.v. and oral administration. After oral dosage, peak plasma concentrations occurred between 2 and 6 h. The half-life of the drug (4–9 h) was independent of dose and route of administration and was similar to that in man (4–12 h) (Wiltshire *et al.*, unpub.). The apparent volume of distribution of misonidazole indicated uniform distribution in body water. Similar levels of Ro 05-9963 after various doses of misonidazole indicated possible saturation of demethylating enzyme(s).

In 3 dogs bearing spontaneous tumours, given 150 mg/kg i.v., concentrations in normal tissues, including brain, were similar to the corresponding plasma concentrations. An exception was the liver, which was $<10\%$. Tumour levels reached 80–90% of the corresponding plasma concentrations.

The similarity of these data to those in man indicate that the dog may be a good model for the study of misonidazole as a hypoxic cell sensitizer.

***IN VITRO* METABOLISM OF CYTOSINE ARABINOSIDE IN NORMAL AND TUMOUR BREAST TISSUE.** A. BAXTER, L. M. CURRIE and J. P. DURHAM, *Dept. of Clinical Oncology, Univ. of Glasgow, and* D. C. SMITH, *Div. of Surgery, Victoria Infirmary, Glasgow.*

Significant levels of deoxycytidine kinase, the enzyme which activates cytosine arabinoside (Ara-C) have been measured in human breast tumours and normal breast tissue. A mean activity of 0·5 u/mg protein was found in 21 tumours and of 0·52 u/mg protein in 9 controls. Concomitant measurement of cytidine deaminase, the enzyme which inactivates Ara-C, yields a mean tumour value of 1·6 u/mg protein and a mean normal value of 2·3 u/mg protein. These levels of kinase activity and the ratios of kinase to deaminase activity compare very favourably with data for other human normal and neoplastic tissues (Ho, 1973, *Cancer Res.*, **33**, 2816).

Kinetic studies on the interaction of Ara-C with these enzymes have been carried out on paired normal and tumour tissues from 6 patients. In tumour tissue the kinase displays an 11-fold higher affinity (as determined by $K_m$ measurements) than the deaminase for Ara-C, while in normal tissue the affinity is 6-fold higher. When these data are related to the ratios of the enzyme activities, the net effect should be activation of the drug, and suggests a possible use of Ara-C in the treatment of carcinoma of the breast. Moreover, these data suggest that activation of the drug will be 3 times higher in tumour tissue than in control tissue, leading to decreased toxicity in normal breast tissue.

**THE EFFECTS OF HYDROXYUREA (HU) AND CYTOSINE ARABINOSIDE (ARA-C) ON JEJUNAL CRYPTS AND ACCESSORY SEX ORGANS (ASO) IN THE MOUSE.** M. R. ALISON and N. A. WRIGHT, *Harkness Laboratories, Radcliffe Infirmary, Oxford.*

It has been reported that DNA-synthesizing cells of cell renewal systems are more susceptible to the necrotizing effects of HU than those in stimulated tissues (Farber and Baserga, 1969, *Cancer Res.*, **29**, 136). ASO epithelial cells are quiescent, but enter DNA synthesis after a period of androgen deprivation (3 days) and testosterone (T) injection. A continuous thymidine-labelling technique revealed that the fraction of proliferating (P) cells in the coagulating gland (CG) was 30%. Mice were injected with either Ara-C (400 mg/kg body wt.) or HU (1600 mg/kg body wt.) after 50 h T stimulation. Some mice received a second injection at a time of maximum labelling index during the recovery phase, 10 and 14 h later in the Ara-C and HU-treated groups respectively. Ara-C and HU were equally damaging upon crypt cells, 38% of cells being degenerate after 2–4 h. Crypt column height was reduced from $23·4\pm2·2$(s.d.)$-28·1 \pm 3$ cells (range during T stimulation) to $16·5\pm 1·6$ cells 8 h after Ara-C and $18·2\pm1·5$ cells 8 h after HU. In the ASO, DNA synthesis was similarly inhibited for 6–8 h. Fewer necrotic cells were observed than expected from labelling indices, but the greater cytocidal effect of Ara-C was associated with the recruitment of non-cycling (Q) cells into the P compartment, a phenomenon of significance in tumour growth (Gelfant, 1977, *Cancer Res.*, **37**, 3845).

### EFFECTS OF MULTIPLE HUMAN-EQUIVALENT I.V. DOSES OF *CORYNEBACTERIUM PARVUM* IN THE MOUSE.

H. D. MITCHESON, T. E. SADLER and J. E. CASTRO, *Urology and Transplantation Unit, Royal Postgraduate Medical School, Hammersmith Hospital, London.*

The aim of this study was to investigate the effects of multiple human-equivalent i.v. doses of *C. parvum* in the mouse, and to compare these with the response to a single high dose of vaccine. The whole-body, liver, spleen and thymus weights, and antibody titre against *C. parvum* were determined.

*C. parvum* was injected either weekly as a low dose (70 μg) or as a single high dose (466 μg). Both treatments had similar effects on total-body, liver, spleen and thymus weights. However multiple low doses caused a higher antibody titre against *C. parvum*, 25 $\log_2$ after 4 injections compared with 8 $\log_2$ after a single high dose.

The effect of these treatments on the growth of primary Lewis tumour and its metastases was studied. Multiple low-dose *C. parvum* treatment was given both before or after tumour inoculation. These results will be discussed.

### UNCONJUGATED DIHYDROTESTOSTERONE AND TESTOSTERONE LEVELS IN SERUM OF PATIENTS WITH CARCINOMA OF THE PROSTATE.

R. GHANADIAN, C. M. PUAH and E. P. N. O'DONOGHUE, *Prostate Research Laboratory, Royal Postgraduate Medical School and Institute of Urology, London.*

In an extensive study, the effect of antiandrogens and oestrogen treatment on the level of circulating dihydrotestosterone and testosterone was investigated. Patients with histologically proven prostatic carcinoma are treated by one of the following treatment modalities according to a prospective protocol: stilboestrol 1 mg t.d.s., cyproterone acetate 100 mg b.d., bilateral orchidectomy or no immediate treatment. Plasma dihydrotestosterone and testosterone have been monitored using a sensitive and reliable radioimmunoassay developed in our laboratory (Ghanadian *et al.*, 1975, *Steroids*, **25**, 753).

There was a significant correlation between pre-treatment plasma dihydrotestosterone and testosterone levels ($P < 0{\cdot}05$). However, after hormonal treatment, such a relationship no longer existed ($P > 0{\cdot}1$) although there was significant suppression of both dihydrotestosterone and testosterone levels. These data suggest a differential effect on these 2 androgens. Suppression of plasma testosterone was significantly less ($P < 0{\cdot}05$) in the cyproterone acetate group than in the stilboestrol or orchidectomy groups.

### DISTRIBUTION OF [$^{99m}$TC]TECHNETIUM-LABELLED LIPOSOMES IN PATIENTS WITH CANCER.

V. J. RICHARDSON*, K. JEYASINGH†, R. F. JEWKES†, S. B. KAYE‡, E. S. NEWLANDS‡, B. E. RYMAN* and M. H. N. TATTERSALL‡, *Departments of *Biochemistry, †Nuclear Medicine and ‡Medical Oncology, Charing Cross Hospital and Charing Cross Hospital Medical School, London.*

Liposomes (phospholipid vesicles) have been proposed as carriers of antitumour drugs in order to alter the blood clearance, tumour and marrow localization of the drugs and thereby increase their therapeutic value. γ-Camera studies have shown that $^{99m}$Tc (technetium) label attached to small anionic liposomes concentrates in the Walker 256 carcinoma model (Richardson *et al.*, 1977, *Biochem. Soc. Trans.*, **5**, 290). We have now studied the distribution of $^{99m}$Tc-labelled liposomes in 14 patients with advanced cancer and one patient with polycythaemia rubra vera (PRV). Pharmaceutical preparations of liposomes, 20 or 300 mg of lipid (7:2:1 molar ratio phosphatidylcholine:cholesterol:phosphatidic acid) labelled with ~20 mCi of $^{99m}$Tc, were injected i.v. Blood clearance and urine excretion of the label were measured over 24 h, and the tissue distribution at 24 h was observed using a whole-body scanner.

In all cases, excluding the patient with PRV, the major site of uptake of the label was the liver and spleen. In the patient with PRV, liver and spleen uptake were greatly reduced and the majority of the radioactivity was found in sites corresponding to the marrow.

With the possible exception of one patient with a hepatoma, there was no evidence of tumour localization of radioactivity. The interesting results in the patient with PRV may reflect the altered marrow turnover which occurs in this condition. Future plans include a study on larger numbers of patients using liposomes of different sizes and lipid compositions.

### DEVELOPMENT OF A RADIOIMMUNOASSAY FOR A RAT SARCOMA-ASSOCIATED SPECIFIC ANTIGEN.

V. E. PRESTON and M. R. PRICE, *Cancer Research Campaign Laboratories, Univ. of Nottingham.*

A partially purified, tumour-specific antigen was isolated from soluble 3 M-KCl extracts of a transplanted, 3-methylcholanthrene-induced rat sarcoma Mc7 by immunoadsorption to syngeneic immune IgG antibodies immobilized by covalent linkage to Sepharose 4B (Preston and Price, 1977, *Biochem. Soc. Trans.*, **5**, 123). This antigen preparation was used to prepare a rabbit antiserum, which, after absorption with normal tissue extracts and sarcoma Mc40A cells, displayed unique specificity for sarcoma Mc7 cells in indirect membrane immunofluorescence tests. Radioiodinated, immunoadsorbent-purified sarcoma Mc7 antigen was reacted with this antiserum, and labelled immune complexes were precipitated by addition of an excess of heat-killed, formaldehyde-fixed *Staphylococcus aureus* organisms (which express the Fc-binding component, Protein A). A radioimmunoassay for sarcoma Mc7 antigen was developed by measuring the competitive inhibition of this precipitation reaction by the addition of unlabelled antigen in crude soluble extracts. At the present stage of development, the assay retains the capacity to distinguish between extracts of different chemically induced tumours and has been successfully used to monitor the fractionation of soluble sarcoma Mc7 extracts.

**ENHANCEMENT OF LYMPHOCYTE-MEDIATED CYTOTOXICITY AFTER TUMOUR RESECTION IN PATIENTS WITH COLORECTAL CANCER.** P. G. GILL, C. WALLER and I. McLELLAN, *Nuffield Departments of Medicine and Surgery, Radcliffe Infirmary, Oxford.*

Lymphocyte numbers and function, in 37 patients with colorectal cancer, were compared with those in 23 healthy control subjects of the same age range. No significant difference in lymphocyte count, numbers of cells forming E rosettes or bearing surface immunoglobulin, cell-mediated cytotoxicity or mitotic response to PHA were found. Six to twelve months after tumour resection, patients showed a rise in antibody and PHA-induced cytotoxicity to levels significantly higher than those found in age-matched controls. This rise occurred irrespective of whether patients had received adjuvant immunotherapy with *C. parvum*.

**CYTOTOXIC AND PHAGOCYTIC ACTIVITY OF HUMAN TUMOUR INFILTRATING MACROPHAGES.** B. M. VOSE, *Paterson Laboratories, Christie Hospital and Holt Radium Institute, Manchester.*

Adherent cells separated from human tumour specimens were tested for cytotoxic potential against antibody-coated human red cells and against autologous tumour cells in 18 h $^{51}$Cr-release assay. These cells represented 0·2–8·5% of the cells plated, and had the morphological appearance of macrophages. 64–88% showed uptake of 1·1 $\mu$m diameter latex particles and 77–94% pinocytosed neutral-red solution. Lysis of antibody coated human erythrocytes was determined in 14/15 cases examined with 2 of 20 positive without antiserum. Significantly increased $^{51}$Cr release over background was recorded in 19/27 tests against autologous tumour cells using the monolayers as effectors. This latter cytotoxicity was manifested in the presence of autologous and allogeneic sera and was also evident against allogeneic tumour targets. Reactivity was less frequently determined against targets from uninvolved lung tissue from patients with pulmonary neoplasia. Macrophage cytotoxicity was often associated with T-cell-mediated anti-tumour cytotoxic activity in the blood lymphocytes.

***IN VIVO* FATE OF $^{125}$I-LABELLED TUMOUR CELLS IN MICE GIVEN ANTIBODY AND ARA-C.** P. D. E. JONES, F. GOTCH, G. F. ROWLAND and G. J. O'NEILL, *G. D. Searle Research Labs., High Wycombe, Bucks., and Nuffield Dept. Clinical Medicine, Radcliffe Infirmary, Oxford.*

Administration of rabbit anti-EL4-tumour sera (RaEL4) in conjunction with cytosine arabinoside (Ara-C) was found to prolong survival of C57BL/6 mice bearing the syngeneic EL4 tumour. A synergistic protective effect against tumour development with antibody and drug may be obtained by a multiple-treatment regimen (Davies *et al.*, 1974, *Br. J. Cancer*, **30**, 305). Using $^{125}$I-deoxyuridine-labelled tumour cells and daily whole body measurement of retained radioactivity of individual mice, it is possible to follow *in vivo* the fate of the tumour cells after therapy (Carlson and Terres, 1976, *J. Immunol.*, **117**, 822). Preliminary studies show that in an allogeneic situation, RaEL4 (0·1–0·2 ml) or Ara-C (1–2 mg) given i.p. or i.v. to CBA mice 24 h after i.p. injection of $10^7$ labelled EL4 cells, results in the rapid killing of these cells, whereas rabbit antisera against the C57BL/6 B16 tumour has no effect. The rate of cell death after RaEL4 and Ara-C in conjunction was greater than that obtained by either treatment alone.

This technique allows the analysis and rapid screening of tumour therapy *in vivo* with antibody and drug, using the mouse as a host to syn-, allo- and possibly xeno-geneic tumours.

**DNA REPAIR SYNTHESIS, FACT OR ARTIFACT?** B. W. FOX, *Paterson Laboratories, Christie Hospital and Holt Radium Institute, Manchester.*

The measurement of DNA repair synthesis at different times after treatment, is essential to an understanding of the relative sensitivity of tumour lines to DNA-damaging agents. The density-labelling centrifugation procedure has been widely used for measuring DNA repair synthesis in the first few hours after treatment.

However, during investigations over a longer time, we (Nikaido and Fox, 1976, *Chem. Biol. Interact.*, **14**, 47) observed a pattern of "repair synthesis" occurring in untreated cells. This phenomenon has been studied in greater detail, using a benzoylated-naphthoylated (BND)-column procedure, which does not require a density label. The "repair synthesis" initially observed was found to be due, in part, to a differential exclusion of DNA precursors by the cell, possibly at the cell membrane. However, a further artifact has been uncovered, dependent on the centrifugation field applied, which has important implications in the interpretation of DNA repair synthesis obtained by any of the current procedures.

**INCREASE IN CHROMOSOMAL RADIOSENSITIVITY OF CHINESE HAMSTER CELLS WITH *IN VITRO* PASSAGE.** C. Y. LYONS, D. SCOTT and J. R. CONNELL, *Paterson Laboratories, Christie Hospital and Holt Radium Institute, Manchester.*

Cells from cancer-prone individuals with a trisomic chromosome constitution (e.g. Downs syndrome) have an elevated sensitivity to X-ray-induced chromosome aberrations (Sasaki *et al.*, 1970, *Mutation Res.*, **10**, 617). The sensitivity of a trisomic Chinese hamster cell line (passage 40) to X-ray-induced chromosome damage was found to be twice that of the diploid line (passage 16) from which it arose spontaneously. A line carrying a simple inversion (passage 9) was of similar sensitivity to the diploid. Further experiments suggest, however, that the difference in radiosensitivity is not related to the karyotypic differences, but to age in culture, since the diploid cells were found to increase in sensitivity between passage 16 and 47. Cells may lose their repair capacities with increasing *in vitro* culturing.

**REGRESSION PATTERN ANALYSIS FOR THE CELLULAR CHEMOSENSITIVITY OF HUMAN MULTIPLE MYELOMA.** G. F. BRUNTON, T. E. WHELDON and A. H. W. NIAS, *Glasgow Institute of Radiotherapeutics and Oncology, Western Infirmary, Glasgow.*

The kinetics of growth and regression during non-cycle-specific chemotherapy of human IgG multiple myeloma using melphalan, may be interpreted using a mathematical model which allows identification of the parameter of cellular chemosensitivity, $D_0$, for an individual patient.

Knowledge of these parameters may be used to predict tumour response, not only to the continued application of the treatment schedule chosen originally, but also to any other schedule using the same agent.

For 11 published cases (Sullivan and Salmon, 1972, *J. Clin. Invest.*, **51**, 1697) the mean $D_0$ was found to be 77·6 mg, with a range of 25·4–198 mg, reflecting an intrinsic variability in chemosensitivity to melphalan at the cellular level, and emphasizing the necessity of individually orientated schedule design. Computer simulation of individual response to alternative schedules, using $D_0$ values estimated early in an arbitrarily chosen schedule, may assist in rationalizing the therapy of this disease.

**FLUOROCHROMASIA AND FLUORESCENCE POLARIZATION IN LYMPHOID CELLS.** A. W. PREECE, P. A. LIGHT and P. BALDING, *Oncology Research Laboratories, Radiotherapy Centre, Bristol.*

The SCM test for malignant disease utilizes the change in polarization value of fluorescein to demonstrate lymphocyte response to PHA or tumour basic protein (TBP).

The kinetics of fluorochromasia in a range of lymphoid cells, including human lymphocytes, is such that there is a rapid decrease in polarization over 10–20 minutes, related to the rate of accumulation of intracellular fluorescein. The presence of PHA or TBP does not appear to alter the polarization value, but may have an effect on the rate of hydrolysis and accumulation of fluorescein.

Data for a group of healthy controls and cancer patients show no significant difference in the curves of P value against time.

**CELL PARTITION: OPTIMUM SYSTEMS FOR DETECTION OF SURFACE CHANGES.** J. P. DICKINSON and C. M. BALLARD, *Y.C.R.C. Laboratories, Univ. Dept. of Radiotherapy, Cookridge Hospital, Leeds.*

Following on the partial success of cell partition as an attractive alternative to the MEM test for lymphocyte sensitization (Smith and Dickinson, 1976, *Lancet*, ii) detailed study of the partition phenomenon has been made, in order to optimize conditions for routine measurement of cell-surface-charge changes.

For a variety of cells modified in surface charge by digestion with neuraminidase or trypsin, or treated with maleic anhydride, changes in partition coefficient correlate strongly with changes in electrophoretic mobility: theoretically each is dependent on the surface-charge density.

Optimum phase systems for observation of increases or decreases in surface charge of human lymphocytes, leucocytes and erythrocytes, guinea-pig macrophages and tanned sheep erythrocytes have been defined in terms of polymer and electrolyte concentration.

As a corollary, study of the transfacial potential difference ($\Delta V$) shows that, as predicted by theory, ln (partition coefficient) $\propto \Delta V$. Increasing PEG concentration increases $\Delta V$; increasing NaCl beyond 30 mM reduces $\Delta V$ virtually to zero. Polymer-alone systems exhibit a definite $\Delta V$ which is greatly increased by small concentrations (< 10 mM) but gradually discharged by increasing concentrations, of phosphate buffer.

**ORGAN DISTRIBUTION OF NATURAL CYTOTOXICITY IN THE RAT.** M. R, POTTER and M. MOORE, *Immunology Division. Paterson Laboratories, Christie Hospital and Holt Radium Institute, Manchester.*

The natural (spontaneous) cytotoxicity (NC) of cell populations from different lymphoid organs of the rat were examined, using a human myeloid cell line (K562) and a rat fibrosarcoma cell line (Mc40) as target cells. Rat blood and spleen lymphoid-cell populations gave high cytotoxicity against K562, while lymphnode cells and marrow cells gave low levels of cytotoxicity, and thymus cells virtually no activity. Addition of thymus or lymphnode cells to spleen effector cells did not suppress the high cytotoxicity of spleen cells. A similar organ distribution of reactivity was seen against Mc40 cells, but the levels of cytotoxicity were much lower than for K562.

A strain difference was monitored in the levels of natural cytotoxicity and cell populations from inbred Wistar rats consistently gave higher activity on a cell-to-cell basis than the corresponding population from PVG/c rats. Natural cytotoxicity was not removed when spleen-cell populations were depleted of cells adhering to nylon-fibre columns or plastic surfaces, or depleted of cells ingesting carbonyl iron. In agreement with other studies using human and animal lymphoid cells, the natural killer cell in this system was found to be non-adherent and non-phagocytic and its distribution did not correspond to the established organ distribution of T or B lymphocytes.

**NATURAL CELL-MEDIATED IMMUNITY: REACTIVITY AGAINST MYCOPLASMA-INFECTED CELLS.** C. G. BROOKS*, R. C. REES† and R. H. LEACH‡, **Cancer Research Campaign Laboratories, Univ. of Nottingham, †Dept. of Virology, Univ. of Sheffield Medical School, ‡Mycoplasma Reference Laboratory, Norwich.*

We have found that infection of tumour cells with certain mycoplasma species causes a large increase in their susceptibility to the cytotoxic effects of natural killer (NK) cells. Of 3 mycoplasma species tested, two have this property—*M. arginini* and *M. hyorhinis*—while a third, *M. orale*, has been found to be negative. The NK-cell reactivity against mycoplasma-infected tumour cells crosses both strain and species barriers. For example, lymphnode cells (LNC) from syngeneic rats, allogeneic rats, mice and rabbits are reactive against mycoplasma-infected rat tumour cells. Natural immunization by mycoplasma or bacterial infection is not responsible for this reactivity, as LNC from mycoplasma-free and germ-free rats were as reactive as LNC from conventionally housed rats. Furthermore, spleen cells from nude mice were strongly reactive, indicating that the effector cell is non-T. These findings suggest that the elevated NK-cell reactivity often observed against *in vitro*-grown target cells may sometimes be caused by mycoplasma infection, and indicates that mycoplasma infection somehow increases target-cell sensitivity to NK cells.

**RESPONSE OF MURINE THYMUS CELLS TO DEVELOPMENT OF A TRANSPLANTABLE TUMOUR.** P. A. LIGHT, A. W. PREECE and R. ASPINALL, *Oncology Research Laboratories, Radiotherapy Centre, Bristol.*

Alterations in the properties of murine thymocytes during development of a transplantable tumour (NK/Lymphoma) have been detected by measurements of migration inhibition of thymocytes. It was found that marked inhibition of migration from capillary tubes of thymocytes from tumour-bearing animals occurred when autologous tumour-cell fragments were present in the medium surrounding the capillary tubes. Such migration inhibition was observed with thymocytes from animals in whom the tumour had been transplanted only 6 h earlier. By 24 h after tumour transplantation, inhibition had risen to 65%, and by Day 5 was 95%. Similar effects were observed when syngeneic lymphoma-cell fragments were tested against thymocytes from tumour bearers, although the actual inhibition values were slightly lower. Thymocytes from normal animals were tested in the same migration assay system against membrane fragments prepared from lymphoma cells from various stages of growth. In no instance was any migration inhibition observed in these controls.

**TRANSPLANTATION OF CANINE LYMPHOSARCOMA TO NUDE MICE.** D. R. MORGAN, D. E. ONIONS and L. N. OWEN, *Dept. of Clinical Veterinary Medicine, Univ. of Cambridge.*

The successful transplantation of canine osteosarcoma, mammary carcinoma and melanoma to nude mice has been previously reported (Oughton and Owen, 1974, *Res. Vet. Sci.*, **17**, 414; Morgan, 1977, *Br. J. Cancer*, **36**, 427). Attempts to transplant both human and canine lymphosarcoma have rarely been successful, although bovine lymphosarcoma has been transplanted to nude mice following whole-body X-irradiation (Irvin *et al.*, 1977, *Res. Vet. Sci.*, **22**, 53). In our experiments cell suspensions of lymph node from each of 11 dogs histologically diagnosed as multicentric lymphosarcoma were injected into outbred nude mice. In one case the recipient mice were pretreated with 300–500 rad of whole-body X-irradiation. Tumours did not grow in non-irradiated mice, but all X-irradiated mice developed tumours within 4 weeks. The tumour was readily passaged *in vivo* and from the second passage tumours grew in non-irradiated mice as rapidly as in mice given whole-body X-irradiation. S.c. tumour growth was diffuse and infiltrating, and injection of $5 \times 10^7$ cells produced 5 g of lymphosarcoma tissue within 6 weeks. The tumour was composed of closely packed lymphoid cells with some lymphoblastic types, and mitotic figures were common. Metastases were frequent, affecting the axillary lymph nodes, lungs, kidney and heart.

### THE ADHESION OF CELLS TO 3-DIMENSIONAL COLLAGEN LATTICES.

S. L. Schor* and T. D. Allen†, **C.R.C. Department of Medical Oncology*, *†Dept. of Ultrastructure, Christie Hospital, Manchester.*

We have measured the kinetics of cell adhesion to both plastic tissue-culture dishes and 3-dimensional collagen lattices. The cells examined in this initial study (24 types) include normal, transformed and tumour cells of both epithelial and mesenchymal origin. Cells were removed from the surfaces of plastic tissue-culture dishes by a 20 min incubation in EGTA (2 mM) and trypsin (0·05%) and a cell suspension ($\sim 1 \times 10^5$ cells/ml) prepared in growth medium supplemented with 10% foetal calf serum. 1 ml of cell suspension was then added to 35 mm plastic dishes, with or without a collagen substratum, and the kinetics of cell adhesion determined by counting the number of cells recovered in the medium after gentle washing of the dishes at various times. The cells examined in this manner fall into 3 distinct groups as defined by their relative kinetics of adhesion to plastic and collagen (the data are expressed as % of initial cells adherent after 2 h): the cells may (a) adhere at an equal rate and extent to both plastic and collagen ($>80\%$), (b) adhere faster to plastic ($>80\%$) than to collagen ($<20\%$), or (c) adhere faster to collagen ($>80\%$) than to plastic ($<20\%$).

Differences in overall cell morphology, as well as surface details, were observed with the SEM between cells grown on plastic and collagen. These morphological and kinetic data form the basis for ongoing work designed to examine the mechanism of cell adhesion to collagen, especially possible differences which may exist between cell types.

### GLYCOPROTEINS AND GLYCOSAMINOGLYCANS OF CULTURED HUMAN SKIN FIBROBLASTS.

N. Gasiunas, J. T. Gallagher and S. Schor, *C.R.C. Department of Medical Oncology, Christie Hospital, Manchester.*

Monolayers of adult human skin fibroblasts grown on plastic surfaces or collagen gels were incubated for 48 h with $^3$H-glucosamine and $Na_2{}^{35}SO_4$. A trypsin (0·05% w/v)/EGTA (2 mM) extract of cells on plastic, presumed to contain cell-surface and intercellular macromolecules, was analysed by gradient elution from DEAE-cellulose. Four $^3$H-labelled peaks were resolved, but only two of these were labelled $^{35}$S-sulphate. The non-sulphated components were hyaluronic acid and acidic glycoproteins; the doubly-labelled peaks were heparin sulphate (HS)/(main component) and chondroitin sulphate-B (CS-B).

Cells grown on collagen gels were not detached by trypsin/EGTA, but this solution mobilized labelled materials qualitatively similarly to those on plastic, but with a significant shift in the sulphated glycosaminoglycans to predominantly CS-B. The gel was then dissolved with collagenase, which released adhering cells and cells which had migrated into the gel network. The collagenate was also enriched in CS-B, and in addition showed a greatly elevated $^3$H-label in a neutral glycoprotein. Thus, modification in the pattern of "matrix" synthesis by cells *in vitro* can be mediated through an interaction between cells and their environment.

### SYNTHESIS AND RELEASE OF SPECIFIC PROTEIN PRODUCTS BY HUMAN MAMMARY TUMOUR CELLS *IN VITRO*.

R. J. Grieve, K. L. Woods, D. H. Cove, S. C. H. Smith, J. Leonard* and A. Howell, *Depts. of Medicine and Experimental Pathology, Univ. of Birmingham, and *The Birmingham and Midland Hospital for Women, Birmingham* 11.

We have found that lactalbumin, $\alpha$ subunit of glycoprotein hormones and carcinoembryonic antigen are elevated in the sera of 12%, 8% and 16% of patients with operable breast cancer (Stages I and II). The same proteins have been identified in the cytosols of 35%, 42% and 70% of primary mammary carcinomas. If these proteins are to prove of value as tumour markers, information is needed on the control of their synthesis and release. All 3 proteins are produced by the human mammary carcinoma line MCF-7, and we have studied their regulation *in vitro*. Sodium butyrate increases $\alpha$ subunit production by a factor of 264 and lactalbumin production by a factor of 40. Synthesis and release of $\alpha$ subunit is increased by phosphodiesterase inhibitors, but unaffected by LHRH and TRH.

**COHORT STUDY OF OVARIAN CANCER CASES.** D. J. HOLE and C. R. GILLIS, *Cancer Surveillance Unit, Ruchill Hospital, Glasgow.*

Aspects of aetiology, management and survival in ovarian cancer have been examined in a prospective study of all cases first diagnosed in 1974 and resident in the geographical area defined as the West of Scotland (population 3 million). Ovarian cancer, with an overall incidence of 11 cases per $10^5$ females, exhibits varying age-specific rates and aetiological factors, when divided into distinct tumour types. Germcell tumours occur predominantly at the younger ages, while mucinous tumours are related specifically to zero and low parity. Mucinous tumours also tend to present at the earlier stages, and are more amenable to complete surgical removal, whereas undifferentiated tumours prove to be the most difficult to treat.

Study of the management of ovarian cancers shows that for early disease cases, complete removal is effected in 60% of cases and this is usually combined with radiotherapy or, alternatively, no additional treatment is given. Incomplete removal is most commonly combined with chemotherapy. For extensive disease, 67% had no surgical intervention and of these 52% received chemotherapy and 43% received no other treatment.

The FIGO staging of cases with malignant cells in the fluid as stage Ic, is questioned, as they have a 2-year survival of 22%, which is only marginally better than that for Stages III and IV. The amount of tumour removed is the best single factor associated with a good survival, whereas age, pathology and the different methods of treatment showed little correlation. A baseline has now been established for comparison with results for later years and a methodological study has been made on quality-of-life parameters.